# Using Phenotype MicroArrays to Determine Culture Conditions That Induce or Repress Toxin Production by *Clostridium difficile* and Other Microorganisms

**DOI:** 10.1371/journal.pone.0056545

**Published:** 2013-02-20

**Authors:** Xiang-He Lei, Barry R. Bochner

**Affiliations:** Biolog, Inc., Hayward, California, United States of America; Institute Pasteur, France

## Abstract

Toxin production is a central issue in the pathogenesis of *Clostridium difficile* and many other pathogenic microorganisms. Toxin synthesis is influenced by a variety of known and unknown factors of genetics, physiology, and environment. To facilitate the study of toxin production by *C. difficile*, we have developed a new, reliable, quantitative, and robust cell-based cytotoxicity assay. Then we combined this new assay with Phenotype MicroArrays (PM) technology which provides high throughput testing of culture conditions. This allowed us to quantitatively measure toxin production by *C. difficile* type strain ATCC 9689 under 768 culture conditions. The culture conditions include different carbon, nitrogen, phosphorus, and sulfur sources. Among these, 89 conditions produced strong toxin induction and 31 produced strong toxin repression. Strong toxin inducers included adenine, guanosine, arginine dipeptides, γ-D-Glu-Gly, methylamine, and others. Some leucine dipeptides and the triple-leucine tripeptide were among the strongest toxin repressors. While some results are consistent with previous observations, others are new observations that provide insights into toxin regulation and pathogenesis of *C. difficile*. Additionally, we have demonstrated that this combined assay technology can be applied broadly to a wide range of toxin producing microorganisms. This study is the first demonstration of simultaneous assessment of a large number of culture conditions influencing bacterial toxin production. The new functional cytotoxin quantitation method developed provides a valuable tool for studying toxigenic microorganisms and may also find applications in clinical and epidemiological research.

## Introduction

Phenotype MicroArrays (PM) technology provides a simple tool for testing microbial cells [1], [2], [3] as well as mammalian cells [4] under hundreds or thousands of culture conditions. In 2009, Gardiner and colleagues [5] reported on the use of PM to examine the effect of many culture condition variables on toxin production by the pathogenic fungus, *Fusarium graminearum*. This fungus is a major pathogen on wheat, with severe agricultural and commercial impact. Over many decades of study, no one was able to find *in vitro* culture conditions that would turn on synthesis of the *F. graminearum* trichothecene mycotoxin. However, the use of PM technology provided the breakthrough, indicating that strong toxin induction could be obtained *in vitro* by simply culturing the fungus with arginine, putrescine, agmatine, or guanine as the nitrogen source. Secondarily they found and confirmed that pH 4.5 produced additional induction [6].

A different novel approach was taken by Singh [7] from the Natural Products Discovery Group at Wyeth Pharmaceuticals, who studied substrate utilization effects on secondary metabolite production in fungal strains with promising commercial potential. He used the 95 substrates of the FF MicroPlate combined with scaled-down LC-MS to quantitatively profile the secondary metabolites directly from the microwell culture supernatants. Singh showed this to be a promising approach for both characterization and optimization of secondary metabolite production by fungi.

To expand upon and generalize these works, we have undertaken a study of bacterial toxin induction and repression using an important human pathogenic bacterium and incorporating a new and generally applicable toxin detection method.

In 1978, *Clostridium difficile*, a Gram-positive, spore-forming anaerobic bacillus, was identified as a gastrointestinal pathogen that frequently causes diarrhea and more seriously pseudomembranous colitis in patients undergoing antibiotic treatment [8], [9], [10]. Besides diarrhea, the symptoms of *C. difficile* infection (CDI) include abdominal pain, fever, loss of appetite, nausea, toxic megacolon, and even perforations of the colon and sepsis. Death occurred occasionally. With the emergence of hypervirulent strains, the mortality rate of CDI has risen dramatically. Among serious cases, 15,000–20,000 patients die annually from CDI in the United States [11]. This bacterium is also an important animal pathogen [12].

*C. difficile* is a genetically diverse species with a highly dynamic genome that seems to be evolving rapidly [13], [14], [15], [16], [17]. This genetic diversity may be the result of horizontal gene transfer, point mutations, inversions, and large-scale recombination of core chromosomal regions over considerable phylogenetic distance [13], [14], [15], [16]. Disease-causing isolates have arisen not from a single lineage but multiple lineages, suggesting that virulence evolved independently in multiple highly epidemic lineages [13]. These recent findings have provided invaluable insights and significantly advanced our understanding of *C. difficile* pathogenesis and epidemiology.

During the past decade, the prevalence and severity of CDI has increased dramatically worldwide [11], [18], [19], [20], [21], [22]. The emerging epidemic of “hypervirulent” isolates represented by ribotype 027 (also called BI/NAP1/027), which are variant strains of toxinotype III, have been identified as a major culprit in hospital or hospital associated CDI outbreaks [11]. Comparative genomic analyses showed that the epidemic 027 strains have gained 234 additional genes during the past two decades, which may account for their epidemic proficiency and their higher case-fatality ratio [15], [16].

Nevertheless, the central issue in the pathogenesis of *C. difficle* is its major virulence factors, which have long been linked to the two large toxins, A and B. The cause-effect relationship between the toxins and the pathological changes they engender in animal cells, the cytopathic effects (CPE), have been shown to be due to inactivation of Rho-GTPase through glucosylation by the toxins [23], [24], [25], [26]. The essential roles the toxins play in *C. difficile* pathogenesis have also been demonstrated in multiple animal models [27], [28], [29], [30], [31], [32] and in clinical settings [33], [34]. Antibodies against toxins A and B as a supplemental treatment to antibiotic regimens have been shown to reduce recurrence of CDI in patients [35], [36] and to protect intoxicated animals [36]. Identification of *C. difficile* toxin A or B in patients' diarrheal stool is critical and required for diagnosis of CDI [37]. The quality and quantity of the toxins are directly or indirectly determined or regulated by multiple factors such as genetic, environmental, nutritional, and metabolic status. Therefore, monitoring functional toxin production is fundamental in studies of pathogenesis and epidemiology as well as in clinical diagnosis and treatment of CDI.

Cell-based cytotoxicity assay (CCTA) is traditionally regarded as the gold standard assay for *C. difficile* cytotoxin and serves as the reference for other toxin assay methods [38]. This assay looks for toxin induced CPE by microscopic detection of a shift from normal to “rounded” morphology using a toxin-sensitive adherent mammalian cell line (an indicator cell, e.g., CHO, Vero, HT-29, foreskin or others) and then verifies that the CPE is prevented by a specific toxin-neutralizing antibody. This gold standard assay is a true test for functional cytotoxin regardless of whether the DNA coding sequence of the toxin or the sequences of regulatory proteins are mutated. Given that *C. difficile* has an extremely dynamic genome [13], [14], [15], [16], [17], it is critical to have a reference assay that directly tests the toxin's true biological activity. Evidence has shown that, in addition to other factors, virulence is dependent on the autoactivation of a toxin cysteine protease [39], [40], [41], [42]. It is also dependent on the ability of the infected host cell to S-nitrosylate *C. difficile* toxins, which attenuates virulence by inhibiting toxin self-cleavage and cell entry [43]. Toxin B from hypervirulent strains (TcdB_HV_) undergoes acid-induced conformational changes at a pH much higher than that of toxin B from historical strains (TcdB_HIST_), which makes TcdB_HV_ enter the cell more rapidly [44]. Further, TcdB_Hv_ is autoprocessed more efficiently than TcdB_HIST_ [45], which may explain why TcdB_HV_ causes increased cytotoxicity. Understandably, such functional differences of the toxin may not be detected by PCR-based toxin assays, or by ELISA- or Western Blot-based assays which do not necessarily measure toxin function. However, CCTA can detect differences that other methods can not because it is a direct functional and phenotypic assay of toxins.

Although authoritative, traditional CCTA also has some pitfalls, including laborious and time consuming steps (usually 2–3 days for direct toxin assay of a faecal sample, not including the culturing step), difficulties in quantitation due to subjective interpretation (grading cell rounding), and requirements for tissue culture facilities and well trained technical staff [46]. As traditional CCTA is not highly standardized, interpretation of the cytotoxic or cell-rounding activity will be variable [46]. For example, some consider 50% cell rounding a positive reaction [47], [48] while others consider 100% cell rounding a positive reaction [49], making it difficult to compare the results among various laboratories and studies.

We have used a different approach to overcome the inconvenience and limitations of the traditional CCTA assay cited above. To do so, we have taken advantage of Phenotype MicroArray (PM) technology, which can now be employed with both microbial and mammalian cells. In addition to *C. difficile*, we employ a sensitive mammalian indicator cell line (CHO-k1 or Vero cells) as required by CCTA. Combining microbial and mammalian assay methodologies has allowed us to develop an efficient, reliable, highly informative, and quantitative method to measure *C. difficile* toxin production under hundreds to thousands of different culture conditions. Our new hybrid approach enables high throughput evaluation of the role of environmental factors in stimulation or repression of toxin production by *C. difficile*. Given its high sensitivity and reliability, it may potentially be used for direct clinical faecal sample measurement of the toxins. The same approach can be further generalized to study toxins or other secondary metabolites produced by microbial cells.

Here, we report on toxin production of *C. difficile* type strain ATCC 9689 under hundreds of culture conditions, including variations of basic cellular nutritional components for carbon, nitrogen, phosphorus, and sulfur (768 culture conditions, PM1–8). Also, we demonstrate that this same approach is successful with other toxin-producing clostridia as well as toxin-producing aerobic bacteria.

## Results

### Development of *C. difficile* toxin assays in 96-well format

For this study, we purchased purified *C. difficile* toxins A and B which are commercially available in lyophilized form. Once the toxins are dissolved into buffer solutions, their potencies decrease rapidly with noticeable loss by the next day, even if the solutions are stored at 4°C. So, to make standard titration curves for quantification purpose, we used exclusively freshly dissolved purified toxins. We observed that the cytotoxic potency of the supernatants collected from the PM panels also decreased over time. Therefore, the assays of the toxins produced by *C. difficle* strains in PM panels (96-well format) were always carried out with fresh preparations of the supernatants, usually on the same day of collection.

**Figure 1:**
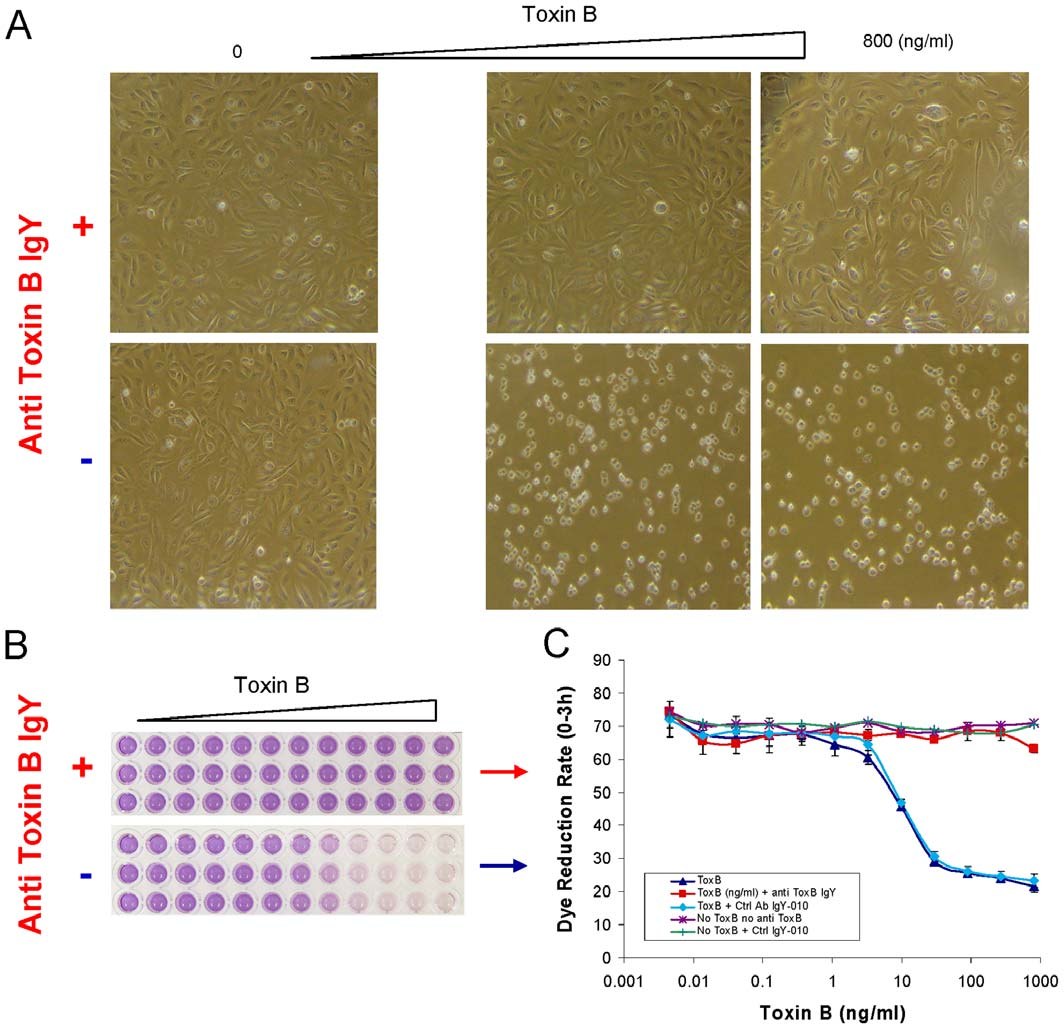
Cytotoxicity and neutralization assay of purified *C. difficile* toxin B with CHO-k1 cell line. Serial 3-fold titrations of standard toxin B (ng/ml) in the presence or absence of 2.5 µg/ml neutralizing antibodies IgY specific for toxin B. (A) CHO-k1 cell morphology changed to rounded shapes by toxin B in a dose dependent manner (the lower panel). This was prevented by the neutralizing antibodies (upper panel). (B) CHO-k1 cell dye reduction was reduced by toxin B also in a dose dependent manner (the lower panel), which corresponds to the cell morphological changes. This effect was also prevented by the neutralizing antibodies (the upper panel). (C) Quantification of the dye reduction changes by toxin B, with (Red) or without (Dark blue) 2.5 µg/ml neutralizing antibodies IgY. Light blue: toxin B with control antibodies IgY-010; Purple: no toxin B with no antibodies; Green: no toxin B with control antibodies IgY-010.

Purified standard *C. difficile* toxin B (Listlab) was used to optimize and calibrate two assays. In a morphological assay the commercial toxin caused cytopathic effects (CPE), seen microscopically as cell rounding changes, in both CHO-k1 and Vero cells. The observed CPE was active in a concentration-dependent manner. The lower panel of Figure 1A shows an example of a toxin B titration with CHO-k1 cells. Purified toxin A (Listlab) was much less potent than toxin B against both cell lines (data not shown).

The CHO-k1 and Vero cells could also be employed in a colorimetric assay using Biolog redox dye MB and an OmniLog instrument to quantitatively measure the degree of intoxication of the cells by the toxin. In this assay also, toxin B-treated cells showed concentration-dependent intoxication of cells that resulted in decreasing rates of color formation as the killed cells were incapable of dye reduction and the dying or injured cells were compromised in the reduction (lower panel of Figure 1B, Figure 1C, Materials and Methods). Therefore, increasing cell rounding in the morphological assay was correlated with decreasing dye reduction rate in the colorimetric assay (Figures 1A, 1B, 1C). Neutralization of toxin B with anti-toxin B polyclonal antibodies IgY (Gallus Immunotech) provided complete protection of the indicator cells in both assays (Figures 1A, 1B, 1C).

The colorimetric dye reduction provided a quantitave assay of toxin over a >3 log concentration range. From serial titrations of standard toxin B, the dye reduction rates by mammalian cells were calculated using PM Analysis Software. Regression analysis on known (prepared) concentrations of toxin B and corresponding dye reduction rates by CHO-k1 cells could be accurately fit to several regression equations over a range of serial titrations of toxin B, from 800 ng/ml (∼2963 pM) down to approximately 0.122 ng/ml (∼0.45 pM). The predicted toxin concentrations calculated from the regression equations were very close to the prepared concentrations (Table 1).

**Table 1:** Predicted concentrations of *C. difficile* toxin compared to the true concentrations in cell culture assay medium.

Prepared toxin Conc. (ng/ml)^a^	Dye Reduction Rate (x)^b^	Regression Equation	R^2^	Predicted Toxin Conc. (ng/ml) (y)^c^
800.000	28.715	y = 10.488x^2^−784.98x+14692	0.998	799.193
266.667	32.365	*ibid.*	*ibid.*	272.231
88.889	34.955	*ibid.*	*ibid.*	67.808
29.630	35.400	y = 7E+08x^−4.7608^	0.980	29.553
9.877	44.945	*ibid.*	*ibid.*	9.484
3.292	60.030	*ibid.*	*ibid.*	2.391
1.097	68.310	y = 5E+62x^−34.157^	0.999	1.093
0.366	70.650	*ibid.*	*ibid.*	0.346
0.122	72.845	*ibid.*	*ibid.*	0.122

a Standard purified *C. difficile* B toxin.

b Dye reduction rate by CHO-k1 cells incubated with the standard purified *C. difficile* toxin B for 20 hr. The dye reduction rate is calculated by using PM Analysis Software from 0 h to 3 h of incubation with Dye MB.

c Predicted *C. difficile* toxin B concentration calculated using the equation indicated in the table.

**Figure 2:**
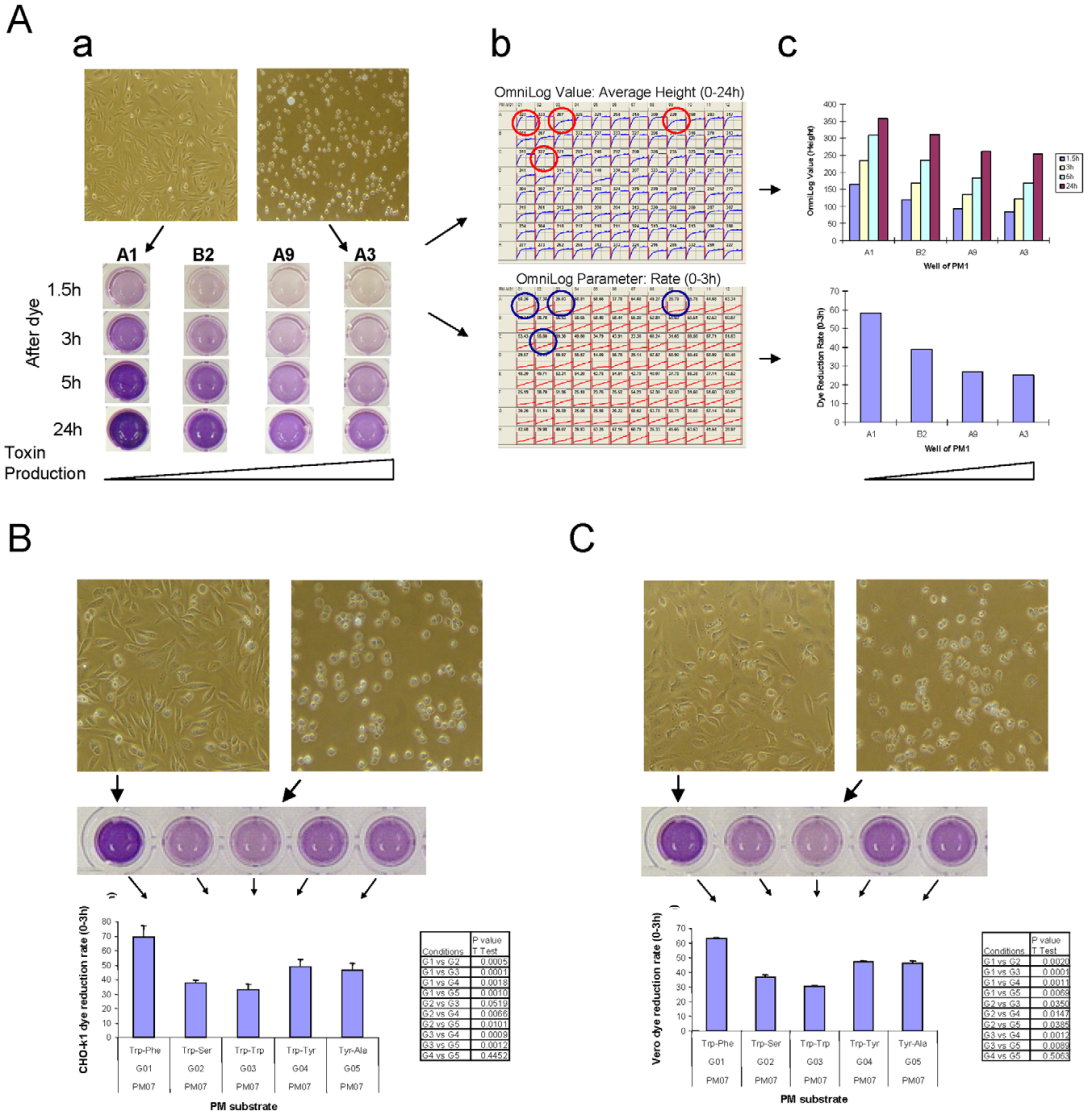
Cellular dye reduction is correlated to cell morphology changes induced by *C. difficile* toxin produced from PM culture conditions. A. CHO-k1 cells used in the assay. (a) Upper part: CHO-k1 cell morphological changes 20 h after exposure to *C. difficile* supernatants collected from different wells of PM1. A1 = No PM substrate control; A3 = N-Acetyl-D-Glucosamine; A9 = D-Alanine; B2 = D-Sorbitol. Lower part: corresponding cellular dye reduction by CHO-k1 cells. Pictures taken at the time points indicated after dye addition. (b) Upper panel: cellular dye reduction kinetics over the course of 24 h, automatically recorded by the OmniLog instrument. The numbers are averages of OmniLog Value (OmniLog Unit or Height); Lower panel: dye reduction rate over 0–3 h, an OmniLog parameter calculated and analyzed by PM Analysis Software. Larger rate numbers indicate faster dye reduction, and therefore healthier cells. (c) Upper panel: plots for OmniLog Values; Lower panel: plots for dye reduction rate. B. Similarities of changes seen with both CHO-k1 (left) and Vero (right) cells in cytotoxicity and dye reduction rate by *C. difficile* supernatants from individual wells of PM7.

**Figure 3:**
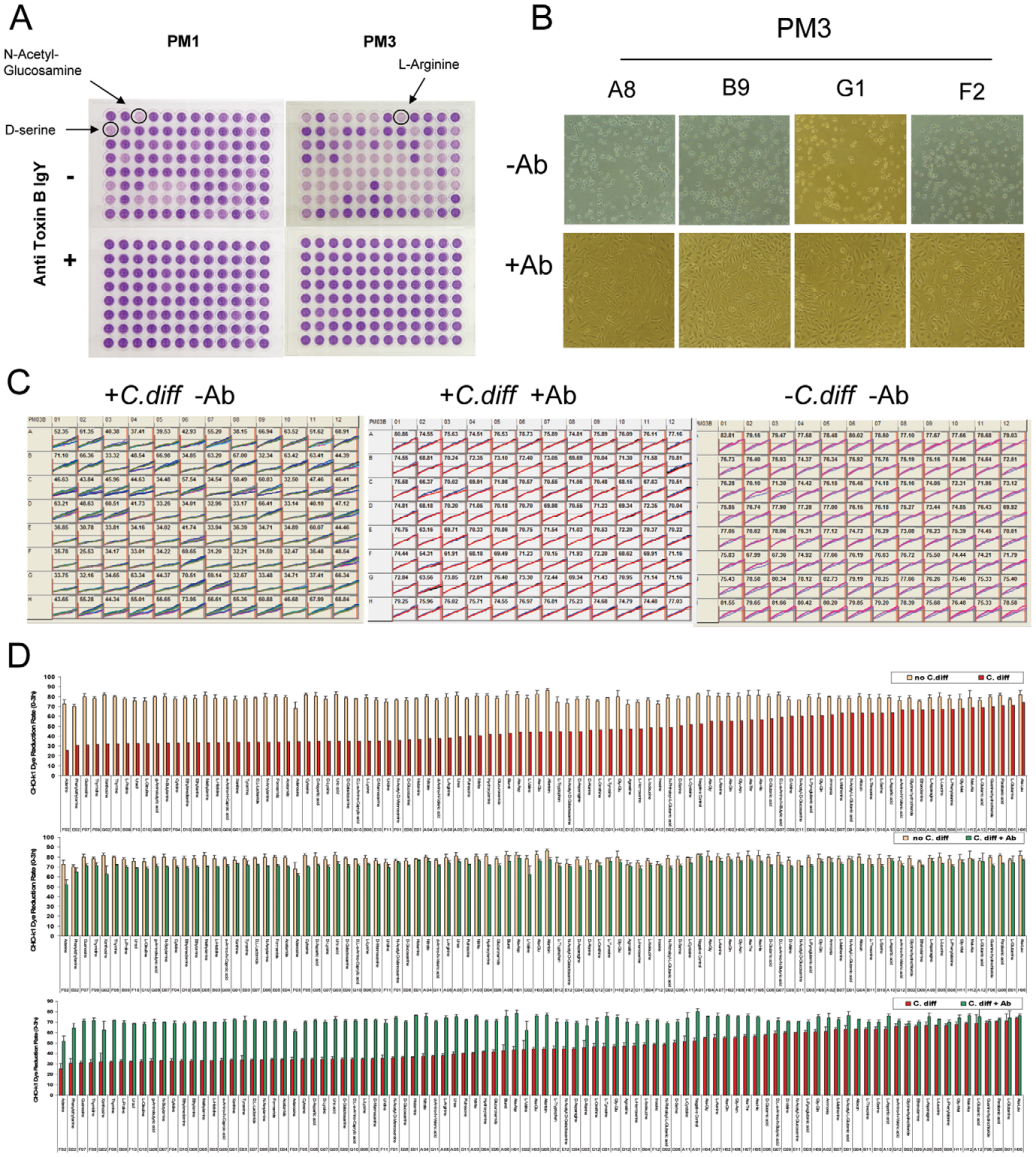
Cytotoxicity and neutralization assays of toxin prepared from *C. difficile* ATCC 9689 grown in PMs. Anti-toxin B polyclonal antibodies IgY were used throughout at 2.5 µg/ml for neutralization assays. A. Representative cell-based cytotoxicity and neutralization assays with redox dye MB in PMs 1 and 3 with CHO-k1 cells. B. Typical CHO-k1 cell morphological changes with or without anti-toxin B IgY. PM3: A8 = L-Arginine, B9 = L-Proline, G1 = Xanthine, F2 = Adenine. C. Dye reduction signals by CHO-k1 cells automatically and kinetically collected by OmniLog instrument. The numbers shown are means of dye reduction rates of replicas of each well in PM3, calculated by PM Analysis Software. D. Histograms of the same data of the dye reduction rates as in C. Tan: no *C. difficile*; Red: *C. difficile*; Green: *C. difficile*+anti-toxin B IgY.

Finally, we showed that toxin preparations taken directly from *C. difficile* cultures could intoxicate indicator cells and be effectively neutralized by anti-toxin B antibody. Both CHO-k1 and Vero cells were intoxicated by crude toxin preparations of *C. difficile* strain ATCC 9689 supernatants from different culture conditions of the PM panels (Figure 2B). The morphological changes of the CHO-k1 cells were identical to those caused by purified standard toxin B (Figures 1A, 2A, 2B, 3B). However, the levels of toxin production in the supernatants were distinctively different under different PM culture conditions. This is clearly shown both by the cell culture toxicity assay (Figures 2A, 2B, 3B) and by the dye reduction assay with the indicator cells (Figures 2, 3A, 3C, 3D). As seen in the case of purified standard toxin B (Figure 1), the inverse correlation between the CPE and dye reduction rate of the intoxicated cells was also observed with unpurified toxin preparations (filtered supernatants) from various PM culture conditions (Figure 2). These adverse effects of unpurified toxin preparations on cell morphology and dye reduction were also specifically prevented by anti-toxin B IgY polyclonal antibodies (Figures 3A, 3B, 3C, 3D).

The cell mass under most PM culture condtions peaked at 24 h and thereafter tended to decrease at 48 h and further decrease at 72 h (Materials and Methods, Figure S1). In contrast, initial experiments indicated that toxin production was low at 24 h and tended to peak around 72 h.

### Toxin production under different PM culture conditions

To examine the effect of culture conditions on toxin production, *C. difficile* ATCC 9689 was cultured in PM1–8 which constitutes 768 culture conditions with variations in the basic metabolic nutrients, C, N, P, and S (Table S1) for 72 h before toxin-containing supernatants were collected. CHO-k1 cells treated with the 768 *C. difficile* supernatants showed different degrees of CPE (Figures 2A, 2B, 3B). Control experiments (no *C. difficile* control) were also performed to show that the 768 chemicals themselves did not induce toxicity to the CHO-k1 cells (data not shown). Based on statistical analysis (t-test) we consider the differences of dye reduction rate between the two groups (*C. difficile* supernatant group and no *C. difficile* control group) to be significant only if P<0.05. There were 544 out of 768 culture conditions that significantly induced *C. difficile* toxin production (Table 2). Of these 544 culture conditions, 89 (16.4% of 544) induced toxin production at high levels of  = >420 ng/ml (Tables 2, 3, S2), 192 (35.3% of 544) gave a middle range level of  = >42 but <420 ng/ml (Tables 2, 4, S2), and 262 (48.2% of 544) gave a low range level of toxin production, >2.562 but <42 ng/ml in the supernatants (Table 2). There were 224 out of 768 culture conditions that were not statistically significant in inducing toxin production (P>0.05 except one PM7 A4 Leu-Trp, P = 0.048). Of these 224, 30 (13.8% of 224) produced lowest toxin levels (<0.122 ng/ml in CHO-k1 cell assay medium, or possibly 0 ng/ml) (Tables 2, 5, S2).

**Table 2:** *C. difficile* toxin production ranges and statistics under different culture conditions from PM1–8.

Toxin concentration range (ng/ml)^a^	Number of PM conditions (P<0.05^b^)	Number of PM conditions (P>0.05^b^)	Total
> = 420	89	0	89
> = 42, <420	192	10	202
>2.562, <42	262	184	446
<0.122^c^	1	30	31
Total	544	224	768

a For measurable toxin levels, the numbers indicate toxin concentrations in supernatants of *C. difficile* grown under different PM conditions.

b The P values were obtained from t-test on the dye reduction rates by CHO-k1 cells in the presence vs absence of *C. difficile* supernatants collected from different PM conditions.

c Below the measurable limit in CHO-k1 cell assay medium of 0.122 ng/ml. Note that the concentration of the toxin in the cell assay medium is 21-fold diluted from the bacterial supernatant (see Materials and Methods).

**Table 3:** PM substrates giving highest levels of toxin production by *C. difficile* ATCC 9689.

Plate panel	Well	Chemical	Category	*C. difficile* Mass^a^	Toxin (ng/ml)^b^	P value^c^
PM03	F02	Adenine	nucleobase	0.031	>16800	2.10E-04
PM03	F07	Guanosine	nucleoside	0.0092	10402	2.45E-05
PM06	B07	Arg-Asp	dipeptide	0.0444	10371	2.32E-07
PM08	G02	γ-D-Glu-Gly	dipeptide, γ-	0.0519	10122	3.34E-03
PM06	B05	Arg-Ala	dipeptide	0.0456	10080	5.70E-08
PM03	G02	Xanthosine	nucleoside	0.0516	9499	6.36E-09
PM06	B08	Arg-Gln	dipeptide	0.0476	8912	4.54E-08
PM06	C05	Arg-Tyr	dipeptide	0.0455	8819	7.07E-05
PM06	F10	His-Pro	dipeptide	0.0481	8568	6.08E-07
PM06	C04	Arg-Trp	dipeptide	0.0499	8423	7.81E-07
PM08	F04	β-Ala-His	dipeptide, β-	0.0473	8265	3.15E-06
PM06	B06	Arg-Arg	dipeptide	0.0435	8229	4.65E-07
PM03	F08	Thymine	nucleobase	0.0338	7749	2.83E-09
PM06	C03	Arg-Ser	dipeptide	0.0448	7608	1.85E-08
PM07	D08	Pro-Hyp	dipeptide	0.045	7523	1.04E-06
PM03	F09	Thymidine	nucleoside	0.0384	7336	3.22E-08
PM07	E07	Ser-Pro	dipeptide	0.0472	7136	2.23E-07
PM06	F08	His-Lys	dipeptide	0.0453	6993	1.02E-07
PM06	C02	Arg-Phe	dipeptide	0.0467	6541	2.70E-07
PM03	D05	Methylamine	amine	0.0398	6537	1.61E-04
PM07	F07	Trp-Arg	dipeptide	0.0628	6468	9.53E-07
PM03	G08	γ-Aminobutyric acid	amino fatty acid, γ-, GABA	0.0375	6329	4.45E-11
PM03	D07	N-Butylamine	amine, N-	0.0407	5679	3.74E-04
PM07	A07	Lys-Arg	dipeptide	0.0502	5536	5.20E-06
PM03	B09	L-Proline	amino acid	0.0394	5343	1.07E-10
PM07	B04	Lys-Trp	dipeptide	0.006	5277	5.29E-06
PM07	B02	Lys-Ser	dipeptide	0.0426	5105	1.25E-07
PM03	F05	Cytosine	nucleobase	0.0361	4809	1.11E-09
PM07	F05	Thr-Pro	dipeptide	0.0355	4772	3.32E-06
PM03	G03	Uric acid	nucleobase derivative	0.0556	4289	6.27E-07
PM03	D10	Ethylenediamine	amine	0.0369	4133	2.78E-05
PM07	G03	Trp-Trp	dipeptide	0.055	4104	1.74E-03
PM06	B09	Arg-Glu	dipeptide	0.0404	4059	1.70E-09
PM03	F04	Cytidine	nucleoside	0.0456	3952	9.78E-12
PM03	D08	Ethylamine	amine	0.0401	3799	8.84E-05
PM06	E11	Gly-Pro	dipeptide	0.0414	3798	9.96E-07
PM03	B03	L-Histidine	amino acid	0.0361	3640	8.27E-05
PM03	E05	Formamide	amide	0.0418	3639	1.96E-11
PM06	F02	Gly-Trp	dipeptide	0.0431	3632	1.68E-05
PM03	G09	ε-Amino-N-Caproic acid	amino fatty acid, ε-	0.0366	3389	4.02E-05
PM03	D06	N-Amylamine	amine, N-	0.0433	3384	9.99E-09
PM03	C05	D-Aspartic acid	amino acid, D-	0.0109	3334	3.33E-04
PM03	F10	Uracil	nucleobase	0.0375	3204	2.32E-04
PM03	C10	L-Citrulline	amino acid	0.0377	3086	1.56E-03
PM08	C11	Pro-Arg	dipeptide	0.0442	2967	2.61E-03
PM03	E04	Acetamide	amide	0.0458	2926	7.25E-09
PM03	E07	D,L-Lactamide	amide, DL-	0.04	2859	1.50E-07
PM03	E03	Tyramine	amine, Tyr derivative	0.0442	2772	9.91E-07
PM01	F04	D-Threonine	amino acid, D-	0.0923	2699	1.04E-03
PM01	A03	N-Acetyl-D-Glucosamine	acetyl amino sugar, N-	0.0955	2657	5.17E-05
PM01	G05	L-Alanine	amino acid	0.113	2469	1.02E-04
PM01	F05	Fumaric Acid	carboxylic acid	0.018	2284	8.73E-04
PM03	E02	β-Phenylethylamine	amine, Phe derivative, β-	0.0448	2147	1.80E-06
PM01	G06	Ala-Gly	dipeptide	0.0934	2130	4.82E-07
PM03	G01	Xanthine	nucleobase	0.2978	2079	2.83E-05
PM08	C01	Lys-Gly	dipeptide	0.047	1950	1.30E-04
PM08	H03	Gly-Gly-Gly	tripeptide	0.0456	1811	2.93E-05
PM03	B06	L-Lysine	amino acid	0.0436	1767	3.75E-04
PM03	E09	D-Galactosamine	amino sugar, D-	0.0374	1723	3.43E-09
PM08	F03	β-Ala-Gly	dipeptide, β-	0.0389	1547	2.32E-07
PM01	G03	L-Serine	amino acid	0.0635	1537	4.48E-03
PM08	G04	Gly-D-Asp	dipeptide	0.0467	1537	3.45E-07
PM01	F01	Gly-Asp	dipeptide	0.1004	1507	3.62E-03
PM03	C07	D-Lysine	amino acid, D-	0.0489	1472	1.00E-04
PM03	G10	D,L-α-Amino-Caprylic acid	amino fatty acid, DL-α	0.0484	647	4.73E-07
PM01	F06	Bromosuccinic Acid	carboxylic acid	0.0335	615	8.93E-05
PM01	B01	D-Serine	amino acid, D-	0.1114	588	1.19E-03
PM02	G07	L-Homoserine	amino acid, Thr isomer	0.0071	587	7.04E-03
PM03	E10	D-Mannosamine	amino sugar, D-	0.0364	583	1.06E-05
PM08	G12	D-Ala-Gly-Gly	tripeptide	0.0538	515	1.09E-05
PM02	G10	L-Leucine	amino acid	0.089	503	2.97E-05
PM03	E01	Histamine	amine, His derivative	0.0424	492	3.79E-08
PM03	F11	Uridine	nucleoside	0.0373	484	2.62E-04
PM03	E08	D-Glucosamine	amino sugar, D-	0.0447	476	3.86E-06
PM03	A08	L-Arginine	amino acid	0.046	458	2.65E-05
PM03	G11	δ-Amino-N-Valeric acid	amino fatty acid, δ-	0.0424	435	5.08E-10
PM1–8	All wells	All substrates	control	0.0517	50	3.62E-02
PM1–8	A1	No substrate	control	0.0517	44	9.80E-02

a OD (750 nm) difference between *C. difficile* under certain PM substrate and the same substrate without *C. difficile*.

b Toxin concentrations in *C. difficile* supernatant collected from different PM conditions, which were calculated from the average dye reduction rate by the CHO-k1 cells according to the equations in Table 1.

c The P values were obtained from t-test on the dye reduction rates of CHO-k1 cells in the presence or absence of *C. difficile* supernatants collected from different PM conditions.

**Table 4:** PM substrates giving middle levels of toxin productions by *C. difficile* ATCC 9689.

Plate panel	Well	Chemical	Category	*C. difficile* Mass^a^	Toxin (ng/ml)^b^	P value^c^
PM07	F06	Trp-Ala	dipeptide	0.0489	415	1.08E-04
PM03	A03	Nitrite	inorganic N-source	0.0333	383	3.24E-07
PM08	B08	Leu-Asn	dipeptide	0.0482	381	2.24E-03
PM06	F07	His-Leu	dipeptide	0.0495	376	6.77E-05
PM03	F03	Adenosine	nucleoside	0.0472	373	7.02E-03
PM01	G01	Gly-Glu	dipeptide	0.0411	366	5.53E-04
PM06	E03	Gly-Arg	dipeptide	0.0403	352	6.87E-05
PM08	B12	Lys-Asp	dipeptide	0.0525	335	4.93E-05
PM03	D11	Putrescine	amine	0.0339	335	7.69E-08
PM03	G05	Allantoin	nucleobase derivative	0.0425	334	1.48E-08
PM03	D04	Hydroxylamine	inorganic base, reducing agent	0.0228	334	1.58E-07
PM01	D02	D-Aspartic Acid	amino acid, D-Asp	0.0269	331	5.56E-05
PM02	B02	N-Acetyl-Neuraminic acid	acetyl amino sugar, sialic acid	0.1163	328	1.77E-03
PM04	C12	Cytidine 3′,5′-Cyclic Monophosphate	nucleotide, 3′,5′-cyclic	0.0106	321	8.83E-05
PM03	A06	Biuret	amide, carbamide derivative	0.0394	307	3.59E-06
PM01	G10	Methylpyruvate	carboxylic acid derivative, methyl ester	0.0795	299	5.31E-05
PM08	H08	Gly-Phe-Phe	tripeptide	0.2751	298	4.18E-07
PM08	G05	Gly-D-Ser	dipeptide	0.0543	297	8.49E-06
PM08	H07	Val-Tyr-Val	tripeptide	0.0382	296	2.62E-06
PM03	E06	Glucuronamide	sugar acid, amide (6c)	0.0369	280	1.10E-06
PM07	A08	Lys-Glu	dipeptide	0.0425	271	1.01E-04
PM01	G04	L-Threonine	amino acid, Thr	0.0643	243	3.70E-03
PM07	H05	Val-Asp	dipeptide	0.0469	225	1.12E-04
PM03	C02	L-Valine	amino acid, Val	0.0431	223	2.01E-04
PM08	H05	Gly-Gly-Leu	tripeptide	0.0305	215	1.03E-04
PM02	B08	Arbutin	sugar hydroquinone (12c), glycoside	0.128	213	5.36E-05
PM03	C04	D-Asparagine	amino acid, D-Apn	0.0321	196	2.31E-04
PM07	E04	Ser-Leu	dipeptide	0.0404	187	3.43E-04
PM07	G05	Tyr-Ala	dipeptide	0.0331	186	5.79E-05
PM03	H10	Gly-Glu	dipeptide	0.0472	184	5.43E-03
PM08	E03	Trp-Val	dipeptide	0.0276	184	1.26E-04
PM08	E12	Val-Pro	dipeptide	0.0425	183	2.01E-04
PM06	F12	His-Trp	dipeptide	0.0442	178	1.49E-04
PM08	E08	Val-Glu	dipeptide	0.0481	178	1.11E-05
PM07	H02	Tyr-Tyr	dipeptide	0.0415	174	2.16E-04
PM01	H12	2-Aminoethanol	amine, alcohol, 2-	0.0853	173	1.15E-03
PM03	C01	L-Tyrosine	amino acid, Tyr	0.1528	172	1.52E-05
PM03	C03	D-Alanine	amino acid, D-Ala	0.038	171	1.70E-04
PM03	B12	L-Tryptophan	amino acid, Trp	0.0299	165	2.91E-04
PM02	E01	Capric acid	fatty acid	0.014	162	3.24E-04
PM02	A06	Dextrin	sugar, polysaccharide	0.0478	162	3.99E-03
PM08	G06	Gly-D-Thr	dipeptide	0.0549	157	3.21E-05
PM03	E12	N-Acetyl-D-Galactosamine	acetyl amino sugar, N-	0.0355	153	3.06E-04
PM07	A11	Lys-Lys	dipeptide	0.0427	150	5.07E-05
PM03	C12	L-Ornithine	amino acid, Orn	0.0369	141	3.49E-04
PM02	D02	Salicin	sugar phenol, β-glucoside (13c)	0.1291	140	3.61E-03
PM02	C04	D-Lyxose	sugar (5c), D-	0.1508	139	3.82E-03
PM03	D02	N-Phthaloyl-L-Glutamic acid	amino acid derivative	0.0344	137	4.69E-05
PM02	H04	L-Valine	amino acid, Val	0.0917	135	1.28E-02
PM07	G04	Trp-Tyr	dipeptide	0.0451	134	5.89E-05
PM02	E02	Caproic acid	fatty acid	0.0568	133	1.90E-03
PM02	G12	L-Methionine	amino acid, Met	0.0818	127	6.01E-04
PM06	G04	Ile-Arg	dipeptide	0.0442	122	3.82E-03
PM03	A01	Negative Control	negative control	0.0403	122	1.90E-04
PM03	B04	L-Isoleucine	amino acid, Ile	0.0563	122	9.75E-04
PM02	D06	D-Tagatose	sugar (6c), D-	0.138	121	8.44E-04
PM03	C11	L-Homoserine	amino acid, Thr isomer	0.0293	119	2.52E-05
PM01	A09	D-Alanine	amino acid, D-Ala	0.1233	118	9.72E-06
PM07	A03	Leu-Ser	dipeptide	0.037	116	1.51E-04
PM03	C08	D-Serine	amino acid, D-Ser	0.0465	110	7.29E-05
PM03	A11	L-Cysteine	amino acid, Cys	0.0394	108	6.64E-05
PM03	D12	Agmatine	amine, Arg derivative	0.043	108	3.06E-04
PM01	C07	D-Fructose	sugar (6c), D-	0.1435	106	1.34E-06
PM01	H08	Pyruvic Acid	carboxylic acid, α-keto acid	0.093	103	5.67E-04
PM02	H02	L-Phenylalanine	amino acid, Phe	0.0714	103	2.46E-02
PM1–8	All wells	All substrates	control	0.0517	50	3.62E-02
PM1–8	A1	No substrate	control	0.0517	44	9.80E-02

a OD (750 nm) difference between *C. difficile* under certain PM substrate and the same substrate without *C. difficile*.

b Toxin concentrations in C. difficile supernatant collected from different PM conditions, which were calculated from the average dye reduction rate by the CHO-k1 cells according to the equations in Table 1.

c The P values were obtained from t-test on the dye reduction rates of CHO-k1 cells in the presence or absence of *C. difficile* supernatants collected from different PM conditions.

**Table 5:** PM substrates giving lowest levels of toxin production by *C. difficile* ATCC 9689.

PM panel	Well	Chemical	Category	*C. difficile* mass^a^	Toxin (ng/ml)^b^	P value^c^
PM01	H11	β-Phenylethylamine	amine, Phe derivative, β-, (C-source)	0.0477	<0.122	9.40E-01
PM02	H11	2,3-Butanone	ketone, methyl ethyl, 2,3-, C-source)	0.0572	<0.122	4.87E-01
PM04	A01	Negative Control	ctrl, no substrate	0.0473	<0.122	2.59E-01
PM04	A10	Adenosine 5′-Monophosphate	nucleotide, (P-source)	0.0495	<0.122	8.02E-02
PM04	A03	Pyrophosphate	diphosphate, (P-source)	0.0075	<0.122	1.98E-01
PM04	A04	Trimetaphosphate	metaphosphate, (P-source)	0.0493	<0.122	1.86E-01
PM04	B01	Thiophosphate	thiophosphate (P-source)	0.0373	<0.122	1.92E-01
PM04	H04	D,L-Lipoamide	amide, 6,8-dithiooctanoic, DL-, (S-source)	0.0265	<0.122	1.16E-01
PM04	G12	L-Methionine Sulfone	amino acid derivative (S-source)	0.0446	<0.122	3.84E-01
PM04	H06	Taurine	aminoethanesulfonic acid, 2-, (S-source)	0.0505	<0.122	1.76E-01
PM04	H05	Taurocholic acid	cholyltaurine (S-source)	0.0536	<0.122	1.64E-01
PM04	H08	p-Aminobenzene Sulfonic acid	sulfanilic acid, p-, (S-source)	0.0485	<0.122	1.64E-01
PM04	H07	Hypotaurine	sulfinic acid, (S-source)	0.0438	<0.122	3.83E-01
PM04	H12	Tetramethylene Sulfone	Sulfone, (S-source)	0.0442	<0.122	6.03E-01
PM04	H09	Butane Sulfonic acid	sulfonic acid derivative, (S-source)	0.0398	<0.122	3.37E-01
PM04	H10	2-Hydroxyethane Sulfonic acid	sulfonic acid derivative, 2- (S-source)	0.0428	<0.122	2.03E-01
PM05	A11	Adenosine	nucleoside (nutritional stimulant)	0.0414	<0.122	5.88E-01
PM06	A12	Ala-Pro	dipeptide (N-source)	0.0507	<0.122	2.44E-01
PM08	H04	Gly-Gly-Ile	tripeptide (N-source)	0.0421	<0.122	1.38E-01
PM06	E12	Gly-Ser	dipeptide (N-source)	0.0471	<0.122	3.13E-01
PM06	H09	Leu-Ile	dipeptide (N-source)	0.0523	<0.122	8.95E-02
PM06	H10	Leu-Leu	dipeptide (N-source)	0.0530	<0.122	1.06E-01
PM08	H10	Leu-Leu-Leu	tripeptide (N-source)	0.0463	<0.122	1.24E-01
PM06	H11	Leu-Met	dipeptide (N-source)	0.0501	<0.122	1.12E-01
PM06	H12	Leu-Phe	dipeptide (N-source)	0.0538	<0.122	7.52E-02
PM07	A04	Leu-Trp	dipeptide (N-source)	0.0380	<0.122	4.79E-02
PM07	A02	L-Glutamine	amino acid (N-source)	0.0339	<0.122	7.04E-02
PM07	A12	Lys-Phe	dipeptide (N-source)	0.0533	<0.122	6.40E-02
PM08	D12	Thr-Gln	dipeptide (N-source)	0.0403	<0.122	7.74E-02
PM07	G12	Tyr-Phe	dipeptide (N-source)	0.0559	<0.122	7.37E-02
PM08	F01	Val-Ser	dipeptide (N-source)	0.0455	<0.122	6.23E-01
PM6–8	A1	No PM substrate	Control	0.0489	2.690	9.80E-02
PM6–8	All Wells	All PM substrates	Control	0.0467	2.423	3.62E-02
PM6–8	All Wells	All PM substrates but no *C. difficile*	Control	0.0423^d^	<0.122^e^	NA

a OD (750 nm) difference between *C. difficile* under certain PM substrate (condition) and the same substrate without *C. difficile*.

b Toxin concentrations in the CHO-k1 assay medium with *C. difficile* supernatant collected from different PM conditions, which were calculated from the average dye reduction rate by the CHO-k1 cells according to the equations in Table 1.

c The P values were obtained from t-test on the dye reduction rates of CHO-k1 cells in the presence or absence of *C. difficile* supernatants collected from different PM conditions.

d Average OD (750 nm) value of all uninoculated PM substrates from PM1–8.

e This value is a result of calculation based on dye reduction rate of CHO-k1 cells in the presence of PM substrates of PM1–8 without *C. difficile* inoculation. It is a measure of zero toxin.

**Figure 4:**
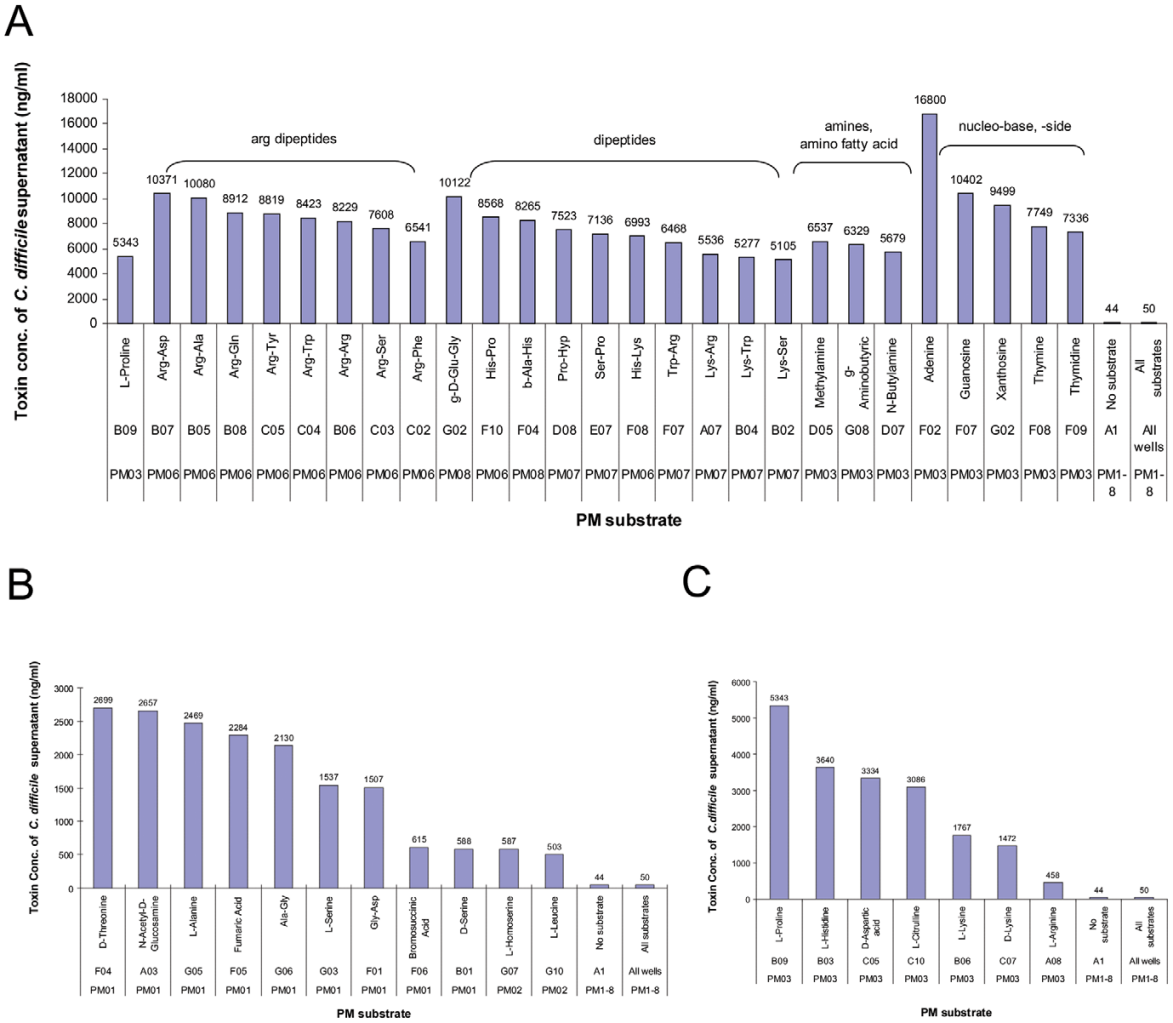
Examples of high toxin-inducing PM substrates for *C. difficile* ATCC 9689. A. Top toxin inducers. Note that adenine as a nitrogen source is the strongest toxin inducer of all tested PM substrates in this study. g-D-Glu-Gly = γ-D-Glu-Gly; b-Ala-His = β-Ala-His; g-Aminobutyric = γ-Aminobutyric acid. B. High toxin production induced by some carbon sources in PM1 and 2. C. High toxin production induced by some amino acids as nitrogen sources. Note that L-proline is the highest toxin inducer in the amino acid category.

With glucose as the carbon source, a variety of nitrogen sources from PM3, 6, 7, and 8 gave highest toxin productions ( = >420 ng/ml) (Figures 4A, 4C, Tables 3, S2). The nitrogen sources were the predominant class of high toxin level inducers compared to other categories (e.g., carbon, phosphorus, sulfur sources, etc.) of PM culture conditions. The most powerful *C. difficile* toxin inducers (>2,100 ng/ml) were found in nucleobases, nucleosides, dipeptides, and amine compounds, which included adenine, guanosine, arginine dipeptides, γ-D-Glu-Gly, methylamine and others (Figure 4A, Tables 3, S2). Among amino acids as nitrogen sources, L-proline, L-histidine, D-aspartic acid, L-citrulline, L-lysine, and D-lysine were also seen in the group of high toxin inducers (Figure 4C, Tables 3, S2). Overall, the highest toxin inducer was adenine as a nitrogen source in PM3, which exceeded the upper tested concentration of the standard purified toxin B in this study (800 ng/ml). This corresponds to 16,800 ng/ml or greater (with a 21-fold dilution factor applied) in the bacterial supernatant (Figure 4A, Table 3). Guanosine as a nitrogen source induced the second highest toxin production of *C. difficile* ATCC 9689 (Figure 4A, Table 3). These were followed by xanthosine, thymine, and thymidine, which induced toxin levels >7,000 ng/ml, and by others including cytosine, cytidine, uracil, uridine, and xanthine. The purine metabolite uric acid also stimulated high toxin production (4288 ng/ml) (Figure 4A, Table 3). Interestingly, whereas multiple arginine dipeptides were among the very top toxin inducers (Figure 4A, Table 3), arginine by itself was not, although it was among the category of high toxin producers (Figure 4C, Table 3). It induced ∼14–22 times lower than the top toxin-inducing arginine dipeptides (Figures 4A, 4C, Table 3). Presumably it is not taken up as well as the arginine-containing peptides or arginine dipeptides as intact molecules work differently than arginine itself in stimulating the toxin production.

Carbon sources from PM1 and 2 gave lower levels of toxin production and were less frequent toxin inducers. Though weaker inducers than the nitrogen sources in PM3,6,7,8, D-threonine, N-acetyl-D-glucosamine, L-alanine, Ala-Gly, fumaric acid, L-serine, and Gly-Asp were the highest toxin inducers of the carbon sources in panels PM1–2 (Figure 4B), and induced toxin levels of greater than 1500 ng/ml. Phosphorus and sulfur sources from PM4 were not seen as significant toxin inducers with *C. difficile* ATCC 9689, although cytidine 3′,5′-cyclic monophosphate as a P-source in PM4 induced a middle range (320 ng/ml) of toxin production. None of the culture conditions in PM5, with low levels of potentially stimulatory nutrients, showed significant toxin induction or repression.

**Figure 5:**
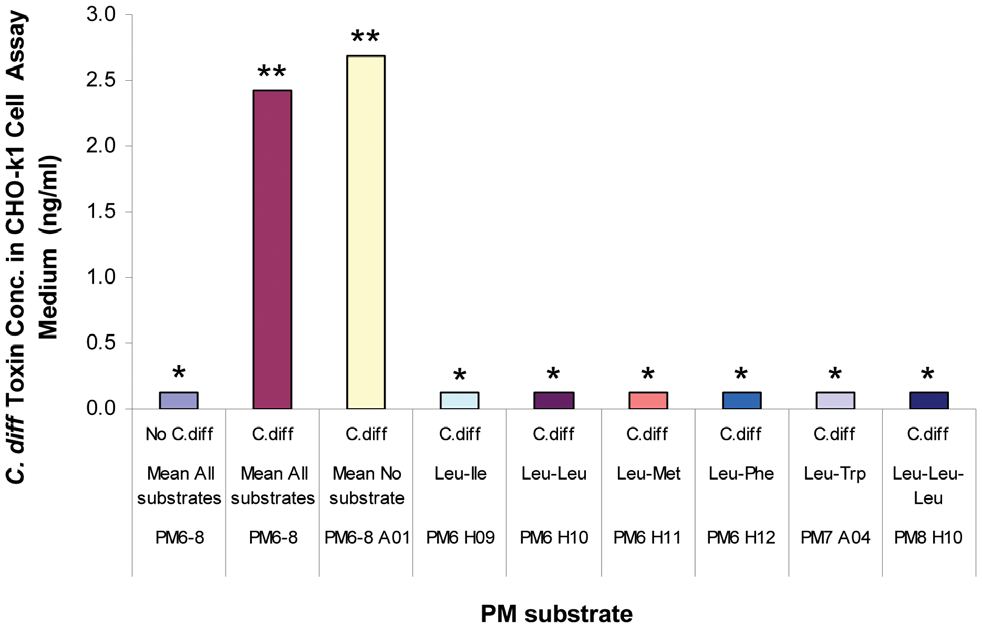
Repression of *C. difficile* toxin production by leucine dipeptides and the triple leucine tripeptide. * Since the dye reduction rate by CHO-k1 cells under the condition was greater than the upper limit indicated in Table 1, the estimated minimum toxin concentration value (0.12 ng/ml) measurable in this assay was taken for plotting purpose, which was actually <0.12 ng/ml or could even be 0 ng/ml. ** The corresponsing dye reduction rates were within the range covered by the equation in Table 1. Therefore, these toxin concentrations were obtained from the rates by calculation. Because all *C. difficile* supernatants were diluted when tested in CHO-k1 cell assay medium, the actual toxin concentrations in the supernatants were all 21 fold higher than the displayed values.

The other interesting class is the PM substrates that gave lowest levels of *C. difficile* toxin production or no toxin at all (<0.122 ng/ml in CHO-k1 assay medium, or correspondingly <2.562 ng/ml in bacterial supernatant), which were indistinguishable from no *C. difficile* control (Table 5). The concentrations were so low that they were out of the reliable range of dye reduction measurement. Notable among this group were several leucine dipeptides and the leu-leu-leu tripeptide (Figure 5, Table 5). Given the average toxin production of 57 ng/ml in the bacterial supernatant (or 2.690 ng/ml in the cell assay medium) from the no PM substrate control and 51 ng/ml (or 2.423 ng/ml in the cell assay medium) from all PM substrate controls of PM 6 through 8 (Figure 5, Table 5), and also given that the bacterial mass under those conditions were all comparable (Table 5), the substrates that gave very low or no toxin production may in fact repress toxin production by *C. difficile*.

Experiments were also performed to compare the results of the type strain *of C. difficile* to the toxinotype strain VPI 10463 (ATCC 43255). These experiments showed that the toxin levels were greatly elevated in the toxinotype strain which we estimate at 100–1000 times higher than ATCC 9689 (data not shown).

**Figure 6:**
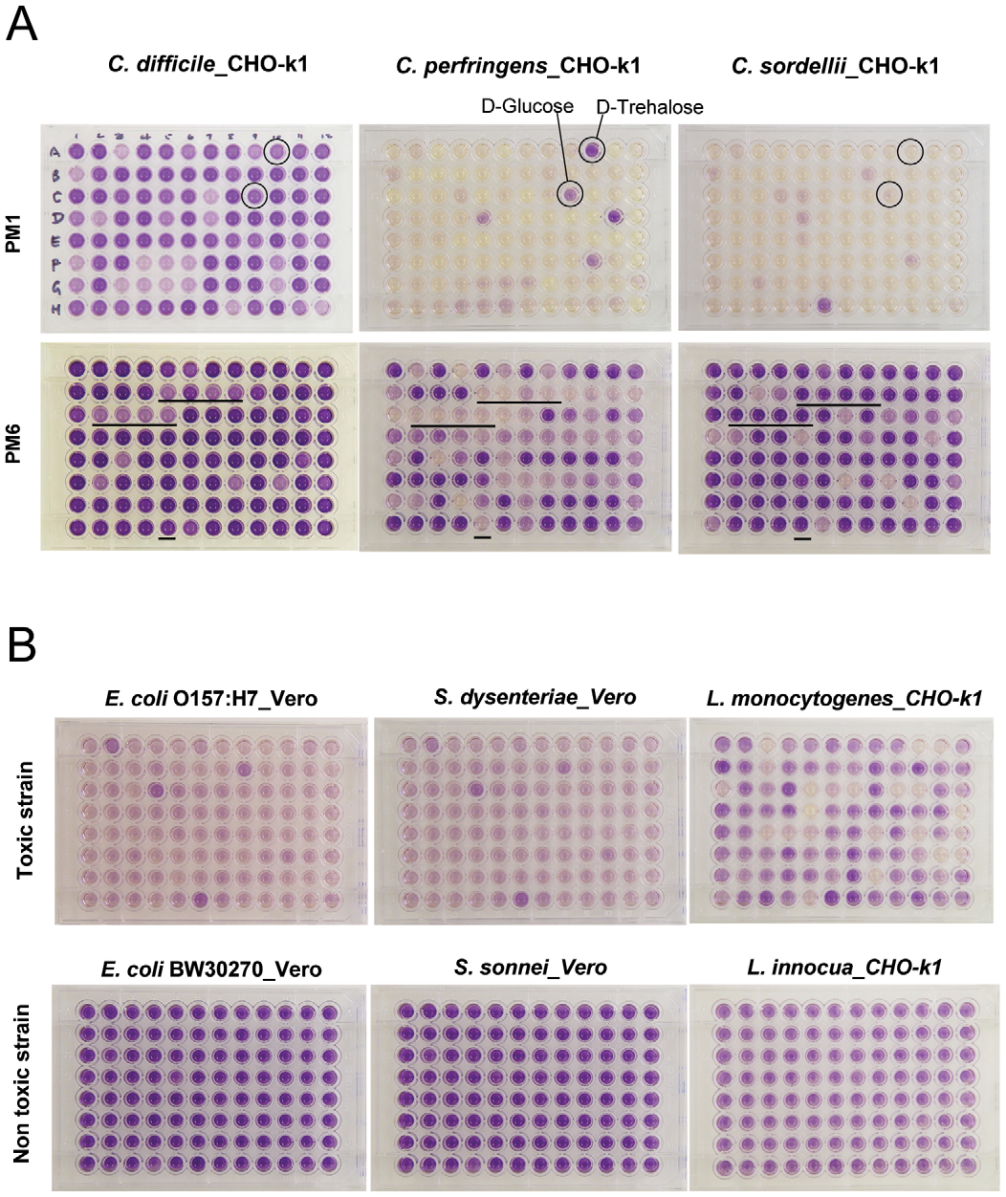
Cell-based cytotoxicity assay of other bacteria. A. Cytotoxin assay comparison of Clostridial species. Five µl of bacterial supernatant from each well of the PM of each Clostridial species, *C. difficile* (ATCC 9689), *C. perfringens* (ATCC 25763), and *C. sordellii* (ATCC 9714), were used in this assay. CHO-k1 is the indicator cell in this dye reduction assay. Upper panel: supernatants from PM1; Lower panel: supernatants from PM6. In the lower panel, the wells above the lines contain arginine dipeptides as the nitrogen source. B. Aerobic bacterial toxin assays and comparison of supernatants from PM1 between toxigenic and non toxigenic strains or species. Vero is the indicator cell for *E. coli* strains and *Shigella* species. CHO-k1 is the indicator cell for *Listeria* species. Upper panel: toxigenic strains or species; Lower panel: non-toxigenic strains or species. For all: darker color indicates higher dye reduction rate and less toxin produced in that well; lighter color indicates lower dye reduction rate and more toxin produced in that well.

Additional experiments were performed to demonstrate the general applicability of this assay technology to diverse toxin producing bacteria. Using CHO-k1 or Vero cells we could demonstrate variable toxicity from the PM panel supernatants of *C. perfringens*, *C. tetani*, *C. sordellii*, *Bacillus cereus*, *Escherichia coli O157* (Vero), *Shigella dysenteriae* (Vero), and *Listeria monocytogenes* (Figure 6).

## Discussion

Reliable, quantitative, and robust assays for functional toxins are essential in scientific research and clinical practice. The roots of our new assay extend back by several decades. In 1983, Mosmann published a highly cited, quantitative and colorimetric assay using the tetrazolium salt MTT to measure mammalian cell proliferation and cytotoxicity [50]. Since then, it has been applied in numerous research studies. In addition to its applications in anti-cancer drug research and other toxicity studies, it has also been applied to bacterial cytotoxin studies [51] including measurement of cytotoxcity of *C. difficile* toxin [43], [52], [53], [54]. Rothman similarly reported quantitation of cell responses to *C. difficile* toxin by crystal violet staining of mammalian cells [55]. The application of colorimetric dyes significantly reduces the labor time because quantitation of color change avoids the laborious and subjective counting of rounded cells. However, the prior colorimetric assays are also inconvenient in that they require solubilization of dye and reading of the plates manually with a microplate reader. This compromises the efficiency of the assays and does not allow one to collect high throughput kinetic data for analysis.

To develop a new, reliable, quantitative, and robust cytotoxicity assay, we started with the gold standard approach by observing the reliable morphological changes of intoxicated cells by *C. difficile* toxin B, the cell rounding, or cytopathic effect (CPE). We confirmed that the cell rounding caused by *C. difficile* toxin B is concentration-dependent and specifically prevented by IgY anti-toxin B polyclonal antibodies. Then, we employed a new proprietary Dye Mix MB [4], that forms a water soluble formazan and does not require solubilization. We also employed the OmniLog instrument which enables the automatic collection of kinetic dye reduction data directly from all wells of 96-well plates in a high throughput format and calculated reduction rates using PM Analysis Software. This enabled measurement of the CPE as decreased cell viability resulting in decreased redox dye reduction rates. Through quantitative analysis, we showed that the CPE is inversely correlated with dye reduction rate of the cells: the stronger the CPE, the lower the dye reduction rate. Furthermore, both toxin-induced CPE and decreased dye reduction rate were specifically and simultaneously prevented by the anti-toxin antibodies. The close correlation between the predicted toxin concentrations calculated from the regression equations and the prepared concentrations (Table 1) indicates that the equations obtained using the standard toxin are reliable and accurate over a wide range (>3 logs) of toxin concentration. Thus, the basis of determining the levels of *C. difficile* toxin production under various culture conditions in this study has been established.

It is estimated that toxin B of *C. difficile* is 1000 times more potent than toxin A [56]. We also observed that the indicator cell lines (CHO-k1 and Vero) were much more sensitive to toxin B than to toxin A (data not shown). Because of this large difference, trace or equivalent amounts of toxin A contamination in toxin B preparations would be of no consequence in the cell rounding assay [56]. Therefore, in this study, we employed exclusively IgY anti-toxin B polyclonal antibodies which were capable of completely protecting the cells from purified standard toxin B (Figure 1). As expected, the anti-toxin B IgY almost completely protected the cells from the crude toxin preparations collected from 96-well PM panels as well (Figures 3A, 3B, 3C, 3D).

As a cell-based cytotoxicity assay, this new method has gold standard reliability and makes the traditional cytotoxicity assay objectively quantifiable, more efficient, shorter in turn-around time (1 day rather than 2 or 3 days), and amenable to high throughput testing. By eliminating subjective scoring of cell rounding, it allows results from different laboratories to be compared.

Combining this toxin assay method with various PM culture conditions (in 96-well plates) provides another unique advantage over the traditional methods used in toxin research. It allows scientists to simultaneously study hundreds to thousands of culture conditions that may positively or negatively affect toxin production by *C. difficile* or other toxigenic microorganisms. Using this approach, we measured toxin production by *C. difficile* ATCC 9689 under very diverse nutritional conditions, including 768 carbon, nitrogen, phosphorus, sulfur, and other nutrient sources (Table S1). Measuring hundreds of culture conditions in a quantitative and high throughput manner provides a broad perspective on toxin regulation and thus increases the probability of meaningful discoveries in toxin research. It provides another dimension beyond the effects of genetic changes.

Research has shown that toxin A and B production by *C. difficile* VPI 10463 is regulated by temperature through TcdR activity, the alternative sigma factor positively regulating *tcdA* and *tcdB* expression [57]. At 37°C, toxin expression was highest compared to 22 or 42°C. This temperature regulated toxin production is positively correlated to the regulation of butyric acid production, but not to other short chain fatty acids [57]. With a robust assay capability on hundreds of different PM culture conditions, this assay technology can enable simultaneously studies of toxin regulation in cells with different or same genetic backgrounds and at different temperatures. This kind of multi-dimensional analysis would greatly enrich our understanding of the bacterium, its metabolism, and environmental factors affecting regulation of toxin production.

Influences of genetic factors on toxin production, in some cases, seem very clear. For example, for some non-toxic strains examined by Hammond and Johnson, the pathogenicity locus (PaLoc) is absent and a very short fragment (127 bp) occupies the same chromosomal location [58]. Emergent in North America, Europe, and Asia since 2003 [11], [18], [19], [20], [21], [22], hypervirulent strains, represented and dominated by ribotype 027 or BI/NAP1/027, produce high levels of toxin A and toxin B, which is presumably due to their harboring a *tcdC* repressor gene mutated at several distinct sites (an 18-bp, a 36-bp, a 39-bp deletion, or a single base pair deletion at position 117 that causes a frameshift introducing a stop codon at position 196 [18], [19], [47], [59], [60], [61], [62], [63]). However, some conflicting evidence has shown that truncation of the repressor gene *tcdC* does not cause higher toxin production in ribotype 027-related strains [64]. In addition, other groups have reported a lack of association of *tcdC* mutation type with disease severity in toxigenic *C. difficile* [65], [66], [67], [68], [69]. Therefore, regulation by genetic factors may be more complicated than expected. A comparable and direct functional or phenotypic toxin assay, such as the assay that we have demonstrated, should be helpful in sorting this out.

Spo0A, the master regulator for sporulation initiation, positively regulates toxin production [70]; and SigH, the key phase transcriptional factor, negatively regulates the toxin expression [71]. CodY, a global regulator of gene expression, directly binds to the promoter of *tcdR* with high affinity, down-regulating toxin genes *tcdA* and *tcdB* [72]. This binding is enhanced by GTP and branched-chain amino acids (leucine, isoleucine, and valine). Therefore, CodY may integrate toxin production with the nutrient status of *C. difficile*. [72], [73]. In our studies with the type strain of *C. difficile*, ATCC 9689, we found that toxin B production was most strongly repressed by some dipeptides containing leucine and the triple-leucine tripeptide as nitrogen sources in the presence of glucose. This appears to agree with the observation of leucine's enhancement of down-regulation via CodY. Leucine peptides may be taken up more efficienty than leucine. It would be very interesting to quantitatively measure functional toxin production with isogenic mutants of relevant regulatory genes under hundreds of nutritional culture conditions. This could provide new insights into understanding the coordination of nutrient metabolism and toxin production by these regulatory genes. Furthermore, if the leucine peptides are broadly and rapidly active in suppressing toxin production, they could perhaps be utilized to quell *C. difficile* induced toxicity in patients.

Over the past decades, there have been some prior studies of the effects of nutritional factors on *C. difficile* toxin production. It is well known that biotin deficiency increases toxin A and toxin B production significantly in some strains [74], [75] whereas glucose has been shown to reduce toxin production [76], [77], [78], [79]. In our studies, we used vitamin sufficient defined media by adding 0.5× of a vitamin mix (RPMI1640 vitamins, Sigma, used for mammalian cell culture). For biotin, the concentration used for *C. difficile* culture was 410 nM. Therefore, in these studies, biotin deficiency was not a factor. Looking at diverse carbon sources, we confirmed that glucose gives relatively low toxin production. Compared to N-Acetyl-D-Glucosamine (2657.19 ng/ml, PM1 A3), glucose gave a much lower level of toxin production (78.86 ng/ml, PM1 C9), only slightly higher than that of no substrate control of the panel (57.83 ng/ml, PM1 A1). The inhibition of *C. difficile* toxin production by glucose may be due to carbon catabolite repression through catabolite control protein A, CcpA [78]. On the other hand, glucose limitation reduced toxin production dramatically [74], [75], [77], which was also true in our observations when *C. difficile* was inoculated in PM3 with reduced glucose as carbon source (data not shown).

An influence of arginine on *C. difficile* toxin production has been noted previously, but the effect has not been consistent in reports: (1) toxin production increases with arginine addition in complex medium [80]; (2) toxin production increases with the absence or insufficiency of arginine [81]. In our assay, some arginine dipeptides increased toxin production dramatically (Figures 4A, Table 3), with less increase by arginine (458 ng/ml, Figure 4C, Table 3). This increase in toxin production by arginine or arginine dipeptides agrees with results of Osgood et al [80] and results from a plant pathogenic fungus using PM panels [5], where arginine and amines were strong toxin inducers in *Fusarium graminearum*. Culture conditions with nitrogen sources other than arginine or arginine dipeptides, can also produce high toxin levels, some of which are even higher (Figures 4A, 4C, Table 3, S2). This agrees with the results of Karasawa et al [81] who found that arginine is not absolutely required for increased toxin production. Not surprisingly, the regulation appears to be multifactorial. For example, arginine dipeptides containing branched-chain amino acids (Leu, Ile, and Val), gave much lower levels of toxin production than other arginine dipeptides, even lower than that of arginine itself. For example, *C. difficile* ATCC 9689 produced 282 ng/ml on Arg-Ile; 122 on Ile-Arg, 67 on Leu-Arg, 54 on Arg-Leu, 36 on Val-Arg, and 32 on Arg-Val (Table S2). These low toxin production levels could be explained, at least in part, by the branched-chain amino acids enhancing CodY's ability to down-regulate *tcdA* and *tcdB* [72]. Other interesting observations may have different underlying explanations. For example, while Arg-Ala and Lys-Arg gave very high toxin production, 10,080 and 5536 ng/ml, respectively (Figure 4A, Table 3), Ala-Arg gave a strikingly low level toxin production of 34 ng/ml (Table S2) and Arg-Lys gave a middle level of 308 ng/ml (Table S2). Presumably the specificity of toxin induction reflects differences in the transport and hydrolylsis of the specific peptides by *C. difficile*.

To our knowledge, this is the first report that nucleobase and nucleoside biochemicals are among the strongest *C. difficile* toxin inducers. In particular, adenine and guanosine top all substrates measured in this study, giving very high levels of toxin production (>168,000 ng/ml and 10402 ng/ml, respecitively) when they serve as nitrogen source in the presence of 5 mM glucose. In this nucleobase and nucleoside substrate group, many derivatives of purines and pyrimidines can stimulate high *C. difficile* toxin production as well (Figure 4A, Table 3). The reason is not known, but it is clearly not a random phenomenon. Maegawa et al [82] report that there is a linkage between toxin production and purine biosynthesis in *C. difficile*. Further investigation of these nucleobases and nucleosides inducing high levels of toxin may provide a new insight into the biochemical and molecular basis of *C. difficile* toxin regulation and may also help in explaining the clinical severity of CDI. Given induction of high toxin production by uric acid, a metabolite of purine, a question could be rationally asked: what is the correlation between circulating level of uric acid and severity or even mortality of CDI in patients?

The nutrients in the basal medium used in this study were present at low concentrations and systematically varied. Each well of panels PM3, 6, 7, and 8 contains these same components except one nitrogen source which differs from well to well (Materials & Methods, Table S1). So, the differences in toxin production are truly related to the specific difference of the nitrogen source among the wells. This is in contrast with previous studies where much richer and more complex media were typically used. For example, amino acids were added up to 35 mM plus 27.78 mM glucose and vitamins and minerals [83], or 39 mM amino acids plus 11.11 mM glucose and other nutrients [84], or 82 mM amino acids plus 11.11 mM glucose and other nutrients [74]. BHI plus 20.83 mM glucose (0.375%) or TY (3% bacto tryptose and 2% yeast extract) plus 55.5 mM glucose were used [72]; PT (peptone-yeast extract) plus 50 mM glucose (PT_0_G) or PT plus 10 mM cysteine (PY_10_) were reported [76]. In our study, the relatively low nutritional PM culture conditions may partially contribute to the high toxin production, which agrees well with the notion that the toxin production increases when the bacteria are under stresses including environmental and nutritional factors. In fact, nutrients in the colon are at low levels based on human feces analysis [85] and *C. difficile* must compete for nutrients against other flora [86], [87], [88]. According to Goldblith's analysis [85], vitamins account on the average for about 0.01% of the feces in human and biotin is about 0.133 mg in feces collected over a period of 24 h (∼544 nM, given 0.133 mg biotin collected and assuming 1000 g wet weight of feces over 24 h). Therefore, the levels of nutrients of our PM culture conditions seem more close to the natural nutrient levels in the colon.

It is reported that before antibiotic treatment the average toxin B concentration in CDI patients' stools was 58.29 pM (∼15.68 ng/ml). After antibiotic treatment the toxin level was 3.3 pM (∼0.89 ng/ml) [89]. Human colonic mucosae *ex vivo* testing showed damaging effects after 5 h of exposure to toxin B at a concentration about 0.2 nM (53.8 ng/ml) [90]. The TcdB_HV_ toxin can cause broad tissue damage to zebrafish embryos at concentrations about 1 nM (∼270 ng/ml) and is cytotoxic to CHO-k1 cells with TCD_50_ of 23.7 pM (∼6.4 ng/ml) [44]. In this study, the lowest concentration of *C. difficile* toxin B that can be reliably detected and quantified is as low as 0.45 pM (∼0.122 ng/ml). Therefore, our method could find applications in direct measurement of clinical samples from patients or animals to improve diagnosis, treatment, and epidemiologic studies of CDI.

*Clostridium difficile* strain VPI 10463 (ATCC 43255) is a high toxin producer [91] and is the toxinotype reference strain, classified as toxinotype 0 [92], [93]. Strain ATCC 9689 is the *C. difficile* type strain, sharing the same toxinotype with VPI 10463. Presumably the toxins produced by the two strains are similar. Indeed, the neutralizing antibody raised against toxin B protein from VPI 16403 (Gallus Immunotech) is highly effective in neutralizing toxins from both strains. In a comparison study, we examined toxin production in the bacterial supernatants of the two strains under identical PM culture conditions. From this we estimate that VPI 10463 produced ∼100 to ∼1000 times more toxin in quantity (data not shown). However, some of the highest toxin-producing substrates (e.g., adenine and guanosine) already stimulate very high toxin production by ATCC 9689 and a further induction of 100–1000 fold in VPI 10463 seems less likely. A more likely explanation is that the toxin B molecules produced by VPI 10463 are more potent than those of ATCC 9689.

The toxins A and B we purchased (Listlab) have been subjected to purification and lyophilization processes whereas the toxin produced in this study are crude toxin protein preparations that are just filtered to remove cells and then assayed quickly. Given the large size of toxins A and B (300 kDa and 270 kDa, respectively), it is possible that the purified proteins may suffer some damage or conformational changes by physical or chemical forces during the processes of purification and lyophilization. Crude toxin proteins tested immediately or within a very short period of time without freezing may be more intact and therefore may retain more functionality and potency. The intactness of the toxin molecules may be particularly important for toxin autoprocessing for the toxin to become cytotoxic. If the toxin molecules in a sample are less damaged than the standard purified toxins against which the sample toxin is quantified, it may lead to assigning an artificially high toxin concentration to the sample. The opposite may also be true. Our PM culture conditions usually produce high level (or high potency) toxin at very low *C. difficile* mass, suggesting that we are more likely to be overestimating the toxin level as our preparations may contain higher levels of intact toxin molecules.

To generalize this assay technology, we also tested some other species of clostridia and other bacterial genera that are known or potential producers of cytotoxins. Species tested in this study that demonstrated cytotoxicity include *C. perfringens*, *C. tetani*, *C. sordellii*, *Bacillus cereus*, *Escherichia coli* O157:H7, *Shigella dysenteriae*, and *Listeria monocytogenes*. Species tested that did not demonstrate cytotoxicity include *E. coli* BW30270, *S. sonnei*, *L. innocua*. Thus, our new cytotoxicity assay has been further validated with diverse bacterial species.

In addition to studying the regulation of toxin production in species and strains that are known producers, the sensitive and general nature of this approach will also enable the discovery of novel toxin-producing strains and novel toxins, especially toxins that are produced only under a very restricted set of culture conditions, as was the case with the *F. graminearum* toxin [6] which inspired these studies.

In summary, this is the first demonstration of simultaneous study of a large number of culture conditions influencing bacterial toxin production in diverse bacteria. Toxin production is a central issue in pathogenesis of *C. difficile* and other pathogenic microorganisms. Reliable, quantitative, sensitive, and robust assays for functional toxins are essential and critical in scientific research and clinical practice. The functional toxin assay method presented here is such an assay technology. It is based on the gold standard cell-based cytotoxicity assay and also takes advantage of PM technology. With hundreds to thousands different culture conditions, this toxin assay technology can provide a wealth of information and insights into the regulation of the toxin production and pathogenesis of toxingenic microorganisms. It may also find beneficial applications in clinical and epidemiological research.

## Materials and Methods

### Phenotype MicroArray (PM) panels, chemicals, toxins, anti-toxins, bacterial strains, and mammalian cells lines

PM panels, inoculating fluid IF-0a GN/GP Base (or IF-0a for short), Redox Dye Mix MB and inoculating fluid IF-M1 are from Biolog, Inc. (Hayward, CA, USA). Other chemicals were purchased from Sigma-Aldrich (St. Louis, MO, USA) unless specified otherwise. Yeast extract (YE) was from Oxoid (UK). *Clostridium difficile* toxin A and toxin B were from List Biological Laboratory (Listlab, Campbell, CA, USA). It is important to use antibody of high quality and antibody from some vendors was not satisfactory. We ultimately sourced anti-toxin A and anti-toxin B polyclonal chicken IgYs from Gallus Immunotech (Fergus, Ontario, Canada). *Clostridium difficile* strains: type strain ATCC 9689 and Toxinotype 0 strain VPI 10463 (ATCC 43255) were purchased from American Type Culture Collection (ATCC, Manassas, VA, USA). Some other bacteria obtained through commercial sources inlude: *C. perfringens* (ATCC 25763), *C. tetani* (ATCC 19406), *C. sordellii* (ATCC 9714), *Bacillus cereus* (ATCC 14579), *Escherichia coli* O157:H7 (ATCC 43894), *Shigella dysenteriae* (ATCC 11835), *S. sonnei* (ATCC 25931), *L. innocua* (ATCC 33090). Others were obtained as gifts, including *E. coli* BW30270 (from Barry Wanner, USA), *Listeria monocytogenes* strains P14 and P14-A (from Jose Vasquez-Boland, UK). Mammalian cell lines CHO-k1, Vero, HT-29, and A549 were also purchased from ATCC. Cell growth medium RPMI 1640, fetal bovine serum (FBS), Penicillin-Streptomycin, glutamine, and trypsin were purchased from Invitrogen. Glucose solution and RPMI 1640 vitamins stock solution were from Sigma-Aldrich. For preparation of bacterial culture supernatants we used 96-well filter plates (Pall, 0.2 uM pore size, PN 8015). For cell-based cytotoxicity assay and subsequent cellular dye reduction assay we used tissue culture treated 96-well plates (BD Falcon 353072).

### Work flow

**Figure 7:**
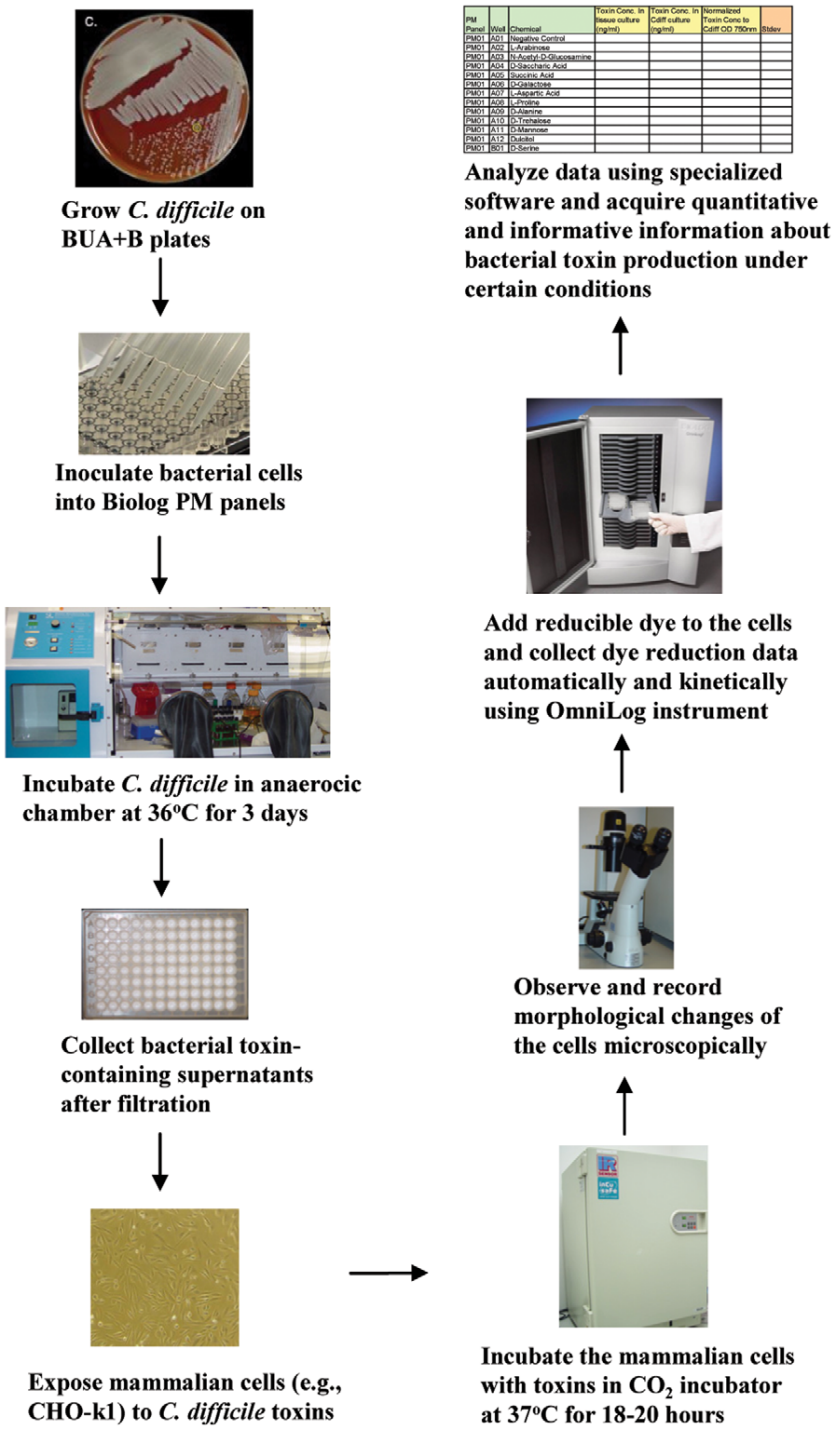
Flow chart of the process used in this study to quantitate toxin production by *C. difficile* under hundreds of culture conditions using PM technology.

Figure 7 depicts and summarizes the testing and assay for measuring *C. difficile* toxin production under different culture conditions in a 96-well panel format. This process is applied to all anaerobic bacteria. The incubation time for different bacteria may vary before toxins are collected. To collect toxins from aerobic bacteria, the process is the same as that for anaerobic bacteria except bacteria are incubated in an aerobic incubator instead of an anaerobic chamber.

### Bacterial preculture and inoculum preparation

*C. difficile* and other anaerobic bacterial strains were routinely pre-cultured on BUA+B agar (Biolog) inside an anaerobic chamber (Bactron IV,) at 36°C with a gas atmosphere of 5% H_2_, 5% CO_2_, and 90% N_2_. *C. difficile* fresh cultures (20–24 h) on BUA+B were used for PM panel inoculation. The inoculum of *C. difficile* was from fresh cultures in late log phase (Figure S2) which were examined and found to be free of endospores (Figure S3). Inocula were conveniently prepared by removing colonies from a BUA+B agar plate using a swab, and resuspending in IF-0a inoculating fluid. The bacterial suspension was adjusted in IF-0a to achieve a 40% transmittance (T40) using a Biolog Turbidimeter, which was measured spectrophotometrically as 0.139±0.002 O.D. (at 750 nm in a Multiskan Ascent). The suspension was further diluted 1∶16 in IF-0a. This cell density was directly used for PM panel inoculation. This inoculum (T40 1∶16) when plated on BUA+B gave a count of 9.07E+07 CFUs/ml.

### PM panel special pretreatment for anaerobic bacteria

Prior to their use with anaerobic bacteria, all PM panels were converted to an anaerobic state by thorough deoxygenation. To do this, the PM panel packaging bags are cut to open one end. Two oxygen absorbers (Ageless sachets, Mitsubishi) are inserted into the bag along with the original desiccate sachet and then the bag is resealed with a heat sealer. If the bag has a good seal, the sachets will absorb the air in the bag so that the packaging appears as if it is shrink-wrapped. This operation can be done on the lab bench. The resealed PM panels can be kept at room temperature for 1 day and then stored in the refrigerator (2–8°C) for additional days or months before use. The thoroughly deoxygenated panels can then be warmed up to room temperature before being used in an experiment. It is critical that the panels are resealed well, which allows the Ageless sachets to completely remove oxygen from the panels. It is not recommended to deoxygenate the PM panels by putting them in the anaerobic chamber because the moisture inside the chamber may destabilize some substrates. To deoxygenate Biolog IF-0a GN/GP Base, the bottle caps were loosened and the bottles were placed inside the anaerobic chamber for 3 days before use.

### PM media and bacterial toxin collection

PM panels are 96-well microplates containing a different substrate in each well. PM1 and PM2 are carbon source panels. PM3, 6, 7, and 8 are nitrogen source panels. PM4 contains various phosphate and sulfur sources; PM5 contains various biosynthesis pathway endproducts and nutrient supplements. The substrates in the PM wells are shown in Table S1. In addition to a unique substrate, each well of these metabolic panels also contains the needed minimal medium components [2] but without ferric chloride, tetrazolium violet, and sodium pyruvate added.

To produce and collect bacterial culture supernatants containing toxins, *C. difficile* or other bacterial species were inoculated in duplicate or triplicate into panels PM1–8 at T40 1∶16 and incubated for an optimum length of time, e.g., 3 days for *C. difficile*. The inoculating fluid for *C. difficile* consists of Biolog IF-0a GN/GP Base, 0.5× RPMI 1640 vitamins, and 0.2% yeast extract (sterilized by filtration) for PM1, 2 or 0.05% yeast extract for PM3, 4, 5, 6, 7, and 8. To provide the carbon source in PM3–8, glucose at a final concentration of 5 mM was added to the inoculating fluid. To obtain a crude toxin preparation free of cells, the bacterial liquid cultures were transferred to a 96-well filter plate (Pall) and filtered by centrifugation at 2000 rpm for 5 minutes (Hermle Z 360 K, rotor model C-0360-50). The filtrates were used immediately (the same day) or kept in a sterile 96-well plate (Biolog) sealed with tape for brief storage at 4°C before use. No bacteria controls (i.e. no toxin controls, or PM substrate controls) were PM substrate solutions rehydrated from the corresponding PM panels, obtained by following the same procedure for bacterial supernatants described above except no bacteria inoculated into the PM panels. All experiments described above were done multiple times (2–4) as independent replicates (2–3) and each was followed by cytotoxicity assays on the bacterial filtrates collected.

All aerobic bacteria tested in this study were handled on the bench and grown on Biolog agar medium (BUG+B) as a preculture and then inoculated and incubated aerobically in PM panels without shaking. After 24 h of incubation in the panels at 37°C, the bacterial supernatants were collected using the 96-well filter plates and centrifugation, and then handled and tested as described above.

The toxin-containing supernatants should be used as soon as possible after harvest because refrigeration temperature does not completely maintain the toxins during long term storage and the potency of the toxins was observed to decrease over time. Better long term storage of the toxins requires purification and lyophilization. These same procedures were followed for preparing PM substrate suspension controls without *C. difficile*.

### Determination of bacterial mass in wells of PM panels

To deteremine bacterial mass kinetics, PM panels inoculated with *C. difficile* in triplicate were removed from the anaerobic chamber at 24, 48, or 72 h of incubation. Bacterial mass was determined by measuring the optical density of wells at 750 nm using a microplate reader (Multiskan Ascent). Compared to 48 h or 72 h, the values of OD-750 at 24 h of incubation were the highest under most PM well conditions. With a few exceptions, all the OD-750 values decreased more or less at 48 h and further decreased at 72 h (Figure S1). Given the nutritional limitations of the media, the growth was limited, but the kinetic trends were clear and similar to each other among the various culture conditions (Figure S1).

To determine the bacterial mass at the end point and before harvesting toxin-containing supernatants, the PM panels with *C. difficile* in replicates were removed from the anaerobic chamber at 72 h and the mass was deteremined as decribed above. To obtain net bacterial mass, the OD-750 value from the corresponding well of an uninoculated plate is subtracted from the OD-750 value of the inoculated well. These bacterial net mass values can be used to normalize toxin production when a specific cell productivity measurement is needed.

### Mammalian cell lines and their cytotoxicity and neutralization assays

Cell lines CHO-k1, Vero, HT-29, and A549 were grown at 37°C in an incubator atmosphere of 5% CO_2_ in standard T75 cell culture flasks with RPMI 1640 medium plus 10% FBS and 1× Pen/Strep, without phenol red. The cells were allowed to grow for 20–24 h before harvesting for experiments. This young cell culture was then prepared in an assay medium (Biolog IF-M1 inoculating fluid plus 2 mM glucose, 2 mM glutamine, 2% FBS, and 1× Pen/Strep) at a density of 200,000 cells per ml. A 100 µl aliquot of this cell suspension was plated into each well of tissue culture treated 96-well microplates, the assay plate. Five microliters of toxin preparation either from serial titrations of purified standard toxin (Listlab) or from supernatants of microbial culture filtrates collected from each PM culture condition were transferred to each well of the assay plate immediately following the cell plating. This makes the toxin preparation a 21-fold dilution [(5+100)/5]. Five µl of corresponding PM substrate solution were used as no toxin control. The assay plates of the treated indicator cells were then incubated at 37°C with 5% CO_2_ for 18–20 h.

For neutralization experiments, a neutralizing antibody was mixed with the indicator cell suspension immediately prior to cell plating. The cell plating was followed immediately by addition of the toxin preparation to the plated cells. After 18–20 h of incubation, cell morphologies were observed under the microscope (10×10 magnification) and images were recorded with a digital camera. These same cells were further tested for their ability to reduce the Biolog Dye Mix MB. Twenty µl of the dye solution was transferred to each well. The plates were then placed into the OmniLog PM instrument (Biolog, Inc.) for incubation and kinetic data collection for 3 h or longer. As the dye is reduced, a purple color is irreversibly developed. The healthier the cells are, the more NADH they produce and the higher the rate of dye reduction [4].

### Determination of dye reduction rate by the mammalian cells

The dye reduction by indicator cells was kinetically measured during the incubation period by the OmniLog instrument. The resulting dye reduction values were imported into a PM analysis program, which can calculate a rate of dye reduction based on a linear regression algorithm. The rate of dye reduction within the first few hours of incubation (e.g., 0 through 3 h) was calculated. The effects of standard *C. difficile* toxin B or *C. difficile* supernatants collected from different PM conditions on the rate were studied. The average rates of the standard toxin or of the supernatants were used in all subsequent calculations.

To correct for the influence (positive or negative) of a given PM substrate on the rate, the dye reduction rate of the PM substrate control is measured. A ratio of a PM substrate is derived from the control set and defined as a quotient of the average rate of all wells for a given PM panel and its replicas (e.g., an average of 3 PM1 panels is obtained from 3×96 wells) divided by the average of a given well in that panel. So, if a PM substrate has a positive influence on the dye reduction rate of the indicator cell line, the ratio will be smaller than 1, otherwise greater than 1. For example, D-glucose (Table S1, PM1 C9) could positively influence dye reduction because the substrate control experiments showed it was associated with higher dye reduction rate than the panel average (78.48 vs 74.93). So, the ratio for D-glucose is smaller than 1 (0.9547). The average dye reduction rate of CHO-k1 in the presence of *C. difficile* supernatant from D-glucose is 57.19, but the corrected dye reduction rate is 57.19×0.9547 = 54.6.

In general, the rate of dye reduction by cells can be affected by many factors, e.g., cell lines, cell number and fitness, adverse environmental conditions (e.g., toxic chemicals), nutrient or energy source used to support cell respiration, the type of redox dyes, and so on. In these experiments, however, all factors and conditions given are controlled except the levels of toxins produced by the microorganism under the different culture conditions in the PM panels.

As mentioned above, all experiments were done multiple times and as independent replicates. From these, the averages and standard deviations of dye reduction rates were calculated, which are expressed as a mean +/− standard deviation and ploted as histograms exemplified by Figrure 3D. Statistical analyses were perfomed using Microsoft Office Excel TTEST via Excel automation on the rates between the experimental set and the control set on a well-by-well basis. The data were treated as two-tailed distributions with unequal variance. The difference is considered significant only if the probability of no difference between the means of the two sets is smaller than 5%, P<0.05.

Having measured the effect of the toxin as a dye reduction rate, the next step is to convert the rate into a concentration value for the toxin.

### Determination of toxin production by *C. difficile* and other Clostridium species

Anaerobic bacteria used in this study including *C. difficile*, *C. perfringens*, *C. tetani*, and *C. sordellii*, were incubated for three days before removal from the anaerobic chamber for cell mass measurement and toxin collection. (1) Establishment of standard curves of toxin and dye reduction rate. *C. difficile* toxins A and B from Listlab were used as standards to determine the relationship between toxin concentration and the corresponding level of inhibition of dye reduction rate by the indicator mammalian cells. Cytotoxicity and dye reduction assays with serial 2-fold or 3-fold standard toxin titrations were performed multiple times. For a given indicator cell line, the known concentrations of the toxin (ng/ml) used in the assay were plotted on the Y-axis with a log_10_ scale against corresponding dye reduction rates plotted on the X-axis (Figure S4). Using Microsoft Office Excel 2002 software, the plots were fitted to curves by non-linear regression analyses and the equations for predicting concentrations from the rates were generated (Figure S4, Table 1). (2) Determination of toxin production by *C. difficile* under different PM culture conditions. The corrected average dye reduction rates from the *C. difficile* supernatants were used to calculate the toxin concentration according to the equations (Figure S4, Table 1). The calculations were performed robustly by employing Excel worksheet functions.

## Supporting Information

Figure S1 ***C. difficile*** **mass kinetics in wells of PM panels.** The bacterial mass was determined at 24, 48, and 72 h of incubation as described in Materials and Methods. A, PM1. B, PM3.
(TIF)

Figure S2 **Growth curve of** ***C. difficile*** **colonies on BUA+B.** Single colonies of *C. difficile* (ATCC 9689) grown on BUA+B were picked up and cell counts were made at the time points, 18, 24, 48, and 72 h. As the colony size was very small at 18 h, 4 colonies were used instead of one, but the count of colony forming units (CFUs) was normalized to a single colony by dividing by 4. To count the viable cells, the colonies were suspended in IF-0a followed by serial dilution and plating on BUA+B. The number of viable cells was determined by counting CFUs.
(TIF)

Figure S3 **Endospore formation of** ***C. difficile*** **(ATCC 9689) at different ages on BUA+B.** At 24, 48, or 72 h of incubation on BUA+B, a cell suspension was made from a single colony in 1 ml IF-0a inoculating fluid. The cell suspension was then incubated in an eppendorf tube without or with 200 proof ethanol (cell suspension ∶ ethanol = 1∶1) for 1 h inside the anaerobic chamber. Then, 30 µl of each suspension was plated on a BUA+B plate. All plates were incubated in the chamber at 36°C for 24 h and then photographed. As shown, without ethanol, a bacterial lawn was formed from cultures of different ages on BUA+B (24, 48, and 72 h). Endospores appear as colonies that survive the ethanol treatment. Note that no endospores were formed from the 24 h culture but then the number of endospores increased as the culture age increased (72 h>48 h).
(TIF)

Figure S4 **Plots of dye reduction rate by CHO-k1 cells against corresponding standard** ***C. difficile*** **toxin B.** A serial 3-fold titrations of standard toxin B (Listlab) was performed on CHO-k1 cells. From top panel to bottom: The toxin concentrations decreased. Dye reduction kinetics were recorded by the OmniLog instrument and reduction rates (0–3 h) at different toxin concentrations were calculated using PM Analysis Software. The known standard toxin concentrations (Y-axis) were plotted against the corresponding Mean ± Stdev of the rates (X-axis). Regression analyses on the data were performed using Microsoft Excel 2002 software. Each equation given was suitable only for a certain range of toxin level as illustrated, and may be suitable only for the given conditions (cell line, assay conditions, type of reducible dye, etc.) as described in the text.
(TIF)

Table S1 Plate maps of Phenotype MicroArrays microplate panels PM1 through PM8.
(PDF)

Table S2 Toxin production of *C. difficile* ATCC 9689 under different PM conditions.
(PDF)
