# Supplementary figures and images for: Using Phenotype MicroArrays to Determine Culture Conditions That Induce or Repress Toxin Production by Clostridium difficile and Other Microorganisms
Source: PLoS One. 2013 Feb 20;8(2):e56545. doi: 10.1371/journal.pone.0056545 (PMC3577869; doi:10.1371/journal.pone.0056545)

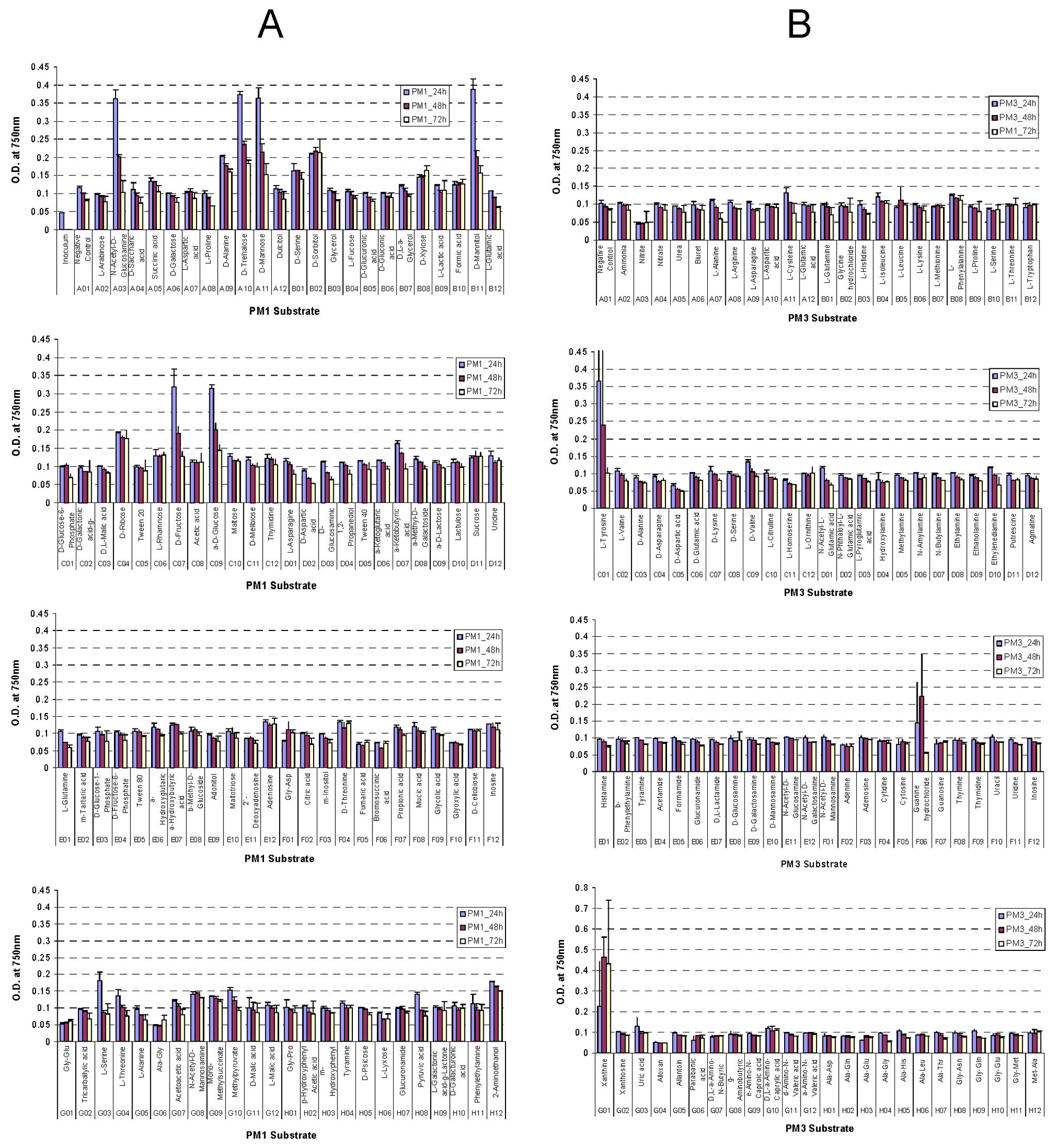

Supplement: Figure S1 — C. difficile mass kinetics in wells of PM panels. The bacterial mass was determined at 24, 48, and 72 h of incubation as described in Materials and Methods. A, PM1. B, PM3. (TIF) [file pone.0056545.s001.tif]

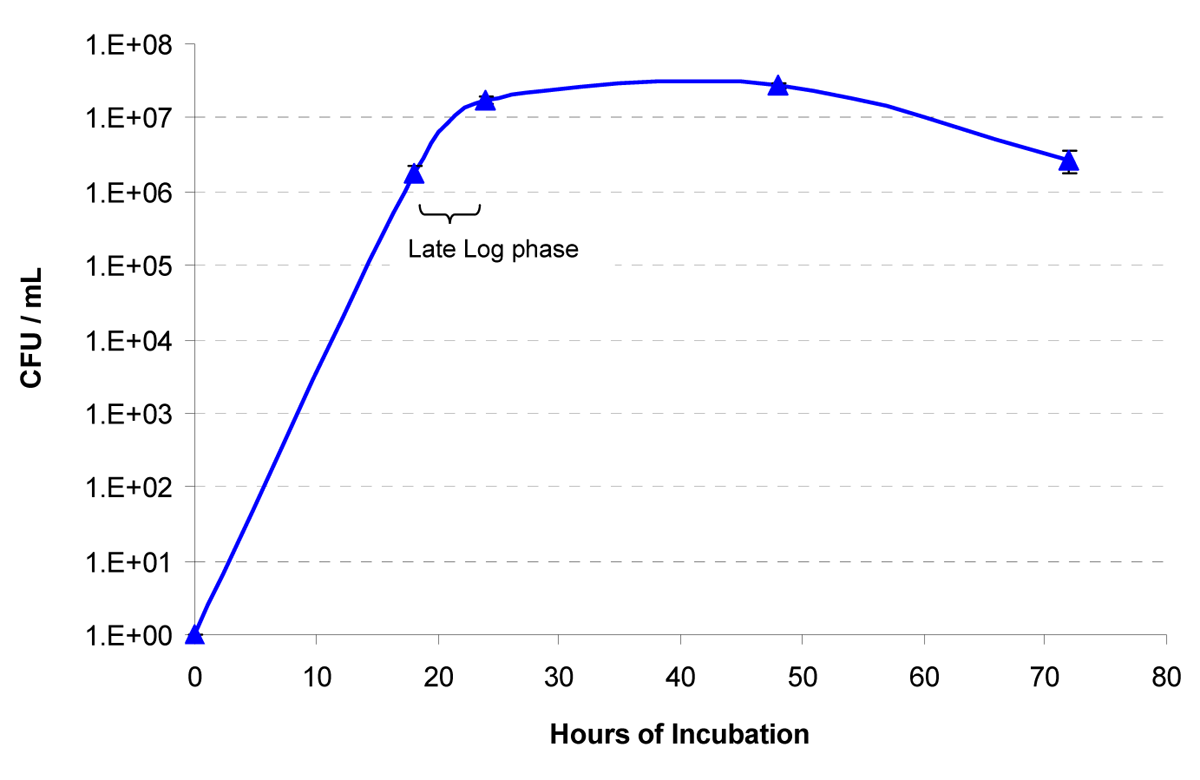

Supplement: Figure S2 — Growth curve of C. difficile colonies on BUA+B. Single colonies of C. difficile (ATCC 9689) grown on BUA+B were picked up and cell counts were made at the time points, 18, 24, 48, and 72 h. As the colony size was very small at 18 h, 4 colonies were used instead of one, but the count of colony forming units (CFUs) was normalized to a single colony by dividing by 4. To count the viable cells, the colonies were suspended in IF-0a followed by serial dilution and plating on BUA+B. The number of viable cells was determined by counting CFUs. (TIF) [file pone.0056545.s002.tif]

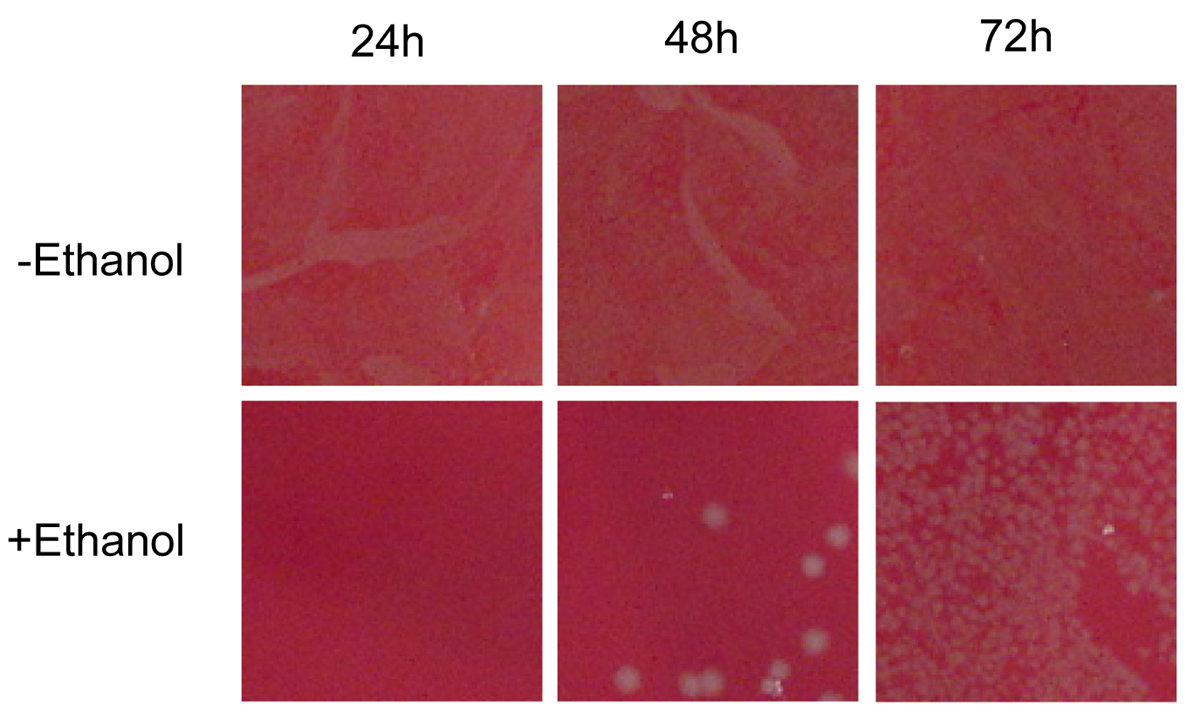

Supplement: Figure S3 — Endospore formation of C. difficile (ATCC 9689) at different ages on BUA+B. At 24, 48, or 72 h of incubation on BUA+B, a cell suspension was made from a single colony in 1 ml IF-0a inoculating fluid. The cell suspension was then incubated in an eppendorf tube without or with 200 proof ethanol (cell suspension ∶ ethanol = 1∶1) for 1 h inside the anaerobic chamber. Then, 30 µl of each suspension was plated on a BUA+B plate. All plates were incubated in the chamber at 36°C for 24 h and then photographed. As shown, without ethanol, a bacterial lawn was formed from cultures of different ages on BUA+B (24, 48, and 72 h). Endospores appear as colonies that survive the ethanol treatment. Note that no endospores were formed from the 24 h culture but then the number of endospores increased as the culture age increased (72 h>48 h). (TIF) [file pone.0056545.s003.tif]

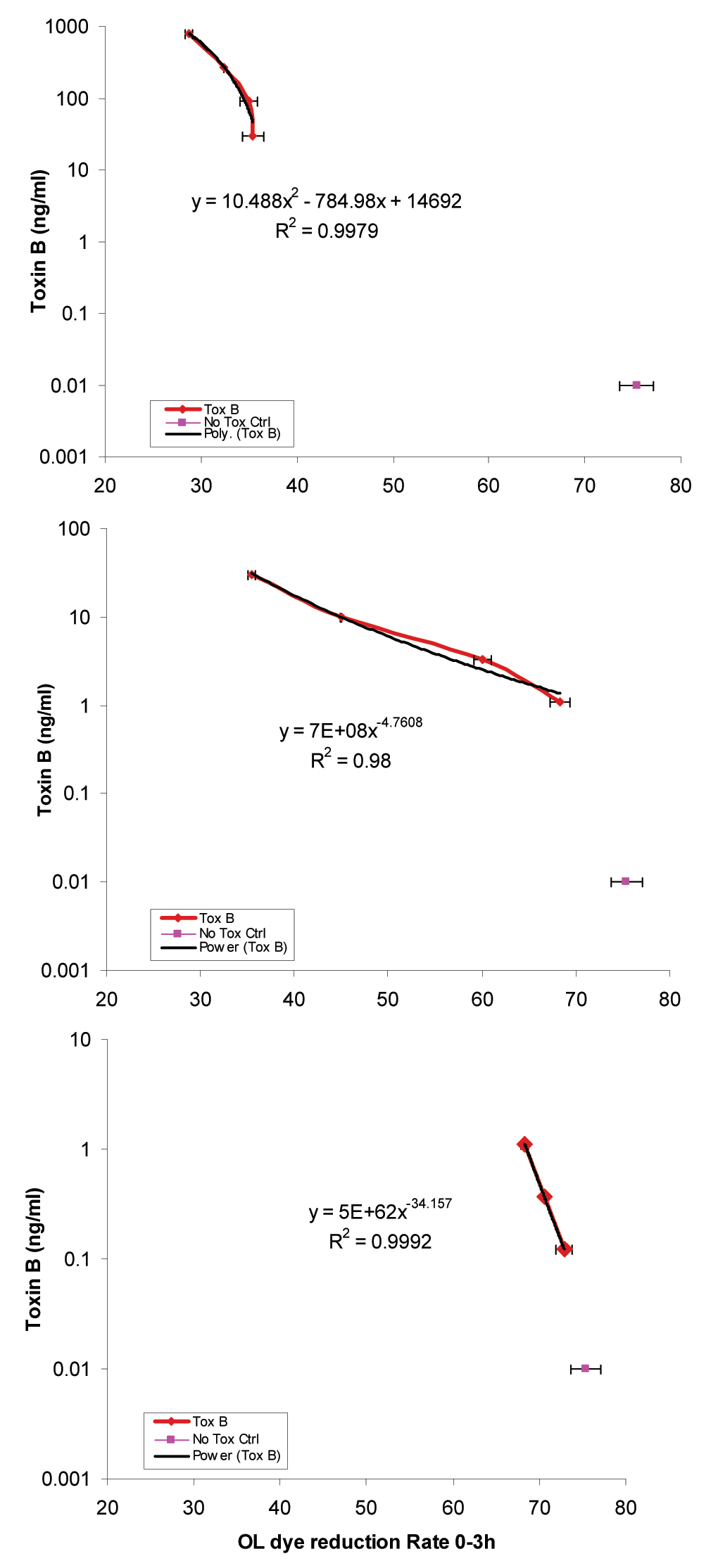

Supplement: Figure S4 — Plots of dye reduction rate by CHO-k1 cells against corresponding standard C. difficile toxin B. A serial 3-fold titrations of standard toxin B (Listlab) was performed on CHO-k1 cells. From top panel to bottom: The toxin concentrations decreased. Dye reduction kinetics were recorded by the OmniLog instrument and reduction rates (0–3 h) at different toxin concentrations were calculated using PM Analysis Software. The known standard toxin concentrations (Y-axis) were plotted against the corresponding Mean ± Stdev of the rates (X-axis). Regression analyses on the data were performed using Microsoft Excel 2002 software. Each equation given was suitable only for a certain range of toxin level as illustrated, and may be suitable only for the given conditions (cell line, assay conditions, type of reducible dye, etc.) as described in the text. (TIF) [file pone.0056545.s004.tif]
