# Supplementary material for: Using Phenotype MicroArrays to Determine Culture Conditions That Induce or Repress Toxin Production by Clostridium difficile and Other Microorganisms
Source: PLoS One. 2013 Feb 20;8(2):e56545. doi: 10.1371/journal.pone.0056545 (PMC3577869; doi:10.1371/journal.pone.0056545)
Supplement: Table S1 — Plate maps of Phenotype MicroArrays microplate panels PM1 through PM8. (PDF) [file pone.0056545.s005.pdf]

BIOLOG

Phenotype MicroArrays™

PM1 MicroPlate™ Carbon Sources

A1 Negative Control	A2 L-Arabinose	A3 N-Acetyl-D-Glucosamine	A4 D-Saccharic Acid	A5 Succinic Acid	A6 D-Galactose	A7 L-Aspartic Acid	A8 L-Proline	A9 D-Alanine	A10 D-Trehalose	A11 D-Mannose	A12 Dulcitol
B1 D-Serine	B2 D-Sorbitol	B3 Glycerol	B4 L-Fucose	B5 D-Glucuronic Acid	B6 D-Gluconic Acid	B7 D,L-α-Glycerol-Phosphate	B8 D-Xylose	B9 L-Lactic Acid	B10 Formic Acid	B11 D-Mannitol	B12 L-Glutamic Acid
C1 D-Glucose-6-Phosphate	C2 D-Galactonic Acid-γ-Lactone	C3 D,L-Malic Acid	C4 D-Ribose	C5 Tween 20	C6 L-Rhamnose	C7 D-Fructose	C8 Acetic Acid	C9 α-D-Glucose	C10 Maltose	C11 D-Melibiose	C12 Thymidine
D-1 L-Asparagine	D2 D-Aspartic Acid	D3 D-Glucosaminic Acid	D4 1,2-Propanediol	D5 Tween 40	D6 α-Keto-Glutaric Acid	D7 α-Keto-Butyric Acid	D8 α-Methyl-D-Galactoside	D9 α-D-Lactose	D10 Lactulose	D11 Sucrose	D12 Uridine
E1 L-Glutamine	E2 M-Tartaric Acid	E3 D-Glucose-1-Phosphate	E4 D-Fructose-6-Phosphate	E5 Tween 80	E6 α-Hydroxy Glutaric Acid-γ-Lactone	E7 α-Hydroxy Butyric Acid	E8 β-Methyl-D-Glucoside	E9 Adonitol	E10 Maltotriose	E11 2-Deoxy Adenosine	E12 Adenosine
F1 Glycyl-L-Aspartic Acid	F2 Citric Acid	F3 M-Inositol	F4 D-Threonine	F5 Fumaric Acid	F6 Bromo Succinic Acid	F7 Propionic Acid	F8 Mucic Acid	F9 Glycolic Acid	F10 Glyoxylic Acid	F11 D-Cellobiose	F12 Inosine
G1 Glycyl-L-Glutamic Acid	G2 Tricarballylic Acid	G3 L-Serine	G4 L-Threonine	G5 L-Alanine	G6 L-Alanyl-Glycine	G7 Acetoacetic Acid	G8 N-Acetyl-β-D-Mannosamine	G9 Mono Methyl Succinate	G10 Methyl Pyruvate	G11 D-Malic Acid	G12 L-Malic Acid
H1 Glycyl-L-Proline	H2 p-Hydroxy Phenyl Acetic Acid	H3 m-Hydroxy Phenyl Acetic Acid	H4 Tyramine	H5 D-Psicose	H6 L-Lyxose	H7 Glucuronamide	H8 Pyruvic Acid	H9 L-Galactonic Acid-γ-Lactone	H10 D-Galacturonic Acid	H11 Phenylethyl-amine	H12 2-Aminoethanol

PM2A MicroPlate™ Carbon Sources

A1 Negative Control	A2 Chondroitin Sulfate C	A3 α-Cyclodextrin	A4 β-Cyclodextrin	A5 γ-Cyclodextrin	A6 Dextrin	A7 Gelatin	A8 Glycogen	A9 Inulin	A10 Laminarin	A11 Mannan	A12 Pectin
B1 N-Acetyl-D-Galactosamine	B2 N-Acetyl-Neuraminic Acid	B3 β-D-Allose	B4 Amygdalin	B5 D-Arabinose	B6 D-Arabitol	B7 L-Arabitol	B8 Arbutin	B9 2-Deoxy-D-Ribose	B10 I-Erythritol	B11 D-Fucose	B12 3-0-β-D-Galacto-pyranosyl-D-Arabinose
C1 Gentiobiose	C2 L-Glucose	C3 Lactitol	C4 D-Melezitose	C5 Maltitol	C6 α-Methyl-D-Glucoside	C7 β-Methyl-D-Galactoside	C8 3-Methyl Glucose	C9 β-Methyl-D-Glucuronic Acid	C10 α-Methyl-D-Mannoside	C11 β-Methyl-D-Xyloside	C12 Palatinose
D1 D-Raffinose	D2 Salicin	D3 Sedoheptulosan	D4 L-Sorbose	D5 Stachyose	D6 D-Tagatose	D7 Turanose	D8 Xylitol	D9 N-Acetyl-D-Glucosaminitol	D10 γ-Amino Butyric Acid	D11 δ-Amino Valeric Acid	D12 Butyric Acid
E1 Capric Acid	E2 Caproic Acid	E3 Citraconic Acid	E4 Citramalic Acid	E5 D-Glucosamine	E6 2-Hydroxy Benzoic Acid	E7 4-Hydroxy Benzoic Acid	E8 β-Hydroxy Butyric Acid	E9 γ-Hydroxy Butyric Acid	E10 α-Keto Valeric Acid	E11 Itaconic Acid	E12 5-Keto-D-Gluconic Acid
F1 D-Lactic Acid Methyl Ester	F2 Malonic Acid	F3 Melibionic Acid	F4 Oxalic Acid	F5 Oxalomalic Acid	F6 Quinic Acid	F7 D-Ribono-1,4-Lactone	F8 Sebacic Acid	F9 Sorbic Acid	F10 Succinamic Acid	F11 D-Tartaric Acid	F12 L-Tartaric Acid
G1 Acetamide	G2 L-Alaninamide	G3 N-Acetyl-L-Glutamic Acid	G4 L-Arginine	G5 Glycine	G6 L-Histidine	G7 L-Homoserine	G8 Hydroxy-L-Proline	G9 L-Isoleucine	G10 L-Leucine	G11 L-Lysine	G12 L-Methionine
H1 L-Ornithine	H2 L-Phenylalanine	H3 L-Pyroglutamic Acid	H4 L-Valine	H5 D,L-Carnitine	H6 Sec-Butylamine	H7 D.L-Octopamine	H8 Putrescine	H9 Dihydroxy Acetone	H10 2,3-Butanediol	H11 2,3-Butanone	H12 3-Hydroxy 2-Butanone

Phenotype MicroArrays™

PM3B MicroPlate™ Nitrogen Sources

A1 Negative Control	A2 Ammonia	A3 Nitrite	A4 Nitrate	A5 Urea	A6 Biuret	A7 L-Alanine	A8 L-Arginine	A9 L-Asparagine	A10 L-Aspartic Acid	A11 L-Cysteine	A12 L-Glutamic Acid
B1 L-Glutamine	B2 Glycine	B3 L-Histidine	B4 L-Isoleucine	B5 L-Leucine	B6 L-Lysine	B7 L-Methionine	B8 L-Phenylalanine	B9 L-Proline	B10 L-Serine	B11 L-Threonine	B12 L-Tryptophan
C1 L-Tyrosine	C2 L-Valine	C3 D-Alanine	C4 D-Asparagine	C5 D-Aspartic Acid	C6 D-Glutamic Acid	C7 D-Lysine	C8 D-Serine	C9 D-Valine	C10 L-Citrulline	C11 L-Homoserine	C12 L-Ornithine
D-1 N-Acetyl-D,L-Glutamic Acid	D2 N-Phthaloyl-L-Glutamic Acid	D3 L-Pyroglutamic Acid	D4 Hydroxylamine	D5 Methylamine	D6 N-Amylamine	D7 N-Butylamine	D8 Ethylamine	D9 Ethanolamine	D10 Ethylenediamine	D11 Putrescine	D12 Agmatine
E1 Histamine	E2 β-Phenylethyl-amine	E3 Tyramine	E4 Acetamide	E5 Formamide	E6 Glucuronamide	E7 D,L-Lactamide	E8 D-Glucosamine	E9 D-Galactosamine	E10 D-Mannosamine	E11 N-Acetyl-D-Glucosamine	E12 N-Acetyl-D-Galactosamine
F1 N-Acetyl-D-Mannosamine	F2 Adenine	F3 Adenosine	F4 Cytidine	F5 Cytosine	F6 Guanine	F7 Guanosine	F8 Thymine	F9 Thymidine	F10 Uracil	F11 Uridine	F12 Inosine
G1 Xanthine	G2 Xanthosine	G3 Uric Acid	G4 Alloxan	G5 Allantoin	G6 Parabanic Acid	G7 D,L-α-Amino-N-Butyric Acid	G8 γ-Amino-N-Butyric Acid	G9 ε-Amino-N-Caproic Acid	G10 D,L-α-Amino-Caprylic Acid	G11 δ-Amino-N-Valeric Acid	G12 α-Amino-N-Valeric Acid
H1 Ala-Asp	H2 Ala-Gln	H3 Ala-Glu	H4 Ala-Gly	H5 Ala-His	H6 Ala-Leu	H7 Ala-Thr	H8 Gly-Asn	H9 Gly-Gln	H10 Gly-Glu	H11 Gly-Met	H12 Met-Ala

PM4A MicroPlate™ Phosphorus and Sulfur Sources

A1 Negative Control	A2 Phosphate	A3 Pyrophosphate	A4 Trimeta-phosphate	A5 Tripoly-phosphate	A6 Triethyl Phosphate	A7 Hypophosphite	A8 Adenosine- 2'-monophosphate	A9 Adenosine- 3'-monophosphate	A10 Adenosine- 5'-monophosphate	A11 Adenosine-2',3'-cyclic monophosphate	A12 Adenosine-3',5'-cyclic monophosphate
B1 Thiophosphate	B2 Dithiophosphate	B3 D,L-α-Glycerol Phosphate	B4 β-Glycerol Phosphate	B5 Carbamyl Phosphate	B6 D-2-Phospho-Glyceric Acid	B7 D-3-Phospho-Glyceric Acid	B8 Guanosine- 2'-monophosphate	B9 Guanosine- 3'-monophosphate	B10 Guanosine- 5'-monophosphate	B11 Guanosine-2',3'-cyclic monophosphate	B12 Guanosine-3',5'-cyclic monophosphate
C1 Phosphoenol Pyruvate	C2 Phospho-Glycolic Acid	C3 D-Glucose-1-Phosphate	C4 D-Glucose-6-Phosphate	C5 2-Deoxy-D-Glucose 6-Phosphate	C6 D-Glucosamine-6-Phosphate	C7 6-Phospho-Gluconic Acid	C8 Cytidine- 2'-monophosphate	C9 Cytidine- 3'-monophosphate	C10 Cytidine- 5'-monophosphate	C11 Cytidine- 2',3'-cyclic monophosphate	C12 Cytidine- 3',5'-cyclic monophosphate
D1 D-Mannose-1-Phosphate	D2 D-Mannose-6-Phosphate	D3 Cysteamine-S-Phosphate	D4 Phospho-L-Arginine	D5 O-Phospho-D-Serine	D6 O-Phospho-L-Serine	D7 O-Phospho-L-Threonine	D8 Uridine- 2'-monophosphate	D9 Uridine- 3'-monophosphate	D10 Uridine- 5'-monophosphate	D11 Uridine- 2',3'-cyclic monophosphate	D12 Uridine- 3',5'-cyclic monophosphate
E1 O-Phospho-D-Tyrosine	E2 O-Phospho-L-Tyrosine	E3 Phosphocreatine	E4 Phosphoryl Choline	E5 O-Phosphoryl-Ethanolamine	E6 Phosphono Acetic Acid	E7 2-Aminoethyl Phosphonic Acid	E8 Methylene Diphosphonic Acid	E9 Thymidine- 3'-monophosphate	E10 Thymidine- 5'-monophosphate	E11 Inositol Hexaphosphate	E12 Thymidine 3',5'- cyclic monophosphate
F1 Negative Control	F2 Sulfate	F3 Thiosulfate	F4 Tetrathionate	F5 Thiophosphate	F6 Dithiophosphate	F7 L-Cysteine	F8 D-Cysteine	F9 L-Cysteinyl-Glycine	F10 L-Cysteic Acid	F11 Cysteamine	F12 L-Cysteine Sulfinic Acid
G1 N-Acetyl-L-Cysteine	G2 S-Methyl-L-Cysteine	G3 Cystathionine	G4 Lanthionine	G5 Glutathione	G6 D,L-Ethionine	G7 L-Methionine	G8 D-Methionine	G9 Glycyl-L-Methionine	G10 N-Acetyl-D,L-Methionine	G11 L- Methionine Sulfoxide	G12 L-Methionine Sulfone
H1 L-Djenkolic Acid	H2 Thiourea	H3 1-Thio-β-D-Glucose	H4 D,L-Lipoamide	H5 Taurocholic Acid	H6 Taurine	H7 Hypotaurine	H8 p-Amino Benzene Sulfonic Acid	H9 Butane Sulfonic Acid	H10 2-Hydroxyethane Sulfonic Acid	H11 Methane Sulfonic Acid	H12 Tetramethylene Sulfone

BiOLOG

Phenotype MicroArrays™

PM5 MicroPlate™ Nutrient Supplements

A1<br>Negative Control	A2<br>Positive Control	A3<br>L-Alanine	A4<br>L-Arginine	A5<br>L-Asparagine	A6<br>L-Aspartic Acid	A7<br>L-Cysteine	A8<br>L-Glutamic Acid	A9<br>Adenosine-3',5'-cyclic monophosphate	A10<br>Adenine	A11<br>Adenosine	A12<br>2'-Deoxy Adenosine
B1<br>L-Glutamine	B2<br>Glycine	B3<br>L-Histidine	B4<br>L-Isoleucine	B5<br>L-Leucine	B6<br>L-Lysine	B7<br>L-Methionine	B8<br>L-Phenylalanine	B9<br>Guanosine-3',5'-cyclic monophosphate	B10<br>Guanine	B11<br>Guanosine	B12<br>2'-Deoxy Guanosine
C1<br>L-Proline	C2<br>L-Serine	C3<br>L-Threonine	C4<br>L-Tryptophan	C5<br>L-Tyrosine	C6<br>L-Valine	C7<br>L-Isoleucine + L-Valine	C8<br>trans-4-Hydroxy L-Proline	C9<br>(5) 4-Amino-Imidazole-4(5)-Carboxamide	C10<br>Hypoxanthine	C11<br>Inosine	C12<br>2'-Deoxy Inosine
D1<br>L-Ornithine	D2<br>L-Citrulline	D3<br>Chorismic Acid	D4<br>(-)Shikimic Acid	D5<br>L-Homoserine Lactone	D6<br>D-Alanine	D7<br>D-Aspartic Acid	D8<br>D-Glutamic Acid	D9<br>D,L-α,ε-Diamino-pimelic Acid	D10<br>Cytosine	D11<br>Cytidine	D12<br>2'-Deoxy Cytidine
E1<br>Putrescine	E2<br>Spermidine	E3<br>Spermine	E4<br>Pyridoxine	E5<br>Pyridoxal	E6<br>Pyridoxamine	E7<br>β-Alanine	E8<br>D-Pantothenic Acid	E9<br>Orotic Acid	E10<br>Uracil	E11<br>Uridine	E12<br>2'-Deoxy Uridine
F1<br>Quinolinic Acid	F2<br>Nicotinic Acid	F3<br>Nicotinamide	F4<br>β-Nicotinamide Adenine Dinucleotide	F5<br>δ-Amino-Levulinic Acid	F6<br>Hematin	F7<br>Deferoxamine Mesylate	F8<br>D-(+)-Glucose	F9<br>N-Acetyl D-Glucosamine	F10<br>Thymine	F11<br>Glutathione (reduced form)	F12<br>Thymidine
G1<br>Oxaloacetic Acid	G2<br>D-Biotin	G3<br>Cyano-Cobalamine	G4<br>p-Amino-Benzoic Acid	G5<br>Folic Acid	G6<br>Inosine + Thiamine	G7<br>Thiamine	G8<br>Thiamine Pyrophosphate	G9<br>Riboflavin	G10<br>Pyrrolo-Quinoline Quinone	G11<br>Menadione	G12<br>Myo-Inositol
H1<br>Butyric Acid	H2<br>D,L-α-Hydroxy-Butyric Acid	H3<br>α-Ketobutyric Acid	H4<br>Caprylic Acid	H5<br>D,L-α-Lipoic Acid (oxidized form)	H6<br>D,L-Mevalonic Acid	H7<br>D,L-Carnitine	H8<br>Choline	H9<br>Tween 20	H10<br>Tween 40	H11<br>Tween 60	H12<br>Tween 80

PM6 MicroPlate™ Peptide Nitrogen Sources

A1<br>Negative Control	A2<br>Positive Control: L-Glutamine	A3<br>Ala-Ala	A4<br>Ala-Arg	A5<br>Ala-Asn	A6<br>Ala-Glu	A7<br>Ala-Gly	A8<br>Ala-His	A9<br>Ala-Leu	A10<br>Ala-Lys	A11<br>Ala-Phe	A12<br>Ala-Pro
B1<br>Ala-Ser	B2<br>Ala-Thr	B3<br>Ala-Trp	B4<br>Ala-Tyr	B5<br>Arg-Ala	B6<br>Arg-Arg	B7<br>Arg-Asp	B8<br>Arg-Gln	B9<br>Arg-Glu	B10<br>Arg-Ile	B11<br>Arg-Leu	B12<br>Arg-Lys
C1<br>Arg-Met	C2<br>Arg-Phe	C3<br>Arg-Ser	C4<br>Arg-Trp	C5<br>Arg-Tyr	C6<br>Arg-Val	C7<br>Asn-Glu	C8<br>Asn-Val	C9<br>Asp-Asp	C10<br>Asp-Glu	C11<br>Asp-Leu	C12<br>Asp-Lys
D1<br>Asp-Phe	D2<br>Asp-Trp	D3<br>Asp-Val	D4<br>Cys-Gly	D5<br>Gln-Gln	D6<br>Gln-Gly	D7<br>Glu-Asp	D8<br>Glu-Glu	D9<br>Glu-Gly	D10<br>Glu-Ser	D11<br>Glu-Trp	D12<br>Glu-Tyr
E1<br>Glu-Val	E2<br>Gly-Ala	E3<br>Gly-Arg	E4<br>Gly-Cys	E5<br>Gly-Gly	E6<br>Gly-His	E7<br>Gly-Leu	E8<br>Gly-Lys	E9<br>Gly-Met	E10<br>Gly-Phe	E11<br>Gly-Pro	E12<br>Gly-Ser
F1<br>Gly-Thr	F2<br>Gly-Trp	F3<br>Gly-Tyr	F4<br>Gly-Val	F5<br>His-Asp	F6<br>His-Gly	F7<br>His-Leu	F8<br>His-Lys	F9<br>His-Met	F10<br>His-Pro	F11<br>His-Ser	F12<br>His-Trp
G1<br>His-Tyr	G2<br>His-Val	G3<br>Ile-Ala	G4<br>Ile-Arg	G5<br>Ile-Gln	G6<br>Ile-Gly	G7<br>Ile-His	G8<br>Ile-Ile	G9<br>Ile-Met	G10<br>Ile-Phe	G11<br>Ile-Pro	G12<br>Ile-Ser
H1<br>Ile-Trp	H2<br>Ile-Tyr	H3<br>Ile-Val	H4<br>Leu-Ala	H5<br>Leu-Arg	H6<br>Leu-Asp	H7<br>Leu-Glu	H8<br>Leu-Gly	H9<br>Leu-Ile	H10<br>Leu-Leu	H11<br>Leu-Met	H12<br>Leu-Phe

Phenotype MicroArrays™

PM7 MicroPlate™ Peptide Nitrogen Sources

A1 Negative Control	A2 Positive Control: L-Glutamine	A3 Leu-Ser	A4 Leu-Trp	A5 Leu-Val	A6 Lys-Ala	A7 Lys-Arg	A8 Lys-Glu	A9 Lys-Ile	A10 Lys-Leu	A11 Lys-Lys	A12 Lys-Phe
B1 Lys-Pro	B2 Lys-Ser	B3 Lys-Thr	B4 Lys-Trp	B5 Lys-Tyr	B6 Lys-Val	B7 Met-Arg	B8 Met-Asp	B9 Met-Gln	B10 Met-Glu	B11 Met-Gly	B12 Met-His
C1 Met-Ile	C2 Met-Leu	C3 Met-Lys	C4 Met-Met	C5 Met-Phe	C6 Met-Pro	C7 Met-Trp	C8 Met-Val	C9 Phe-Ala	C10 Phe-Gly	C11 Phe-Ile	C12 Phe-Phe
D1 Phe-Pro	D2 Phe-Ser	D3 Phe-Trp	D4 Pro-Ala	D5 Pro-Asp	D6 Pro-Gln	D7 Pro-Gly	D8 Pro-Hyp	D9 Pro-Leu	D10 Pro-Phe	D11 Pro-Pro	D12 Pro-Tyr
E1 Ser-Ala	E2 Ser-Gly	E3 Ser-His	E4 Ser-Leu	E5 Ser-Met	E6 Ser-Phe	E7 Ser-Pro	E8 Ser-Ser	E9 Ser-Tyr	E10 Ser-Val	E11 Thr-Ala	E12 Thr-Arg
F1 Thr-Glu	F2 Thr-Gly	F3 Thr-Leu	F4 Thr-Met	F5 Thr-Pro	F6 Trp-Ala	F7 Trp-Arg	F8 Trp-Asp	F9 Trp-Glu	F10 Trp-Gly	F11 Trp-Leu	F12 Trp-Lys
G1 Trp-Phe	G2 Trp-Ser	G3 Trp-Trp	G4 Trp-Tyr	G5 Tyr-Ala	G6 Tyr-Gln	G7 Tyr-Glu	G8 Tyr-Gly	G9 Tyr-His	G10 Tyr-Leu	G11 Tyr-Lys	G12 Tyr-Phe
H1 Tyr-Trp	H2 Tyr-Tyr	H3 Val-Arg	H4 Val-Asn	H5 Val-Asp	H6 Val-Gly	H7 Val-His	H8 Val-Ile	H9 Val-Leu	H10 Val-Tyr	H11 Val-Val	H12 γ-Glu-Gly

PM8 MicroPlate™ Peptide Nitrogen Sources

A1 Negative Control	A2 Positive Control: L-Glutamine	A3 Ala-Asp	A4 Ala-Gln	A5 Ala-Ile	A6 Ala-Met	A7 Ala-Val	A8 Asp-Ala	A9 Asp-Gln	A10 Asp-Gly	A11 Glu-Ala	A12 Gly-Asn
B1 Gly-Asp	B2 Gly-Ile	B3 His-Ala	B4 His-Glu	B5 His-His	B6 Ile-Asn	B7 Ile-Leu	B8 Leu-Asn	B9 Leu-His	B10 Leu-Pro	B11 Leu-Tyr	B12 Lys-Asp
C1 Lys-Gly	C2 Lys-Met	C3 Met-Thr	C4 Met-Tyr	C5 Phe-Asp	C6 Phe-Glu	C7 Gln-Glu	C8 Phe-Met	C9 Phe-Tyr	C10 Phe-Val	C11 Pro-Arg	C12 Pro-Asn
D1 Pro-Glu	D2 Pro-Ile	D3 Pro-Lys	D4 Pro-Ser	D5 Pro-Trp	D6 Pro-Val	D7 Ser-Asn	D8 Ser-Asp	D9 Ser-Gln	D10 Ser-Glu	D11 Thr-Asp	D12 Thr-Gln
E1 Thr-Phe	E2 Thr-Ser	E3 Trp-Val	E4 Tyr-Ile	E5 Tyr-Val	E6 Val-Ala	E7 Val-Gln	E8 Val-Glu	E9 Val-Lys	E10 Val-Met	E11 Val-Phe	E12 Val-Pro
F1 Val-Ser	F2 β-Ala-Ala	F3 β-Ala-Gly	F4 β-Ala-His	F5 Met-β-Ala	F6 β-Ala-Phe	F7 D-Ala-D-Ala	F8 D-Ala-Gly	F9 D-Ala-Leu	F10 D-Leu-D-Leu	F11 D-Leu-Gly	F12 D-Leu-Tyr
G1 γ-Glu-Gly	G2 γ-D-Glu-Gly	G3 Gly-D-Ala	G4 Gly-D-Asp	G5 Gly-D-Ser	G6 Gly-D-Thr	G7 Gly-D-Val	G8 Leu-β-Ala	G9 Leu-D-Leu	G10 Phe-β-Ala	G11 Ala-Ala-Ala	G12 D-Ala-Gly-Gly
H1 Gly-Gly-Ala	H2 Gly-Gly-D-Leu	H3 Gly-Gly-Gly	H4 Gly-Gly-Ile	H5 Gly-Gly-Leu	H6 Gly-Gly-Phe	H7 Val-Tyr-Val	H8 Gly-Phe-Phe	H9 Leu-Gly-Gly	H10 Leu-Leu-Leu	H11 Phe-Gly-Gly	H12 Tyr-Gly-Gly
