# Supplementary material for: Using Phenotype MicroArrays to Determine Culture Conditions That Induce or Repress Toxin Production by Clostridium difficile and Other Microorganisms
Source: PLoS One. 2013 Feb 20;8(2):e56545. doi: 10.1371/journal.pone.0056545 (PMC3577869; doi:10.1371/journal.pone.0056545)
Supplement: Table S2 — Toxin production of C. difficile ATCC 9689 under different PM conditions. (PDF) [file pone.0056545.s006.pdf]

Table S2. Toxin production of *C. difficile* ATCC 9689 under different PM conditions

PM Panel	Well	Chemical	*C. difficile* Mass[a]	Toxin Concentration (ng/ml)[b]	Normalized Dye Reduction Rate by PM Substrate[c]	P value[d]
PM01	A01	Negative Control	0.068	57.83	58.28	1.74E-02
PM01	A02	L-Arabinose	0.074	43.56	61.85	6.09E-02
PM01	A03	N-Acetyl-D-Glucosamine	0.095	2657.19	34.00	5.17E-05
PM01	A04	D-Saccharic Acid	0.074	39.88	63.01	1.74E-02
PM01	A05	Succinic Acid	0.077	39.98	62.97	6.72E-03
PM01	A06	D-Galactose	0.073	48.15	60.56	9.62E-05
PM01	A07	L-Aspartic Acid	0.074	40.41	62.83	1.62E-02
PM01	A08	L-Proline	0.046	41.29	62.55	1.33E-03
PM01	A09	D-Alanine	0.123	118.29	50.14	9.72E-06
PM01	A10	D-Trehalose	0.168	50.01	60.08	7.26E-04
PM01	A11	D-Mannose	0.162	67.07	56.49	1.02E-06
PM01	A12	Dulcitol	0.078	49.72	60.16	1.57E-02
PM01	B01	D-Serine	0.111	587.53	35.81	1.19E-03
PM01	B02	D-Sorbitol	0.150	53.68	59.19	3.11E-03
PM01	B03	Glycerol	0.070	36.52	64.18	8.44E-03
PM01	B04	L-Fucose	0.071	37.53	63.82	8.00E-04
PM01	B05	D-Glucuronic Acid	0.068	34.05	65.14	1.19E-03
PM01	B06	D-Gluconic Acid	0.069	37.71	63.75	9.14E-04
PM01	B07	D,L-a-Glycerol Phosphate	0.068	36.97	64.02	5.41E-04
PM01	B08	D-Xylose	0.126	65.88	56.70	3.52E-04
PM01	B09	D,L-Lactic Acid	0.067	41.14	62.60	3.87E-03
PM01	B10	Formic Acid	0.091	35.66	64.51	2.83E-03
PM01	B11	D-Mannitol	0.149	59.02	58.03	2.94E-04
PM01	B12	L-Glutamic Acid	0.058	47.61	60.71	1.02E-03
PM01	C01	D-Glucose-6-Phosphate	0.070	43.23	61.95	2.69E-02
PM01	C02	D-Galactonic Acid-g-Lactone	0.069	40.45	62.82	1.76E-02
PM01	C03	D,L-Malic Acid	0.055	72.50	55.57	4.76E-05
PM01	C04	D-Ribose	0.137	56.37	58.59	4.88E-02
PM01	C05	Tween 20	0.061	11.26	69.75	3.23E-01
PM01	C06	L-Rhamnose	0.095	43.19	61.96	1.77E-06
PM01	C07	D-Fructose	0.143	106.02	51.31	1.34E-06
PM01	C08	Acetic Acid	0.076	36.27	64.28	3.25E-04
PM01	C09	a-D-Glucose	0.151	78.86	54.60	3.96E-05
PM01	C10	Maltose	0.079	40.70	62.74	1.02E-03
PM01	C11	D-Melibiose	0.073	39.32	63.19	5.04E-03
PM01	C12	Thymidine	0.088	45.87	61.18	9.86E-03
PM01	D01	L-Asparagine	0.079	68.61	56.22	1.09E-02
PM01	D02	D-Aspartic Acid	0.027	331.22	40.39	5.56E-05
PM01	D03	D-Glucosaminic Acid	0.061	53.08	59.34	1.53E-03
PM01	D04	1,2-Propanediol	0.069	44.10	61.69	1.46E-02
PM01	D05	Tween 40	0.069	3.34	72.28	4.29E-01
PM01	D06	a-Ketoglutaric Acid	0.068	35.33	64.63	2.89E-02
PM01	D07	a-Ketobutyric Acid	0.094	65.36	56.80	3.02E-04
PM01	D08	a-Methyl-D-Galactoside	0.074	38.80	63.37	3.48E-03
PM01	D09	a-D-Lactose	0.075	38.20	63.58	4.11E-03
PM01	D10	Lactulose	0.079	35.77	64.46	3.56E-02
PM01	D11	Sucrose	0.082	35.60	64.53	3.81E-02
PM01	D12	Uridine	0.079	36.36	64.24	1.80E-02
PM01	E01	L-Glutamine	0.057	43.68	61.81	7.67E-02
PM01	E02	m-Tartaric Acid	0.062	42.72	62.10	9.00E-02
PM01	E03	D-Glucose-1-Phosphate	0.067	45.03	61.42	1.28E-02
PM01	E04	D-Fructose-6-Phosphate	0.071	39.82	63.03	3.29E-02
PM01	E05	Tween 80	0.060	57.33	58.38	3.19E-02
PM01	E06	a-Hydroxyglutaric Acid-g-Lactone	0.069	39.74	63.05	3.24E-02
PM01	E07	a-Hydroxybutyric Acid	0.077	41.09	62.61	1.58E-02
PM01	E08	b-Methyl-D-Glucoside	0.113	67.23	56.46	5.75E-04
PM01	E09	Adonitol	0.059	59.60	57.91	8.07E-03

PM01	E10	Maltotriose	0.073	40.52	62.80	1.57E-02
PM01	E11	2`-Deoxyadenosine	0.046	86.96	53.49	8.25E-03
PM01	E12	Adenosine	0.089	27.86	67.94	4.17E-01
PM01	F01	Gly-Asp	0.100	1506.51	34.88	3.62E-03
PM01	F02	Citric Acid	0.060	58.44	58.15	2.60E-02
PM01	F03	m-Inositol	0.068	62.33	57.37	2.10E-03
PM01	F04	D-Threonine	0.092	2698.58	33.98	1.04E-03
PM01	F05	Fumaric Acid	0.018	2284.45	34.26	8.73E-04
PM01	F06	Bromosuccinic Acid	0.033	614.61	35.47	8.93E-05
PM01	F07	Propionic Acid	0.068	39.50	63.14	5.51E-02
PM01	F08	Mucic Acid	0.067	37.36	63.88	2.72E-02
PM01	F09	Glycolic Acid	0.058	38.98	63.31	1.60E-03
PM01	F10	Glyoxylic Acid	0.043	80.07	54.43	6.76E-02
PM01	F11	D-Cellobiose	0.103	39.94	62.99	1.08E-01
PM01	F12	Inosine	0.075	37.95	63.67	2.44E-02
PM01	G01	Gly-Glu	0.041	366.23	39.55	5.53E-04
PM01	G02	Tricarballylic Acid	0.059	46.75	60.94	1.67E-02
PM01	G03	L-Serine	0.063	1536.86	34.85	4.48E-03
PM01	G04	L-Threonine	0.064	243.10	43.10	3.70E-03
PM01	G05	L-Alanine	0.113	2469.01	34.13	1.56E-03
PM01	G06	Ala-Gly	0.093	2129.62	34.37	3.34E-03
PM01	G07	Acetoacetic Acid	0.065	41.47	62.49	5.60E-02
PM01	G08	N-Acetyl-D-Mannosamine	0.083	40.10	62.93	3.83E-02
PM01	G09	Mono-Methylsuccinate	0.077	42.10	62.30	2.27E-02
PM01	G10	Methylpyruvate	0.079	298.88	41.27	5.31E-05
PM01	G11	D-Malic Acid	0.072	40.10	62.94	4.37E-02
PM01	G12	L-Malic Acid	0.071	40.41	62.83	3.16E-02
PM01	H01	Gly-Pro	0.077	62.14	57.40	2.84E-02
PM01	H02	p-Hydroxyphenyl Acetic Acid	0.056	77.94	54.74	8.39E-03
PM01	H03	m-Hydroxyphenyl Acetic Acid	0.067	46.56	60.99	3.51E-02
PM01	H04	Tyramine	0.049	42.65	62.13	8.13E-02
PM01	H05	D-Psicose	0.061	45.63	61.25	1.60E-02
PM01	H06	L-Lyxose	0.064	34.47	64.97	1.33E-01
PM01	H07	Glucuronamide	0.065	32.80	65.65	3.21E-02
PM01	H08	Pyruvic Acid	0.093	103.14	51.61	5.67E-04
PM01	H09	L-Galactonic Acid-g-Lactone	0.064	91.86	52.88	2.15E-04
PM01	H10	D-Galacturonic Acid	0.072	33.42	65.39	4.75E-02
PM01	H11	b-Phenylethylamine	0.048	< 2.5	75.45	9.40E-01
PM01	H12	2-Aminoethanol	0.085	173.08	46.29	1.15E-03
PM02	A01	Negative Control	0.07	32.10	65.95	9.25E-02
PM02	A02	Chondroitin Sulfate C	0.05	30.99	66.43	1.27E-01
PM02	A03	a-Cyclodextrin	0.06	34.11	65.11	1.71E-01
PM02	A04	b-Cyclodextrin	0.07	30.92	66.47	7.27E-02
PM02	A05	g-Cyclodextrin	0.06	34.39	65.00	3.00E-02
PM02	A06	Dextrin	0.05	161.86	46.95	3.99E-03
PM02	A07	Gelatin	0.07	33.13	65.51	5.62E-02
PM02	A08	Glycogen	0.07	30.70	66.57	9.94E-02
PM02	A09	Inulin	0.06	35.41	64.60	2.32E-02
PM02	A10	Laminarin	0.06	36.43	64.22	1.30E-02
PM02	A11	Mannan	0.05	39.68	63.07	1.22E-02
PM02	A12	Pectin	0.06	28.61	67.56	1.99E-01
PM02	B01	N-Acetyl-D-Galactosamine	0.07	39.06	63.28	7.94E-02
PM02	B02	N-Acetyl-Neuraminic acid	0.12	328.44	40.46	1.77E-03
PM02	B03	b-D-Allose	0.04	67.36	56.44	2.44E-02
PM02	B04	Amygdalin	0.06	39.63	63.09	3.97E-02
PM02	B05	D-Arabinose	0.07	47.32	60.78	7.23E-03
PM02	B06	D-Arabitol	0.13	89.70	53.14	1.19E-03
PM02	B07	L-Arabitol	0.10	47.18	60.82	1.21E-02
PM02	B08	Arbutin	0.13	213.05	44.31	5.36E-05
PM02	B09	2-Deoxy-D-Ribose	0.00	37.40	63.86	1.76E-02
PM02	B10	i-Erythritol	0.05	58.16	58.21	1.99E-03
PM02	B11	D-Fucose	0.07	36.83	64.07	1.05E-02
PM02	B12	3-0-b-D-Galactopyranosyl-D-	0.05	36.21	64.30	5.20E-02
PM02	C01	Gentiobiose	0.06	35.61	64.53	1.26E-01

PM02	C02	L-Glucose	0.06	42.97	62.03	1.07E-01
PM02	C03	D-Lactitol	0.07	39.36	63.18	6.52E-02
PM02	C04	D-Lyxose	0.15	139.05	48.47	3.82E-03
PM02	C05	Maltitol	0.05	49.67	60.17	3.69E-02
PM02	C06	a-Methyl-D-Glucoside	0.07	47.45	60.75	6.15E-03
PM02	C07	b-Methyl-D-Galactoside	0.06	41.75	62.40	2.58E-02
PM02	C08	3-Methylglucose	0.06	37.45	63.85	4.27E-02
PM02	C09	b-Methyl-D-Glucuronic acid	0.07	42.23	62.25	3.75E-02
PM02	C10	a-Methyl-D-Mannoside	0.11	78.77	54.61	1.18E-02
PM02	C11	b-Methyl-D-Xyloside	0.06	34.02	65.15	1.21E-01
PM02	C12	Palatinose	0.07	31.85	66.06	5.48E-02
PM02	D01	D-Raffinose	0.05	37.32	63.89	6.47E-02
PM02	D02	Salicin	0.13	140.43	48.37	3.61E-03
PM02	D03	Sedoheptulosan	0.05	47.51	60.73	1.35E-02
PM02	D04	L-Sorbose	0.04	62.07	57.42	3.14E-03
PM02	D05	Stachyose	0.06	48.78	60.40	1.10E-02
PM02	D06	D-Tagatose	0.14	120.87	49.92	8.44E-04
PM02	D07	Turanose	0.08	47.41	60.76	4.84E-03
PM02	D08	Xylitol	0.04	50.92	59.85	1.80E-03
PM02	D09	L-Xylose	0.06	42.40	62.20	1.25E-02
PM02	D10	g-Amino-N-Butyric acid	0.06	47.08	60.85	7.46E-03
PM02	D11	d-Amino Valeric acid	0.06	44.11	61.69	3.61E-02
PM02	D12	Butyric acid	0.07	41.06	62.62	2.89E-02
PM02	E01	Capric acid	0.01	162.46	46.91	3.24E-04
PM02	E02	Caproic acid	0.06	132.76	48.94	1.90E-03
PM02	E03	Citraconic acid	0.07	43.59	61.84	1.17E-02
PM02	E04	D,L-Citramalic acid	0.08	43.13	61.98	6.48E-03
PM02	E05	Dihydroxyfumaric acid	0.14	68.61	56.22	2.48E-03
PM02	E06	2-Hydroxybenzoic acid	0.04	66.49	56.59	3.20E-03
PM02	E07	4-Hydroxybenzoic acid	0.05	45.87	61.18	5.59E-03
PM02	E08	b-Hydroxybutyric acid	0.10	38.51	63.47	5.59E-03
PM02	E09	g-Hydroxybutyric acid	0.09	45.09	61.40	1.10E-03
PM02	E10	b-Hydroxypyruvic acid	0.11	66.94	56.51	1.92E-03
PM02	E11	Itaconic acid	0.03	88.94	53.24	7.28E-04
PM02	E12	5-Keto-D-Gluconic acid	0.07	4.37	71.71	3.92E-01
PM02	F01	D-Lactic acid Methyl Ester	0.06	37.22	63.93	4.68E-02
PM02	F02	Malonic acid	0.06	47.32	60.79	7.26E-03
PM02	F03	Melibionic acid	0.06	45.29	61.35	5.15E-03
PM02	F04	Oxalic acid	0.05	54.20	59.08	8.47E-03
PM02	F05	Oxalomalic acid	0.06	57.15	58.42	6.84E-03
PM02	F06	Quinic acid	0.08	49.33	60.25	2.84E-03
PM02	F07	D-Ribono-1,4-Lactone	0.05	59.98	57.83	6.98E-03
PM02	F08	Sebacic acid	0.05	53.37	59.27	2.63E-03
PM02	F09	Sorbic acid	0.00	43.15	61.97	1.64E-02
PM02	F10	Succinamic acid	0.06	51.34	59.75	3.12E-03
PM02	F11	D-Tartaric acid	0.06	46.60	60.98	4.08E-03
PM02	F12	L-Tartaric acid	0.07	30.69	66.57	1.42E-01
PM02	G01	Acetamide	0.05	37.64	63.78	4.80E-02
PM02	G02	L-Alaninamide	0.06	41.97	62.34	8.70E-03
PM02	G03	N-Acetyl-L-Glutamic acid	0.05	46.06	61.13	3.71E-03
PM02	G04	L-Arginine	0.06	39.84	63.02	5.58E-03
PM02	G05	Glycine	0.03	48.65	60.43	3.03E-02
PM02	G06	L-Histidine	0.05	51.78	59.64	5.53E-04
PM02	G07	L-Homoserine	0.01	586.65	35.82	7.04E-03
PM02	G08	Hydroxy-L-Proline	0.05	36.64	64.14	1.25E-02
PM02	G09	L-Isoleucine	0.09	85.78	53.65	2.05E-02
PM02	G10	L-Leucine	0.09	502.96	37.00	2.97E-05
PM02	G11	L-Lysine	0.07	37.35	63.88	7.95E-03
PM02	G12	L-Methionine	0.08	126.72	49.42	6.01E-04
PM02	H01	L-Ornithine	0.12	38.79	63.37	4.26E-02
PM02	H02	L-Phenylalanine	0.07	102.81	51.64	2.46E-02
PM02	H03	L-Pyroglutamic acid	0.05	33.37	65.41	5.26E-02
PM02	H04	L-Valine	0.09	135.30	48.75	1.28E-02
PM02	H05	D,L-Carnitine	0.06	32.78	65.66	4.44E-02

PM02	H06	sec-Butylamine	0.04	27.43	68.16	9.60E-02
PM02	H07	D,L-Octopamine	0.05	31.51	66.21	9.33E-02
PM02	H08	Putrescine	0.06	28.06	67.84	1.05E-01
PM02	H09	Dihydroxyacetone	0.02	30.02	66.88	1.02E-01
PM02	H10	2,3-Butanediol	0.06	33.41	65.39	6.39E-02
PM02	H11	2,3-Butanone	0.06	< 2.5	90.01	4.87E-01
PM02	H12	3-Hydroxy-2-butanone	0.07	27.21	68.28	2.17E-01
PM03	A01	Negative Control	0.04	122.22	49.80	1.90E-04
PM03	A02	Ammonia	0.05	48.51	60.47	1.87E-04
PM03	A03	Nitrite	0.03	383.40	39.17	3.24E-07
PM03	A04	Nitrate	0.04	520.86	36.73	5.81E-05
PM03	A05	Urea	0.04	433.32	38.17	1.04E-05
PM03	A06	Biuret	0.04	307.09	41.04	3.59E-06
PM03	A07	L-Alanine	0.05	83.22	53.99	7.84E-04
PM03	A08	L-Arginine	0.05	457.77	37.74	2.65E-05
PM03	A09	L-Asparagine	0.04	30.55	66.64	3.08E-03
PM03	A10	L-Aspartic acid	0.04	39.31	63.20	2.56E-04
PM03	A11	L-Cysteine	0.04	108.19	51.09	6.64E-05
PM03	A12	L-Glutamic acid	0.04	6.54	70.87	1.85E-02
PM03	B01	L-Glutamine	0.04	3.56	72.14	8.33E-02
PM03	B02	Glycine hydrochloride	0.03	31.15	66.36	2.72E-03
PM03	B03	L-Histidine	0.04	3640.19	33.40	8.27E-05
PM03	B04	L-Isoleucine	0.06	121.96	49.82	9.75E-04
PM03	B05	L-Leucine	0.05	31.80	66.07	1.34E-02
PM03	B06	L-Lysine	0.04	1766.73	34.66	3.75E-04
PM03	B07	L-Methionine	0.04	38.72	63.40	2.51E-04
PM03	B08	L-Phenylalanine	0.06	28.61	67.56	9.96E-03
PM03	B09	L-Proline	0.04	5343.38	32.54	1.07E-10
PM03	B10	L-Serine	0.03	38.66	63.42	3.98E-03
PM03	B11	L-Threonine	0.05	37.89	63.69	4.55E-03
PM03	B12	L-Tryptophan	0.03	165.15	46.75	2.91E-04
PM03	C01	L-Tyrosine	0.15	172.23	46.34	1.52E-05
PM03	C02	L-Valine	0.04	222.56	43.91	2.01E-04
PM03	C03	D-Alanine	0.04	170.66	46.43	1.70E-04
PM03	C04	D-Asparagine	0.03	195.99	45.10	2.31E-04
PM03	C05	D-Aspartic acid	0.01	3333.75	33.58	3.33E-04
PM03	C06	D-Glutamic acid	0.05	67.84	56.35	1.33E-04
PM03	C07	D-Lysine	0.05	1471.73	34.91	1.00E-04
PM03	C08	D-Serine	0.05	109.81	50.93	7.29E-05
PM03	C09	D-Valine	0.05	45.87	61.18	9.54E-03
PM03	C10	L-Citrulline	0.04	3086.32	33.73	1.02E-04
PM03	C11	L-Homoserine	0.03	119.15	50.07	2.52E-05
PM03	C12	L-Ornithine	0.04	140.60	48.36	3.49E-04
PM03	D01	N-Acetyl-L-Glutamic acid	0.03	38.53	63.47	1.47E-03
PM03	D02	N-Phthaloyl-L-Glutamic acid	0.03	136.73	48.64	4.69E-05
PM03	D03	L-Pyroglutamic acid	0.04	52.56	59.46	2.06E-04
PM03	D04	Hydroxylamine	0.02	333.76	40.33	1.58E-07
PM03	D05	Methylamine	0.04	6537.37	32.01	4.02E-05
PM03	D06	N-Amylamine	0.04	3384.22	33.55	3.74E-04
PM03	D07	N-Butylamine	0.04	5679.01	32.38	8.84E-05
PM03	D08	Ethylamine	0.04	3799.28	33.31	1.61E-04
PM03	D09	Ethanolamine	0.04	16.08	69.02	2.60E-03
PM03	D10	Ethylenediamine	0.04	4133.10	33.13	2.78E-05
PM03	D11	Putrescine	0.03	334.93	40.30	7.69E-08
PM03	D12	Agmatine	0.04	108.10	51.10	3.06E-04
PM03	E01	Histamine	0.04	492.25	37.17	3.79E-08
PM03	E02	b-Phenylethylamine	0.04	2147.06	34.36	1.80E-06
PM03	E03	Tyramine	0.04	2771.90	33.93	9.91E-07
PM03	E04	Acetamide	0.05	2925.72	33.83	1.50E-07
PM03	E05	Formamide	0.04	3638.61	33.40	7.25E-09
PM03	E06	Glucuronamide	0.04	280.16	41.84	1.10E-06
PM03	E07	D,L-Lactamide	0.04	2858.59	33.87	1.96E-11
PM03	E08	D-Glucosamine	0.04	475.75	37.43	3.86E-06
PM03	E09	D-Galactosamine	0.04	1723.23	34.69	3.43E-09

PM03	E10	D-Mannosamine	0.04	583.18	35.87	1.06E-05
PM03	E11	N-Acetyl-D-Glucosamine	0.04	44.14	61.68	2.05E-05
PM03	E12	N-Acetyl-D-Galactosamine	0.04	153.43	47.48	3.06E-04
PM03	F01	N-Acetyl-L-Glutamic acid	0.04	521.50	36.72	6.68E-09
PM03	F02	Adenine	0.03	>1680	27.58	2.10E-04
PM03	F03	Adenosine	0.05	373.18	39.39	7.02E-03
PM03	F04	Cytidine	0.05	3952.14	33.23	2.83E-09
PM03	F05	Cytosine	0.04	4808.99	32.79	1.11E-09
PM03	F06	Guanine hydrochloride	0.03	21.37	68.45	3.62E-03
PM03	F07	Guanosine	0.01	10401.96	30.58	2.45E-05
PM03	F08	Thymine	0.03	7749.29	31.52	9.78E-12
PM03	F09	Thymidine	0.04	7336.03	31.68	6.36E-09
PM03	F10	Uracil	0.04	3204.38	33.66	2.32E-04
PM03	F11	Uridine	0.04	484.06	37.30	2.62E-04
PM03	F12	Inosine	0.03	96.29	52.36	2.52E-03
PM03	G01	Xanthine	0.30	2078.68	34.41	2.83E-05
PM03	G02	Xanthosine	0.05	9498.65	30.88	3.22E-08
PM03	G03	Uric acid	0.06	4288.54	33.05	6.27E-07
PM03	G04	Alloxan	0.02	42.78	62.08	1.15E-03
PM03	G05	Allantoin	0.04	333.89	40.32	1.48E-08
PM03	G06	Parabanic acid	0.03	27.96	67.89	3.25E-03
PM03	G07	D,L-a-Amino-N-Butyric acid	0.05	65.97	56.69	3.78E-04
PM03	G08	g-Aminobutyric acid	0.04	6329.21	32.10	4.45E-11
PM03	G09	e-Amino-N-Caproic acid	0.04	3389.34	33.55	9.99E-09
PM03	G10	D,L-a-Amino-Caprylic acid	0.05	646.51	35.10	4.73E-07
PM03	G11	d-Amino-N-Valeric acid	0.04	435.03	38.14	5.08E-10
PM03	G12	a-Amino-N-Valeric acid	0.06	27.24	68.26	2.14E-02
PM03	H01	Ala-Asp	0.04	285.88	41.66	5.83E-06
PM03	H02	Ala-Gln	0.03	84.38	53.83	2.43E-03
PM03	H03	Ala-Glu	0.03	271.83	42.10	2.26E-05
PM03	H04	Ala-Gly	0.04	87.85	53.38	3.15E-03
PM03	H05	Ala-His	0.04	78.44	54.66	1.51E-03
PM03	H06	Ala-Leu	0.04	5.97	71.06	7.59E-02
PM03	H07	Ala-Thr	0.04	78.99	54.58	2.46E-03
PM03	H08	Gly-Asn	0.03	80.42	54.38	3.61E-04
PM03	H09	Gly-Gln	0.03	47.43	60.75	2.46E-03
PM03	H10	Gly-Glu	0.05	184.37	45.68	5.43E-03
PM03	H11	Gly-Met	0.04	19.30	68.66	4.24E-02
PM03	H12	Met-Ala	0.06	22.50	68.35	1.14E-01
PM04	A01	Negative Control	0.05	< 2.5	73.73	2.59E-01
PM04	A02	Phosphate	0.04	68.15	56.30	5.20E-02
PM04	A03	Pyrophosphate	0.01	< 2.5	73.96	1.98E-01
PM04	A04	Trimetaphosphate	0.05	< 2.5	74.17	1.86E-01
PM04	A05	Tripolyphosphate	0.04	3.02	72.49	1.26E-01
PM04	A06	Triethyl Phosphate	0.05	3.42	72.23	6.15E-02
PM04	A07	Hypophosphite	0.04	37.41	63.86	5.68E-03
PM04	A08	Adenosine 2`-Monophosphate	0.05	3.15	72.40	1.35E-01
PM04	A09	Adenosine 3`-Monophosphate	0.05	6.17	70.99	8.10E-02
PM04	A10	Adenosine 5`-Monophosphate	0.05	< 2.5	72.93	8.02E-02
PM04	A11	Adenosine 2`,3`-Cyclic	0.04	2.94	72.55	1.68E-01
PM04	A12	Adenosine 3`,5`-Cyclic	0.05	6.64	70.84	5.90E-02
PM04	B01	Thiophosphate	0.04	< 2.5	73.70	1.92E-01
PM04	B02	Dithiophosphate	0.04	13.34	69.40	4.16E-02
PM04	B03	D,L-a-Glycerol Phosphate	0.05	10.68	69.86	7.30E-02
PM04	B04	b-Glycerol Phosphate	0.05	5.67	71.16	7.24E-02
PM04	B05	L-a-Phosphatidyl-D,L-Glycerol	0.06	22.93	68.31	2.26E-02
PM04	B06	D-2-Phospho-Glyceric acid	0.05	27.29	68.24	2.97E-02
PM04	B07	D-3-Phospho-Glyceric acid	0.05	19.61	68.63	5.51E-02
PM04	B08	Guanosine 2`-Monophosphate	0.05	8.32	70.37	7.00E-02
PM04	B09	Guanosine 3`-Monophosphate	0.05	13.23	69.42	3.23E-02
PM04	B10	Guanosine 5`-Monophosphate	0.05	28.14	67.80	2.40E-02
PM04	B11	Guanosine 2`,3`-Cyclic	0.05	8.51	70.32	9.88E-02
PM04	B12	Guanosine 3`,5`-Cyclic	0.05	4.06	71.86	1.56E-01
PM04	C01	Phosphoenol Pyruvate	0.04	11.74	69.66	8.15E-02

PM04	C02	Phospho-Glycolic acid	0.05	17.94	68.80	7.92E-02
PM04	C03	D-Glucose-1-Phosphate	0.05	9.66	70.06	1.24E-01
PM04	C04	D-Glucose-6-Phosphate	0.05	27.55	68.10	2.75E-02
PM04	C05	2-Deoxy-D-Glucose 6-Phosphate	0.05	30.68	66.58	1.46E-02
PM04	C06	D-Glucosamine-6-Phosphate	0.05	30.43	66.69	1.60E-02
PM04	C07	6-Phospho-Gluconic acid	0.04	20.05	68.58	4.80E-02
PM04	C08	Cytidine 2`-Monophosphate	0.05	31.72	66.11	6.57E-03
PM04	C09	Cytidine 3`-Monophosphate	0.04	29.18	67.28	2.64E-02
PM04	C10	Cytidine 5`-Monophosphate	0.04	12.36	69.56	7.57E-02
PM04	C11	Cytidine 2`,3`-Cyclic Monophosphate	0.04	12.04	69.61	7.08E-02
PM04	C12	Cytidine 3`,5`-Cyclic Monophosphate	0.01	320.53	40.67	8.83E-05
PM04	D01	D-Mannose-1-Phosphate	0.03	4.92	71.46	1.41E-01
PM04	D02	D-Mannose-6-Phosphate	0.06	13.92	69.32	4.86E-02
PM04	D03	Cysteamine-S-Phosphate	0.04	14.34	69.26	3.28E-02
PM04	D04	Phospho-L-Arginine	0.05	22.53	68.35	6.51E-03
PM04	D05	O-Phospho-D-Serine	0.05	29.50	67.13	1.42E-02
PM04	D06	O-Phospho-L-Serine	0.05	20.71	68.51	1.66E-02
PM04	D07	O-Phospho-L-Threonine	0.04	13.87	69.32	3.31E-02
PM04	D08	Uridine 2`-Monophosphate	0.05	28.66	67.54	1.04E-02
PM04	D09	Uridine 3`-Monophosphate	0.05	28.90	67.42	1.08E-02
PM04	D10	Uridine 5`-Monophosphate	0.04	20.38	68.55	1.46E-02
PM04	D11	Uridine 2`,3`-Cyclic Monophosphate	0.05	16.75	68.94	3.14E-02
PM04	D12	Uridine 3`,5`-Cyclic Monophosphate	0.05	8.90	70.23	7.23E-02
PM04	E01	O-Phospho-D-Tyrosine	0.05	20.42	68.54	1.25E-01
PM04	E02	O-Phospho-L-Tyrosine	0.05	13.90	69.32	6.81E-02
PM04	E03	Phosphocreatine	0.04	30.61	66.61	2.12E-02
PM04	E04	Phosphoryl Choline	0.05	29.03	67.35	1.33E-02
PM04	E05	O-Phosphoryl-Ethanolamine	0.05	30.90	66.48	9.77E-03
PM04	E06	Phosphono Acetic acid	0.05	28.67	67.53	1.49E-02
PM04	E07	2-Aminoethyl Phosphonic acid	0.05	28.73	67.50	1.89E-02
PM04	E08	Methylene Diphosphonic acid	0.01	28.47	67.63	1.02E-02
PM04	E09	Thymidine 3`-Monophosphate	0.04	27.98	67.88	1.95E-02
PM04	E10	Thymidine 5`-Monophosphate	0.05	32.10	65.95	2.31E-02
PM04	E11	Inositol Hexaphosphate	0.04	28.31	67.71	2.79E-02
PM04	E12	Thymidine 3`,5`-Cyclic	0.05	34.23	65.06	1.91E-02
PM04	F01	Negative Control	0.04	5.65	71.17	9.25E-02
PM04	F02	Sulfate	0.05	20.75	68.51	2.14E-02
PM04	F03	Thiosulfate	0.04	31.00	66.43	1.22E-02
PM04	F04	Tetrathionate	0.04	21.93	68.40	2.09E-02
PM04	F05	Thiophosphate	0.04	29.89	66.94	7.94E-03
PM04	F06	Dithiophosphate	0.05	21.66	68.43	1.99E-02
PM04	F07	L-Cysteine	0.04	28.70	67.52	8.82E-03
PM04	F08	D-Cysteine	0.04	28.34	67.69	9.01E-03
PM04	F09	Cys-Gly	0.04	31.80	66.08	1.12E-02
PM04	F10	L-Cysteic acid	0.05	31.93	66.02	1.31E-02
PM04	F11	Cysteamine	0.04	29.42	67.17	2.85E-02
PM04	F12	L-Cysteine Sulfinic acid	0.04	3.99	71.90	1.53E-01
PM04	G01	N-Acetyl-L-Cysteine	0.05	6.40	70.91	1.52E-01
PM04	G02	S-Methyl-L-Cysteine	0.04	10.51	69.89	3.47E-02
PM04	G03	Cystathionine	0.05	27.92	67.91	2.53E-02
PM04	G04	Lanthionine	0.04	10.94	69.81	4.58E-02
PM04	G05	Glutathione	0.04	19.38	68.65	2.20E-02
PM04	G06	D,L-Ethionine	0.05	7.16	70.68	4.88E-02
PM04	G07	L-Methionine	0.05	14.64	69.21	3.28E-02
PM04	G08	D-Methionine	0.04	21.78	68.41	1.86E-02
PM04	G09	Gly-Met	0.05	29.45	67.15	1.06E-02
PM04	G10	N-Acetyl-D,L-Methionine	0.05	15.19	69.14	3.56E-02
PM04	G11	L-Methionine Sulfoxide	0.05	10.94	69.81	4.96E-02
PM04	G12	L-Methionine Sulfone	0.04	< 2.5	76.05	3.84E-01
PM04	H01	L-Djenkolic acid	0.05	3.12	72.42	1.23E-01
PM04	H02	Thiourea	0.04	2.93	72.55	1.27E-01
PM04	H03	1-Thio-b-D-Glucose	0.04	5.30	71.30	7.50E-02
PM04	H04	D,L-Lipoamide	0.03	< 2.5	73.39	1.16E-01
PM04	H05	Taurocholic acid	0.05	< 2.5	73.30	1.64E-01

PM04	H06	Taurine	0.05	< 2.5	73.95	1.76E-01
PM04	H07	Hypotaurine	0.04	< 2.5	75.59	3.83E-01
PM04	H08	p-Aminobenzene Sulfonic acid	0.05	< 2.5	73.90	1.64E-01
PM04	H09	Butane Sulfonic acid	0.04	< 2.5	75.35	3.37E-01
PM04	H10	2-Hydroxyethane Sulfonic acid	0.04	< 2.5	73.47	2.03E-01
PM04	H11	Methane Sulfonic acid	0.05	2.86	72.61	1.72E-01
PM04	H12	Tetramethylene Sulfone	0.04	< 2.5	77.63	6.03E-01
PM05	A01	Negative Control	0.05	12.23	69.58	3.94E-01
PM05	A02	Positive Control	0.05	29.13	67.30	2.43E-01
PM05	A03	L-Alanine	0.04	29.49	67.13	2.32E-01
PM05	A04	L-Arginine	0.03	28.21	67.76	2.41E-01
PM05	A05	L-Asparagine	0.04	29.27	67.24	1.79E-01
PM05	A06	L-Aspartic acid	0.05	14.17	69.28	2.48E-01
PM05	A07	L-Cysteine	0.04	27.31	68.23	2.31E-01
PM05	A08	L-Glutamic acid	0.05	18.28	68.77	2.30E-01
PM05	A09	Adenosine 3`,5`-Cyclic	0.04	11.08	69.78	3.11E-01
PM05	A10	Adenine	0.05	5.39	71.27	3.60E-01
PM05	A11	Adenosine	0.04	< 2.5	73.76	5.88E-01
PM05	A12	2`-Deoxyadenosine	0.05	12.08	69.61	3.57E-01
PM05	B01	L-Glutamine	0.04	17.80	68.82	3.55E-01
PM05	B02	Glycine	0.05	29.16	67.29	2.34E-01
PM05	B03	L-Histidine	0.04	30.22	66.79	1.94E-01
PM05	B04	L-Isoleucine	0.04	27.53	68.11	2.23E-01
PM05	B05	L-Leucine	0.04	28.56	67.58	1.63E-01
PM05	B06	L-Lysine	0.04	14.77	69.20	2.29E-01
PM05	B07	L-Methionine	0.04	29.86	66.96	1.48E-01
PM05	B08	L-Phenylalanine	0.04	33.61	65.31	1.16E-01
PM05	B09	Guanosine 3`,5`-Cyclic	0.04	30.23	66.78	1.48E-01
PM05	B10	Guanine	0.03	16.80	68.94	1.99E-01
PM05	B11	Guanosine	0.05	12.14	69.60	2.69E-01
PM05	B12	2`-Deoxyguanosine	0.05	27.19	68.29	3.07E-01
PM05	C01	L-Proline	0.04	12.43	69.55	4.03E-01
PM05	C02	L-Serine	0.04	29.88	66.95	1.41E-01
PM05	C03	L-Threonine	0.04	28.53	67.60	1.77E-01
PM05	C04	L-Tryptophan	0.04	30.25	66.78	1.38E-01
PM05	C05	L-Tyrosine	0.05	30.97	66.45	1.28E-01
PM05	C06	L-Valine	0.04	29.83	66.97	1.53E-01
PM05	C07	L-Isoleucine + L-Valine	0.05	46.67	60.96	2.48E-01
PM05	C08	Hydroxy-L-Proline	0.04	32.58	65.74	1.38E-01
PM05	C09	(5) 4-Amino-Imidazole-4(5)-	0.05	29.86	66.95	9.77E-02
PM05	C10	Hypoxanthine	0.05	28.15	67.79	1.71E-01
PM05	C11	Inosine	0.04	10.44	69.90	2.82E-01
PM05	C12	2`-Deoxyinosine	0.05	15.60	69.09	3.85E-01
PM05	D01	L-Ornithine	0.05	28.59	67.57	3.52E-01
PM05	D02	L-Citrulline	0.04	34.08	65.12	1.57E-01
PM05	D03	Chorismic acid	0.04	28.61	67.56	1.89E-01
PM05	D04	(-)Shikimic acid	0.04	33.40	65.40	1.49E-01
PM05	D05	L-Homoserine Lactone	0.05	32.87	65.62	1.06E-01
PM05	D06	D-Alanine	0.05	29.06	67.34	1.66E-01
PM05	D07	D-Aspartic acid	0.05	30.39	66.71	1.59E-01
PM05	D08	D-Glutamic acid	0.05	29.12	67.31	1.87E-01
PM05	D09	D,L-Diamino-a,e-Pimelic acid	0.04	28.69	67.52	1.64E-01
PM05	D10	Cytosine	0.04	14.24	69.27	2.02E-01
PM05	D11	Cytidine	0.04	10.53	69.89	3.23E-01
PM05	D12	2`-Deoxycytidine	0.04	10.10	69.97	4.05E-01
PM05	E01	Putrescine	0.03	14.46	69.24	3.99E-01
PM05	E02	Spermidine	0.04	31.69	66.12	1.67E-01
PM05	E03	Spermine	0.04	29.75	67.01	1.58E-01
PM05	E04	Pyridoxine	0.04	34.34	65.02	1.21E-01
PM05	E05	Pyridoxal	0.04	31.93	66.02	1.12E-01
PM05	E06	Pyridoxamine	0.04	29.44	67.16	1.85E-01
PM05	E07	b-Alanine	0.04	32.22	65.89	1.44E-01
PM05	E08	D-Pantothenic acid	0.05	30.97	66.44	1.40E-01
PM05	E09	Orotic acid	0.04	30.90	66.47	1.62E-01

PM05	E10	Uracil	0.05	28.88	67.43	2.03E-01
PM05	E11	Uridine	0.05	16.19	69.01	2.48E-01
PM05	E12	2`-Deoxyuridine	0.05	13.26	69.42	3.49E-01
PM05	F01	Quinolinic acid	0.04	15.69	69.07	3.88E-01
PM05	F02	Nicotinic acid	0.04	32.23	65.89	1.82E-01
PM05	F03	Nicotinamide	0.01	28.34	67.70	1.82E-01
PM05	F04	b-Nicotinamide Adenine Dinucleotide	0.04	21.12	68.48	1.47E-01
PM05	F05	d-Amino-N-Valeric acid	0.03	31.40	66.25	1.32E-01
PM05	F06	Hematin	0.05	30.58	66.62	1.14E-01
PM05	F07	Deferoxamine	0.04	31.09	66.39	1.34E-01
PM05	F08	a-D-Glucose	0.05	31.37	66.27	1.14E-01
PM05	F09	N-Acetyl-D-Glucosamine	0.04	31.24	66.32	1.59E-01
PM05	F10	Thymine	0.05	30.08	66.85	1.51E-01
PM05	F11	Glutathione	0.05	20.23	68.56	2.90E-01
PM05	F12	Thymidine	0.05	19.60	68.63	2.77E-01
PM05	G01	Oxaloacetic acid	0.04	32.90	65.61	2.59E-01
PM05	G02	D-Biotin	0.04	33.25	65.46	2.00E-01
PM05	G03	Cyanocobalamin	0.02	31.85	66.06	1.62E-01
PM05	G04	p-Amino-Benzoic acid	0.04	27.64	68.05	2.42E-01
PM05	G05	Folic acid	0.04	30.58	66.62	1.86E-01
PM05	G06	Inosine + Thiamine	0.04	29.86	66.95	1.80E-01
PM05	G07	Thiamine	0.03	30.16	66.82	2.01E-01
PM05	G08	Thiamine Pyrophosphate	0.04	28.95	67.39	1.72E-01
PM05	G09	Riboflavin	0.05	30.50	66.66	1.27E-01
PM05	G10	Pyrrolo-Quinoline Quinone	0.04	28.06	67.84	1.69E-01
PM05	G11	Menadione	0.05	11.18	69.76	2.91E-01
PM05	G12	m-Inositol	0.05	13.43	69.39	3.57E-01
PM05	H01	Butyric acid	0.05	28.29	67.72	4.25E-01
PM05	H02	a-Hydroxybutyric acid	0.04	6.80	70.79	5.17E-01
PM05	H03	a-Ketobutyric acid	0.04	10.93	69.81	4.61E-01
PM05	H04	Caprylic acid	0.01	10.32	69.93	4.77E-01
PM05	H05	D,L-Thioctic acid	0.03	16.53	68.97	4.40E-01
PM05	H06	D,L-Mevalonic acid Lactone	0.04	7.17	70.68	4.89E-01
PM05	H07	D,L-Carnitine	0.05	7.05	70.71	4.92E-01
PM05	H08	Choline	0.04	6.52	70.87	4.76E-01
PM05	H09	Tween 20	0.03	31.76	66.09	3.66E-01
PM05	H10	Tween 40	0.05	3.60	72.12	5.52E-01
PM05	H11	Tween 60	0.04	4.56	71.62	5.20E-01
PM05	H12	Tween 80	0.05	12.48	69.54	4.71E-01
PM06	A01	Negative Control	0.05	54.75	58.95	2.35E-03
PM06	A02	L-Glutamine	0.04	2.92	72.56	8.45E-02
PM06	A03	Ala-Ala	0.04	33.19	65.49	1.60E-01
PM06	A04	Ala-Arg	0.05	33.88	65.20	1.30E-03
PM06	A05	Ala-Asn	0.05	21.84	68.41	1.76E-02
PM06	A06	Ala-Glu	0.05	209.45	44.47	1.65E-04
PM06	A07	Ala-Gly	0.05	28.87	67.43	3.94E-02
PM06	A08	Ala-His	0.04	21.00	68.49	6.68E-03
PM06	A09	Ala-Leu	0.05	9.94	70.00	3.04E-02
PM06	A10	Ala-Lys	0.05	15.27	69.13	7.77E-02
PM06	A11	Ala-Phe	0.05	6.00	71.05	1.07E-01
PM06	A12	Ala-Pro	0.05	< 2.5	73.39	2.44E-01
PM06	B01	Ala-Ser	0.06	5.00	71.43	8.60E-02
PM06	B02	Ala-Thr	0.05	174.64	46.20	1.49E-03
PM06	B03	Ala-Trp	0.05	59.34	57.96	9.32E-05
PM06	B04	Ala-Tyr	0.05	36.73	64.11	5.58E-04
PM06	B05	Arg-Ala	0.05	10080.20	30.69	2.32E-07
PM06	B06	Arg-Arg	0.04	8228.79	31.34	4.65E-07
PM06	B07	Arg-Asp	0.04	10370.66	30.59	5.70E-08
PM06	B08	Arg-Gln	0.05	8911.74	31.09	4.54E-08
PM06	B09	Arg-Glu	0.04	4059.17	33.17	1.70E-09
PM06	B10	Arg-Ile	0.05	281.90	41.78	4.24E-05
PM06	B11	Arg-Leu	0.04	53.88	59.15	4.12E-03
PM06	B12	Arg-Lys	0.04	307.72	41.02	6.88E-04
PM06	C01	Arg-Met	0.05	531.52	36.57	1.53E-05

PM06	C02	Arg-Phe	0.05	6541.35	32.01	7.07E-05
PM06	C03	Arg-Ser	0.04	7608.06	31.58	7.81E-07
PM06	C04	Arg-Trp	0.05	8423.31	31.27	1.85E-08
PM06	C05	Arg-Tyr	0.05	8818.65	31.12	2.70E-07
PM06	C06	Arg-Val	0.05	32.09	65.95	2.39E-03
PM06	C07	Asn-Glu	0.04	35.96	64.39	2.54E-03
PM06	C08	Asn-Val	0.06	39.47	63.14	6.69E-04
PM06	C09	Asp-Asp	0.07	29.20	67.27	1.53E-03
PM06	C10	Asp-Glu	0.05	17.61	68.84	1.27E-02
PM06	C11	Asp-Leu	0.04	417.47	38.47	6.28E-06
PM06	C12	Asp-Lys	0.07	5.22	71.34	6.25E-02
PM06	D01	Asp-Phe	0.05	9.87	70.02	4.44E-02
PM06	D02	Asp-Trp	0.05	75.56	55.09	1.61E-03
PM06	D03	Asp-Val	0.05	36.08	64.35	5.58E-04
PM06	D04	Cys-Gly	0.05	48.40	60.50	5.63E-05
PM06	D05	Gln-Gln	0.03	37.89	63.69	4.62E-04
PM06	D06	Gln-Gly	0.04	32.96	65.58	1.59E-04
PM06	D07	Glu-Asp	0.05	29.08	67.33	9.87E-03
PM06	D08	Glu-Glu	0.04	27.68	68.03	8.48E-03
PM06	D09	Glu-Gly	0.04	12.85	69.48	1.64E-02
PM06	D10	Glu-Ser	0.05	8.36	70.36	1.68E-02
PM06	D11	Glu-Trp	0.04	8.48	70.33	1.99E-02
PM06	D12	Glu-Tyr	0.05	3.37	72.26	8.99E-02
PM06	E01	Glu-Val	0.04	32.41	65.81	2.02E-01
PM06	E02	Gly-Ala	0.06	32.17	65.92	1.62E-03
PM06	E03	Gly-Arg	0.04	351.89	39.88	6.87E-05
PM06	E04	Gly-Cys	0.05	33.81	65.23	1.91E-03
PM06	E05	Gly-Gly	0.05	38.90	63.34	2.17E-03
PM06	E06	Gly-His	0.03	39.92	62.99	5.85E-03
PM06	E07	Gly-Leu	0.05	45.22	61.37	4.36E-02
PM06	E08	Gly-Lys	0.05	36.85	64.06	2.04E-04
PM06	E09	Gly-Met	0.05	27.50	68.13	6.97E-03
PM06	E10	Gly-Phe	0.06	28.82	67.46	7.05E-03
PM06	E11	Gly-Pro	0.04	3797.99	33.32	9.96E-07
PM06	E12	Gly-Ser	0.05	< 2.5	75.67	3.13E-01
PM06	F01	Gly-Thr	0.05	4.07	71.86	1.14E-01
PM06	F02	Gly-Trp	0.04	3631.87	33.41	1.68E-05
PM06	F03	Gly-Tyr	0.04	42.25	62.25	2.35E-04
PM06	F04	Gly-Val	0.05	31.82	66.07	1.55E-03
PM06	F05	His-Asp	0.04	40.63	62.76	2.99E-04
PM06	F06	His-Gly	0.04	40.57	62.78	4.67E-03
PM06	F07	His-Leu	0.05	375.71	39.34	6.77E-05
PM06	F08	His-Lys	0.05	6992.53	31.82	1.02E-07
PM06	F09	His-Met	0.05	36.99	64.01	7.02E-04
PM06	F10	His-Pro	0.05	8568.12	31.22	6.08E-07
PM06	F11	His-Ser	0.05	34.15	65.10	3.27E-03
PM06	F12	His-Trp	0.04	178.38	46.00	1.49E-04
PM06	G01	His-Tyr	0.04	45.27	61.35	4.98E-03
PM06	G02	His-Val	0.05	30.46	66.68	3.10E-03
PM06	G03	Ile-Ala	0.05	30.09	66.85	5.70E-03
PM06	G04	Ile-Arg	0.04	122.30	49.79	3.82E-03
PM06	G05	Ile-Gln	0.05	29.13	67.30	1.82E-03
PM06	G06	Ile-Gly	0.05	28.84	67.45	1.14E-03
PM06	G07	Ile-His	0.03	29.29	67.23	6.63E-03
PM06	G08	Ile-Ile	0.05	27.63	68.05	1.76E-02
PM06	G09	Ile-Met	0.05	29.65	67.06	1.90E-03
PM06	G10	Ile-Phe	0.05	20.85	68.50	6.51E-03
PM06	G11	Ile-Pro	0.04	13.58	69.37	9.00E-03
PM06	G12	Ile-Ser	0.05	4.64	71.58	5.35E-02
PM06	H01	Ile-Trp	0.04	30.04	66.87	4.39E-02
PM06	H02	Ile-Tyr	0.03	31.43	66.24	1.99E-02
PM06	H03	Ile-Val	0.05	19.16	68.67	2.93E-02
PM06	H04	Leu-Ala	0.05	32.51	65.77	1.91E-02
PM06	H05	Leu-Arg	0.04	67.41	56.43	5.99E-03

PM06	H06	Leu-Asp	0.04	483.20	37.31	2.59E-06
PM06	H07	Leu-Glu	0.04	35.89	64.42	1.11E-01
PM06	H08	Leu-Gly	0.05	31.07	66.40	1.14E-01
PM06	H09	Leu-Ile	0.05	< 2.5	73.29	1.26E-01
PM06	H10	Leu-Leu	0.05	< 2.5	74.14	1.18E-01
PM06	H11	Leu-Met	0.05	< 2.5	74.38	7.05E-02
PM06	H12	Leu-Phe	0.05	< 2.5	74.54	5.03E-02
PM07	A01	Negative Control	0.05	45.51	61.28	2.68E-03
PM07	A02	L-Glutamine	0.03	< 2.5	73.04	7.04E-02
PM07	A03	Leu-Ser	0.04	115.79	50.37	1.51E-04
PM07	A04	Leu-Trp	0.04	< 2.5	73.17	9.96E-02
PM07	A05	Leu-Val	0.04	31.14	66.37	7.60E-04
PM07	A06	Lys-Ala	0.05	15.99	69.04	1.25E-02
PM07	A07	Lys-Arg	0.05	5535.64	32.45	9.53E-07
PM07	A08	Lys-Glu	0.04	271.32	42.12	1.01E-04
PM07	A09	Lys-Ile	0.03	6.94	70.74	2.61E-02
PM07	A10	Lys-Leu	0.04	3.03	72.48	3.95E-02
PM07	A11	Lys-Lys	0.04	149.66	47.73	5.07E-05
PM07	A12	Lys-Phe	0.05	< 2.5	72.99	6.40E-02
PM07	B01	Lys-Pro	0.04	656.96	34.98	4.05E-05
PM07	B02	Lys-Ser	0.04	5104.94	32.65	5.29E-06
PM07	B03	Lys-Thr	0.04	20.25	68.56	4.95E-03
PM07	B04	Lys-Trp	0.01	5276.60	32.57	1.25E-07
PM07	B05	Lys-Tyr	0.05	63.98	57.05	1.30E-05
PM07	B06	Lys-Val	0.04	28.72	67.50	1.63E-03
PM07	B07	Met-Arg	0.04	37.99	63.65	1.16E-03
PM07	B08	Met-Asp	0.05	93.94	52.63	1.04E-05
PM07	B09	Met-Gln	0.04	28.50	67.61	4.56E-03
PM07	B10	Met-Glu	0.04	89.17	53.21	2.78E-04
PM07	B11	Met-Gly	0.05	7.91	70.47	1.32E-02
PM07	B12	Met-His	0.05	11.89	69.64	8.27E-03
PM07	C01	Met-Ile	0.05	8.31	70.37	5.52E-02
PM07	C02	Met-Leu	0.04	9.60	70.08	1.62E-02
PM07	C03	Met-Lys	0.05	30.38	66.72	2.03E-03
PM07	C04	Met-Met	0.04	28.23	67.75	6.32E-03
PM07	C05	Met-Phe	0.04	30.35	66.73	2.18E-03
PM07	C06	Met-Pro	0.05	34.39	65.00	1.45E-03
PM07	C07	Met-Trp	0.04	29.66	67.05	2.46E-03
PM07	C08	Met-Val	0.05	44.95	61.44	4.53E-02
PM07	C09	Phe-Ala	0.04	20.26	68.56	2.85E-03
PM07	C10	Phe-Gly	0.05	18.42	68.75	9.96E-03
PM07	C11	Phe-Ile	0.04	10.05	69.98	3.66E-02
PM07	C12	Phe-Phe	0.05	5.07	71.40	1.05E-02
PM07	D01	Phe-Pro	0.04	7.69	70.53	4.81E-02
PM07	D02	Phe-Ser	0.06	18.50	68.74	6.06E-03
PM07	D03	Phe-Trp	0.05	32.73	65.68	2.39E-04
PM07	D04	Pro-Ala	0.01	22.50	68.35	1.81E-03
PM07	D05	Pro-Asp	0.04	36.19	64.31	1.88E-03
PM07	D06	Pro-Gln	0.03	15.53	69.09	8.14E-03
PM07	D07	Pro-Gly	0.05	67.03	56.50	1.45E-04
PM07	D08	Pro-Hyp	0.04	7523.26	31.61	1.04E-06
PM07	D09	Pro-Leu	0.05	33.78	65.25	3.15E-04
PM07	D10	Pro-Phe	0.05	30.78	66.53	1.45E-03
PM07	D11	Pro-Pro	0.04	634.01	35.24	3.64E-06
PM07	D12	Pro-Tyr	0.05	29.32	67.21	1.32E-03
PM07	E01	Ser-Ala	0.04	7.60	70.56	5.95E-02
PM07	E02	Ser-Gly	0.06	22.08	68.39	1.28E-02
PM07	E03	Ser-His	0.04	35.77	64.47	5.86E-04
PM07	E04	Ser-Leu	0.04	187.50	45.52	3.43E-04
PM07	E05	Ser-Met	0.05	32.33	65.85	1.67E-03
PM07	E06	Ser-Phe	0.07	29.20	67.27	1.97E-03
PM07	E07	Ser-Pro	0.05	7135.72	31.76	2.23E-07
PM07	E08	Ser-Ser	0.06	9.24	70.15	1.32E-02
PM07	E09	Ser-Tyr	0.05	32.27	65.87	4.18E-03

PM07	E10	Ser-Val	0.06	8.44	70.34	1.97E-02
PM07	E11	Thr-Ala	0.05	8.67	70.28	4.67E-03
PM07	E12	Thr-Arg	0.05	46.93	60.89	3.86E-03
PM07	F01	Thr-Glu	0.05	60.81	57.66	1.29E-02
PM07	F02	Thr-Gly	0.05	8.62	70.30	2.88E-02
PM07	F03	Thr-Leu	0.05	66.06	56.67	2.28E-05
PM07	F04	Thr-Met	0.04	9.47	70.10	1.96E-02
PM07	F05	Thr-Pro	0.04	4771.57	32.81	3.32E-06
PM07	F06	Trp-Ala	0.05	414.87	38.53	1.08E-04
PM07	F07	Trp-Arg	0.06	6468.04	32.04	5.20E-06
PM07	F08	Trp-Asp	0.05	77.50	54.80	1.54E-07
PM07	F09	Trp-Glu	0.04	5.93	71.07	1.82E-02
PM07	F10	Trp-Gly	0.05	618.89	35.42	7.40E-07
PM07	F11	Trp-Leu	0.03	27.18	68.29	6.31E-02
PM07	F12	Trp-Lys	0.06	492.11	37.17	1.67E-05
PM07	G01	Trp-Phe	0.04	28.76	67.49	4.49E-02
PM07	G02	Trp-Ser	0.05	479.58	37.37	1.63E-04
PM07	G03	Trp-Trp	0.05	4103.86	33.15	1.74E-03
PM07	G04	Trp-Tyr	0.05	133.77	48.86	5.89E-05
PM07	G05	Tyr-Ala	0.03	186.36	45.58	5.79E-05
PM07	G06	Tyr-Gln	0.04	30.91	66.47	1.63E-02
PM07	G07	Tyr-Glu	0.04	84.36	53.83	1.65E-02
PM07	G08	Tyr-Gly	0.04	36.90	64.04	3.98E-04
PM07	G09	Tyr-His	0.05	63.10	57.22	4.65E-05
PM07	G10	Tyr-Leu	0.06	3.91	71.94	1.60E-02
PM07	G11	Tyr-Lys	0.05	29.80	66.98	1.99E-03
PM07	G12	Tyr-Phe	0.06	< 2.5	73.21	7.37E-02
PM07	H01	Tyr-Trp	0.05	537.03	36.49	1.19E-03
PM07	H02	Tyr-Tyr	0.04	174.42	46.22	2.16E-04
PM07	H03	Val-Arg	0.04	35.65	64.51	2.36E-03
PM07	H04	Val-Asn	0.06	72.38	55.59	5.83E-04
PM07	H05	Val-Asp	0.05	224.68	43.82	1.12E-04
PM07	H06	Val-Gly	0.06	3.61	72.11	4.63E-02
PM07	H07	Val-His	0.05	5.13	71.37	6.87E-02
PM07	H08	Val-Ile	0.05	3.70	72.06	7.19E-02
PM07	H09	Val-Leu	0.04	15.27	69.13	3.23E-03
PM07	H10	Val-Tyr	0.05	6.70	70.82	2.60E-02
PM07	H11	Val-Val	0.05	4.26	71.76	3.62E-02
PM07	H12	g-Glu-Gly	0.05	29.82	66.97	1.62E-01
PM08	A01	Negative Control	0.05	73.74	55.38	1.63E-02
PM08	A02	L-Glutamine	0.04	3.97	71.91	9.00E-02
PM08	A03	Ala-Asp	0.04	210.90	44.41	8.69E-05
PM08	A04	Ala-Gln	0.03	3.77	72.02	3.93E-02
PM08	A05	Ala-Ile	0.05	14.12	69.29	4.17E-02
PM08	A06	Ala-Met	0.04	5.66	71.17	7.86E-02
PM08	A07	Ala-Val	0.05	10.91	69.81	2.52E-02
PM08	A08	Asp-Ala	0.05	15.12	69.15	1.38E-02
PM08	A09	Asp-Gln	0.04	27.30	68.23	8.81E-03
PM08	A10	Asp-Gly	0.05	13.39	69.40	2.72E-02
PM08	A11	Glu-Ala	0.04	40.01	62.97	4.31E-04
PM08	A12	Gly-Asn	0.04	36.98	64.01	6.24E-03
PM08	B01	Gly-Asp	0.06	10.95	69.81	1.21E-01
PM08	B02	Gly-Ile	0.04	16.14	69.02	2.61E-02
PM08	B03	His-Ala	0.05	29.40	67.17	1.21E-02
PM08	B04	His-Glu	0.04	21.18	68.47	9.26E-03
PM08	B05	His-His	0.02	36.10	64.34	5.24E-03
PM08	B06	Ile-Asn	0.04	38.61	63.44	5.43E-03
PM08	B07	Ile-Leu	0.04	88.42	53.30	1.13E-03
PM08	B08	Leu-Asn	0.05	380.69	39.23	2.24E-03
PM08	B09	Leu-His	0.04	39.00	63.31	3.09E-03
PM08	B10	Leu-Pro	0.05	8.36	70.36	3.86E-02
PM08	B11	Leu-Tyr	0.05	4.48	71.66	7.48E-02
PM08	B12	Lys-Asp	0.05	335.07	40.29	4.93E-05
PM08	C01	Lys-Gly	0.05	1949.71	34.51	1.30E-04

PM08	C02	Lys-Met	0.07	19.98	68.59	5.26E-02
PM08	C03	Met-Thr	0.04	5.19	71.35	2.18E-02
PM08	C04	Met-Tyr	0.04	19.08	68.68	7.56E-03
PM08	C05	Phe-Asp	0.06	40.79	62.71	3.64E-04
PM08	C06	Phe-Glu	0.05	38.05	63.63	7.19E-04
PM08	C07	Gln-Glu	0.04	31.75	66.10	1.11E-03
PM08	C08	Phe-Met	0.05	17.28	68.88	4.32E-03
PM08	C09	Phe-Tyr	0.03	27.36	68.20	1.45E-02
PM08	C10	Phe-Val	0.05	8.81	70.25	5.67E-02
PM08	C11	Pro-Arg	0.04	2967.35	33.80	2.61E-03
PM08	C12	Pro-Asn	0.04	58.86	58.06	7.74E-04
PM08	D01	Pro-Glu	0.04	3.49	72.19	1.05E-01
PM08	D02	Pro-Ile	0.04	27.95	67.89	8.54E-03
PM08	D03	Pro-Lys	0.04	550.03	36.31	1.74E-06
PM08	D04	Pro-Ser	0.04	27.93	67.90	1.08E-03
PM08	D05	Pro-Trp	0.04	643.21	35.14	1.92E-05
PM08	D06	Pro-Val	0.06	37.74	63.74	8.25E-05
PM08	D07	Ser-Asn	0.04	27.52	68.11	2.83E-03
PM08	D08	Ser-Asp	0.06	43.61	61.83	2.59E-05
PM08	D09	Ser-Gln	0.04	7.08	70.70	9.54E-03
PM08	D10	Ser-Glu	0.05	45.47	61.30	1.35E-02
PM08	D11	Thr-Asp	0.06	51.83	59.63	1.83E-03
PM08	D12	Thr-Gln	0.04	< 2.5	73.63	7.74E-02
PM08	E01	Thr-Phe	0.06	4.32	71.73	9.89E-02
PM08	E02	Thr-Ser	0.05	30.34	66.73	3.72E-03
PM08	E03	Trp-Val	0.03	184.00	45.70	1.26E-04
PM08	E04	Tyr-Ile	0.04	27.42	68.16	3.20E-03
PM08	E05	Tyr-Val	0.04	48.97	60.35	5.39E-02
PM08	E06	Val-Ala	0.04	42.23	62.25	7.82E-02
PM08	E07	Val-Gln	0.05	31.60	66.16	1.07E-03
PM08	E08	Val-Glu	0.05	178.34	46.00	1.11E-05
PM08	E09	Val-Lys	0.05	17.64	68.84	9.54E-03
PM08	E10	Val-Met	0.05	28.56	67.58	1.60E-02
PM08	E11	Val-Phe	0.04	8.27	70.38	1.36E-02
PM08	E12	Val-Pro	0.04	183.39	45.73	2.01E-04
PM08	F01	Val-Ser	0.05	< 2.5	75.50	6.23E-01
PM08	F02	b-Ala-Ala	0.03	28.08	67.83	9.87E-03
PM08	F03	b-Ala-Gly	0.04	1546.56	34.84	2.32E-07
PM08	F04	b-Ala-His	0.05	8264.53	31.33	3.15E-06
PM08	F05	Met-b-Ala	0.06	29.02	67.36	2.10E-03
PM08	F06	b-Ala-Phe	0.04	15.44	69.11	9.04E-03
PM08	F07	D-Ala-D-Ala	0.05	20.02	68.58	5.83E-03
PM08	F08	D-Ala-Gly	0.05	125.78	49.50	4.70E-05
PM08	F09	D-Ala-Leu	0.05	66.51	56.59	1.94E-05
PM08	F10	D-Leu-D-Leu	0.04	32.79	65.65	3.75E-02
PM08	F11	D-Leu-Gly	0.04	12.51	69.53	2.97E-02
PM08	F12	D-Leu-Tyr	0.03	129.16	49.23	2.81E-05
PM08	G01	g-Glu-Gly	0.05	17.61	68.84	4.94E-02
PM08	G02	g-D-Glu-Gly	0.05	10122.13	30.67	4.82E-07
PM08	G03	Gly-D-Ala	0.05	27.15	68.31	1.42E-02
PM08	G04	Gly-D-Asp	0.05	1537.16	34.85	3.45E-07
PM08	G05	Gly-D-Ser	0.05	297.47	41.31	8.49E-06
PM08	G06	Gly-D-Thr	0.05	156.62	47.27	3.21E-05
PM08	G07	Gly-D-Val	0.04	38.41	63.51	1.12E-01
PM08	G08	Leu-b-Ala	0.04	11.77	69.66	5.61E-03
PM08	G09	Leu-D-Leu	0.05	34.37	65.01	1.35E-03
PM08	G10	Phe-b-Ala	0.06	10.26	69.94	2.05E-02
PM08	G11	Ala-Ala-Ala	0.05	51.70	59.66	2.21E-05
PM08	G12	D-Ala-Gly-Gly	0.05	514.90	36.82	1.09E-05
PM08	H01	Gly-Gly-Ala	0.05	36.36	64.24	2.02E-02
PM08	H02	Gly-Gly-D-Leu	0.04	11.73	69.67	2.23E-01
PM08	H03	Gly-Gly-Gly	0.05	1810.79	34.62	2.93E-05
PM08	H04	Gly-Gly-Ile	0.04	< 2.5	73.68	1.38E-01
PM08	H05	Gly-Gly-Leu	0.03	214.74	44.24	1.03E-04

PM08	H06	Gly-Gly-Phe	0.05	10.40	69.91	1.38E-02
PM08	H07	Val-Tyr-Val	0.04	295.94	41.36	2.62E-06
PM08	H08	Gly-Phe-Phe	0.28	298.09	41.30	4.18E-07
PM08	H09	Leu-Gly-Gly	0.04	11.13	69.77	3.01E-01
PM08	H10	Leu-Leu-Leu	0.05	< 2.5	73.75	2.32E-01
PM08	H11	Phe-Gly-Gly	0.05	32.50	65.78	1.33E-03
PM08	H12	Tyr-Gly-Gly	0.06	39.10	63.27	2.52E-04

a. OD (750nm) differences between *C. difficile* under certain PM substrate and the same substrate without *C. difficile* .

b. Toxin concentrations in *C. difficile* supernatant collected from different PM conditions, which were calculaated from the average dye reduction rate by the CHO-k1 cells according to the equations in Table 1.

c. Product of an actual dye reduction rate of CHO-k1 cells with *C. difficile* supernatant collected from a PM substrate and the ratio of the same PM substrate dye reduction rate (see Material and Methods).

d. The P values were obtained from t-test on the dye reduction rates of CHO-k1 cells in the presence or absence of *C. difficile* supernatants collected from different PM conditions.
